# Identification of cuproptosis-related lncRNAs signature for predicting the prognosis in patients with kidney renal clear cell carcinoma

**DOI:** 10.1016/j.jgeb.2023.100338

**Published:** 2024-01-30

**Authors:** Ya He, Hongxia Zhang, Jingang Li, Hui Zhou, Fei Wang, Guangliang Zhang, Yuetao Wen

**Affiliations:** a Department of Physical Examination Center, Chongqing University Jiangjin Hospital, School of Medicine, Chongqing University, Chongqing, China; b Department of Neurosurgery, Yongchuan Hospital of Chongqing Medical University, Chongqing, China; c Department of Neurosurgery, Chongqing University Jiangjin Hospital, School of Medicine, Chongqing University, Chongqing, China

**Keywords:** Cuproptosis, Cuproptosis-related lncRNAs, Kidney renal clear cell carcinoma, Tumor immune

## Abstract

**Background:**

Kidney renal clear cell carcinoma (KIRC), with low survival rate, is the most frequent subtype of renal cell carcinoma. Recently, more and more studies indicate that cuproptosis-related genes (CRGs) and long non-coding RNAs (lncRNAs) play a vital role in the occurrence and development of many types of cancers. However, the roles of cuproptosis-related lncRNAs (CRlncRNAs) in the KIRC was uncertain.

**Results:**

In our study, CRlncRNAs were obtained by coexpression between differentially expressed and prognostic CRGs and differentially expressed and prognostic lncRNAs, and an 8-CRlncRNAs (AC007743.1, AC022915.1, AP005136.4, APCDD1L-DT, HAGLR, LINC02027, MANCR and SMARCA5-AS1) risk model was established according to least absolute shrinkage and selection operator (LASSO) and multivariate Cox regression. This risk model could differentiate immune cell infiltration, immune function and gene mutation.

**Conclusions:**

This 8-CRlncRNAs risk model may be promising for the clinical prediction of prognoses, tumor immune, immunotherapy response and chemotherapeutic response in KIRC patients.

## 1 Background

Renal cell carcinoma (RCC) is the most prevalent and malignant kidney cancer in adults, which shares 3.7 % of all malignancies in adults globally,^1^ kidney renal clear cell carcinoma (KIRC) accounting for about 80 %-90 % among all histological subtypes of RCC.^2^ It is knowable from the current studies of KIRC, a rising need is required to introduce predictive biomarkers and new forecasting to comsummate and reinforce the current testing system. More evidence suggests that the discovery of molecular biomarkers can provide a need for prognostic assessment and identification of potentially high-risk KIRC patients. What’s more, it might serve as therapeutic targets in the future. Recently, more attention has been payed to a novel cell death named cuproptosis, and it has been shown that copper binds directly to the fatty conponents of the tricarboxylic acid (TCA) cycle. It induces toxic protein stress and eventually induce cell death.^3^, ^4^ Studies also revealed that KIRC is generally accompanied by a reprogramming of the TCA cycle, In primary KIRC, the concentration of glycolysis intermediates was the highest, while that of TCA cycle intermediates was the lowest.^5^ The downregulating the energy production through a TCA cycle, which enabled tumor cells to survive in conditions of nutrient depletion and hypoxia and escape from the immune system.^6^, ^7^, ^8^

In 2022, Bian et al. systematically analyzed the landscape of molecular alternations and interactive genes of cuproptosis in KIRC. The study demonstrated that these cuproptosis-related genes (CRGs) may play a crucial role in KIRC outcomes. The prognostic risk score based on the expression signature of CRGs showed a good performance for the prediction of OS and PFS of KIRC patients and was significantly associated with immune infiltration levels and PD1 expression. These implied that genes involved in copper-induced cell death may offer novel strategies to predict the prognosis of KIRC patients.^9^

Long noncoding RNAs (lncRNAs) are a subset of RNA molecules with a length of 200 nucleotides that modelate gene expression.^10^ Besides gene regulation, lncRNA may exhibit tumor-suppressive and -promoting (oncogenic) functions. Own to their genome-wide expression patterns in a variety of tissues and their tissue-specific expression characteristics, lncRNAs bear strong promise as novel biomarkers and therapeutic targets for cancer.^11^ And studies revealed that lncRNAs are found to be upregulated in disorders related to pyroptosis, Tang et al. in 2021 revealed that pyroptosis-related lncRNAs signature can predict the prognosis of KIRC.^12^ Regarding the cuproptosis is also a new form of cell death, it may also occur in the KIRC, and now, there are just not many research on cuproptosis-related lncRNAs (CRlncRNAs). By getting data from The Cancer Genome Atlas (TCGA), we first generated predictive multilncRNA signatures of differentially expressed cell nucleopause related lncRNAs, and we suspected those could serve as the prognostic assessment and the identification of potentially high-risk KIRC patients.

## 2 Methods

### 2.1 Acquisition of datasets and preprocessing

The RNA transcriptome datasets, somatic mutation data and the relevant clinical information including futime, fustat, age, gender, grade, stage, T stage, N stage, and M stage of KIRC patients were downloaded from TCGA database (https://portal.gdc.cancer.gov/). There were 541 KIRC samples and 72 nontumorous samples in the RNA transcriptome datasets, 330 out of 541 KIRC patients had somatic mutation information and 532 out of 541 KIRC patients had clinical information. The FPKM value of RNA transcriptome datasets was convertedc to the TPM value, and the expression data of protein-coding genes and lncRNAs were acquired using the Ensembl human genome browser (https://asia.ensembl.org/info/data/index.html) and Strawberry Perl program.

### 2.2 Selection of CRGs and CRlncRNAs

A total of 19 CRGs (NFE2L2, NLRP3, ATP7B, ATP7A, SLC31A1, FDX1, LIAS, LIPT1, LIPT2, DLD, DLAT, PDHA1, PDHB, MTF1, GLS, CDKN2A, DBT, GCSH and DLST) were summarized from previously published report.^4^ The differential expression analysis of CRGs and lncRNAs between tumor samples and nontumorous samples were performed by using the R package “limma” with a FDR < 0.05. Moreover, the prognostic CRGs and lncRNAs were obtained by using univariate Cox (uni-Cox) regression analysis with a *p* value < 0.05. In this study, we found 13 differentially expressed and prognostic CRGs and 2076 differentially expressed and prognostic lncRNAs. Then spearman correlation analysis was performed to analyze the correlation between 2076 lncRNAs and 13 CRGs, and CRlncRNAs was defined as lncRNAs significantly related to at least one CRGs (|spearman correlation| ≥ 0.4 and *p* value < 0.001). Finally, we got 312 CRlncRNAs for further study.

### 2.3 Construction of CRlncRNAs risk model

312 CRlncRNAs were incorporated into the least absolute shrinkage and selection operator (LASSO) by using the R package “glmnet” with 10-fold cross-validation and a *p* value < 0.05,^13^ and we found 20 CRlncRNAs out of 312 CRlncRNAs were distinctly related to the OS of KIRC patient. Then, multivariate Cox (multi-Cox) was used to analyze 20 CRlncRNAs, and an 8-CRlncRNAs risk model was ultimately established.^14^ The risk score was calculated according to the following formula:

$$Risk\;score=\sum_{k=0}^{n}Coefficient\left({\mathrm{lncRNA}}_{k}\right)*Expression\left({\mathrm{lncRNA}}_{k}\right)$$

Using the median risk score as the cut-off value, KIRC patients in training set and testing set were divided into low- or high-risk groups. Kaplan-Meier curve with log-rank test was used to compare OS between the high- and low-risk groups by using the R package “survival” and “survminer”.

### 2.4 Validation of the predictive effect of CRlncRNAs risk model

To test whether the clinical features such as age, gender, tumor grade, tumor stage, T stage, M stage and N stage could affect the predicted effect of our model, KIRC patients in entire set were stratified by age (<60 years and ≥60 years), gender (male and female), tumor grade (G1–G2 and G3–G4), tumor stage (stage I–II and stage III–IV), T stage (T1–T2 and T3–T4), M stage (M0 and M1) and N stage (N0–N1 and NX), and Kaplan-Meier curve with log-rank test was used to compare OS between the high- and low-risk groups in each stratify by using the R package “survival” and “survminer”.

The uni-Cox regression and multi-Cox regression analyses were utilized to evaluate whether the CRlncRNAs risk score and clinical characteristics, including age, gender, grade and stage were independent variable factors to predict prognosis by using the R package “survminer”. The concordance index (C-index) was used to evaluate the predictive effect of CRlncRNAs risk scores on the prognostic of KIRC patients in the TCGA cohort by using the R package “survival”, “rms” and “pec”. Using the R package “survival”, “survminer” and “timeROC”, the 1-year survival, 3-year survival and 5-year survival receiver operating characteristic (ROC) curves were computed to analyze the specificity and sensitivity of the CRlncRNAs risk model according to the area under the curve (AUC) value of ROC curve. Moreover, to illustrate whether the prediction outcome showed good consistence with the practical, four independent prognostic factors, including age, grade, stage and risk score (all *p* < 0.05 in uni-Cox and multi-Cox) were used to set up a nomogram for the 1-, 3-, and 5-year OS and correction curves by using the Hosmer-Lemeshow test with the R package “survival”, “regplot” and “rms”.

### 2.5 Principal-component analysis and concordance index

Principal-component analysis (PCA) was used for effective dimensionality reduction, model identification, and grouping visualization of high-dimensional data of the entire gene expression profiles, 13 CRGs, 8 CRlncRNAs, and risk model according to the expression patterns of the 8 CRlncRNAs.^14^ The concordance index (C-index) was used to estimate the predictive accuracy and discrimination ability of each factor.

### 2.6 Functional enrichment analysis and gene set enrichment analyses

The differentially expressed genes between the high-risk and low-risk groups in the entire set between the high- and low-risk groups were identified by using the R package “limma” with |log2FC| > 1 and a *p* value < 0.05), and the Gene Ontology (GO) and the Kyoto Encyclopedia of genes and Genomes (KEGG) pathway analyses of those differentially expressed genes were performed by using R package “clusterProfiler” “org.Hs.eg.db” and “enrichplot”. Gene Set Enrichment Analysis (GSEA) was employed to identify the biological functions and pathways associated with the high and low-risk groups by using using R package “clusterProfiler” “org.Hs.eg.db” and “enrichplot”.

### 2.7 Evaluation of tumor microenvironment

The immune score and stromal score of each KIRC sample, which are positively related to the ratio of immune and stromal cells, were calculated according to the expression of immune and stromal cells, and the tumor microenvironment scores (immune score, stromal score and ESTIMATE score) were obtained using ESTIMATE algorithm.^15^ Firstly, the samples were divided into the low and high groups by the median of the the tumor microenvironment scores, and Kaplan-Meier method was used to determine the relationship between the tumor microenvironment scores and OS of KIRC patients. Then, we compared the immune score, stromal score and ESTIMATE score between low- and high-risk groups. Lastly, spearman correlation coefficient was applied to evaluate the correlation between risk score and tumor microenvironment scores.

### 2.8 Evaluation of immune cells infiltration

To identify immune cell infiltration of KIRC samples, their expression data were imported into CIBERSORT and iterated 1000 times to estimate the relative proportion of 22 types of immune cells, and samples with a CIBERSORT output of p-value less than 0.05 was used for further analyses.^16^ Firstly, the samples were divided into the low and high groups by the median of the level of immune cells, and Kaplan-Meier method was used to determine the relationship between the 22 types of immune cells and OS of KIRC patients. Then, we analyzed the difference in 22 types of immune cells between the low and high-risk groups in those samples. Lastly, spearman correlation coefficient was applied to evaluate the correlation between risk score and immune cell levels.

### 2.9 Evaluation of immune function

To identify immune function of KIRC samples, we got the immune function scores by using R package “limma”, “GSVA” “GSEABase” and “reshape2”.^17^, ^18^ Firstly, the samples were divided into the low and high groups by the median of immune function score, and Kaplan-Meier method was used to determine the relationship between the immune function and OS of KIRC patients. Then, we analysed the difference in the immune function between the low and high-risk groups in KIRC samples. Lastly, spearman correlation coefficient was applied to evaluate the correlation between risk score and immune function scores.

### 2.10 Evaluation of tumor mutation burden

The tumor mutation burden (TMB) was calculated as mutations per megabase (mut/Mb). Mutation annotation format (MAF) was used to store the somatic variant data. We then qualitatively and quantitatively analyzed the somatic variant data among different subgroups by using the R package “maftools”. The samples were divided into the low and high groups by the median of TBM, and Kaplan-Meier method was used to determine the relationship between the TBM and OS of KIRC patients. Also, we combined the risk score and TMB to analyze the impact on OS of KIRC patients. Then, we analyzed the difference in the TMB between the low and high-risk groups in KIRC samples. Lastly, spearman correlation coefficient was applied to evaluate the correlation between risk score and TBM.

### 2.11 Evaluation of immunotherapy response and chemotherapeutic response

We used tumor immune dysfunction and exclusion (TIDE; https://tide.dfci.harvard.edu) algorithm to evaluate the immunotherapy response of each patient.^19^ The R package of “pRRophetic” was used to predict half-maximal inhibitory concentration (IC50) of common chemotherapeutic agents according to tumor gene expression level.^20^

### 2.12 Statistical analysis

Strawberry (version perl 5.30.0) and R software (version 4.1.3) were applied for processing data, statistical analyses and graph visualizations. Chi-square or fisher test was used to analyze categorical variables, the LASSO regression was used to calculate coefficients of the prognostic signature, the Kaplan–Meier method and a log-rank test were used to compare survival rates between groups, the spearman test was used for the correlation analysis, uni-Cox regression and multi-Cox regression were conducted to examine the independent risk factors for the model, and the Wilcoxon test was performed to compare the differences between groups of continuous data. Unless specifically mentioned, a *p* < 0.05 is considered statistically significant.

## 3 Results

### 3.1 Identification of CRlncRNAs

**Fig. 1:**
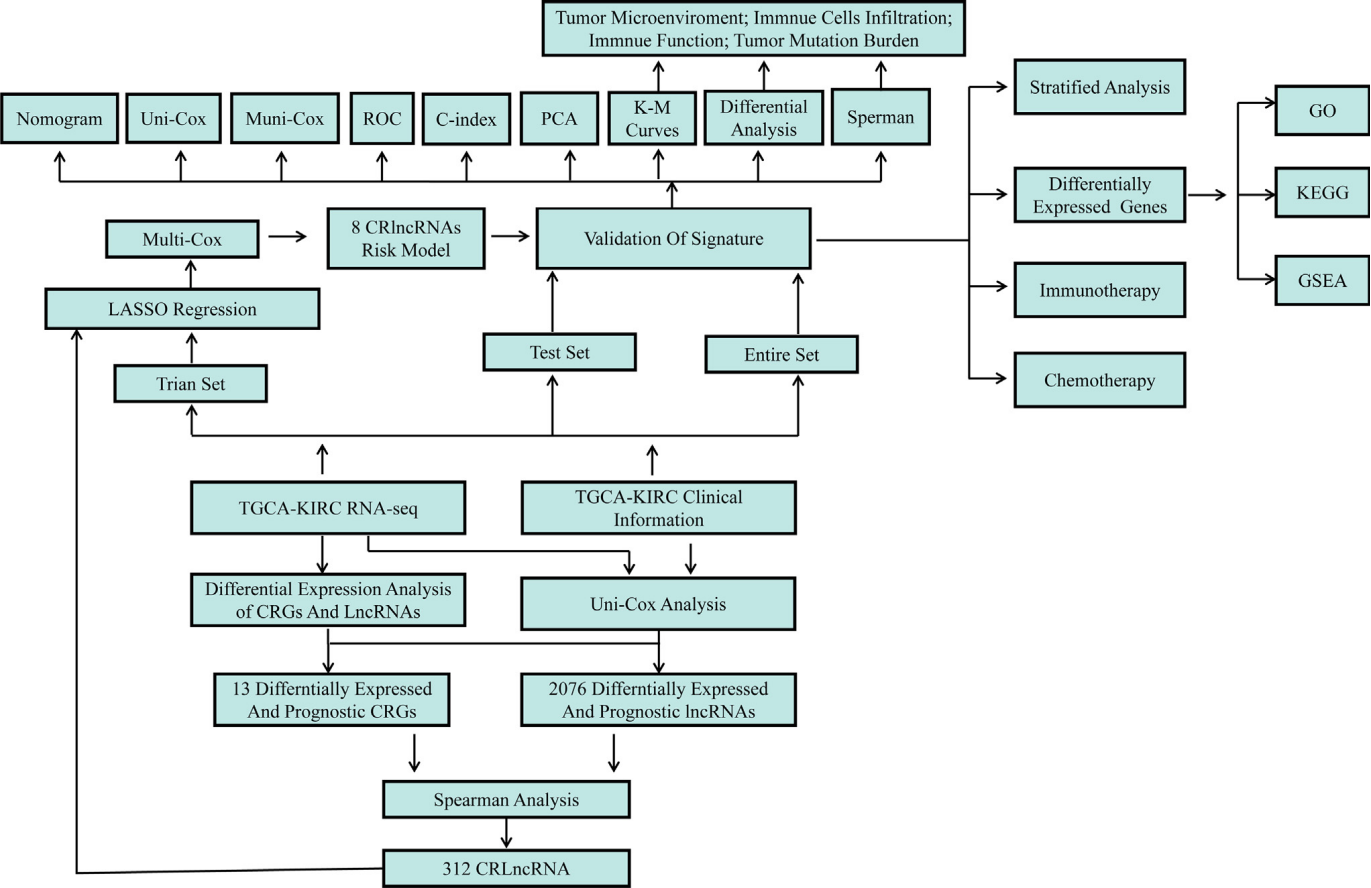
**The workflow chart of this study**.

**Fig. 2:**
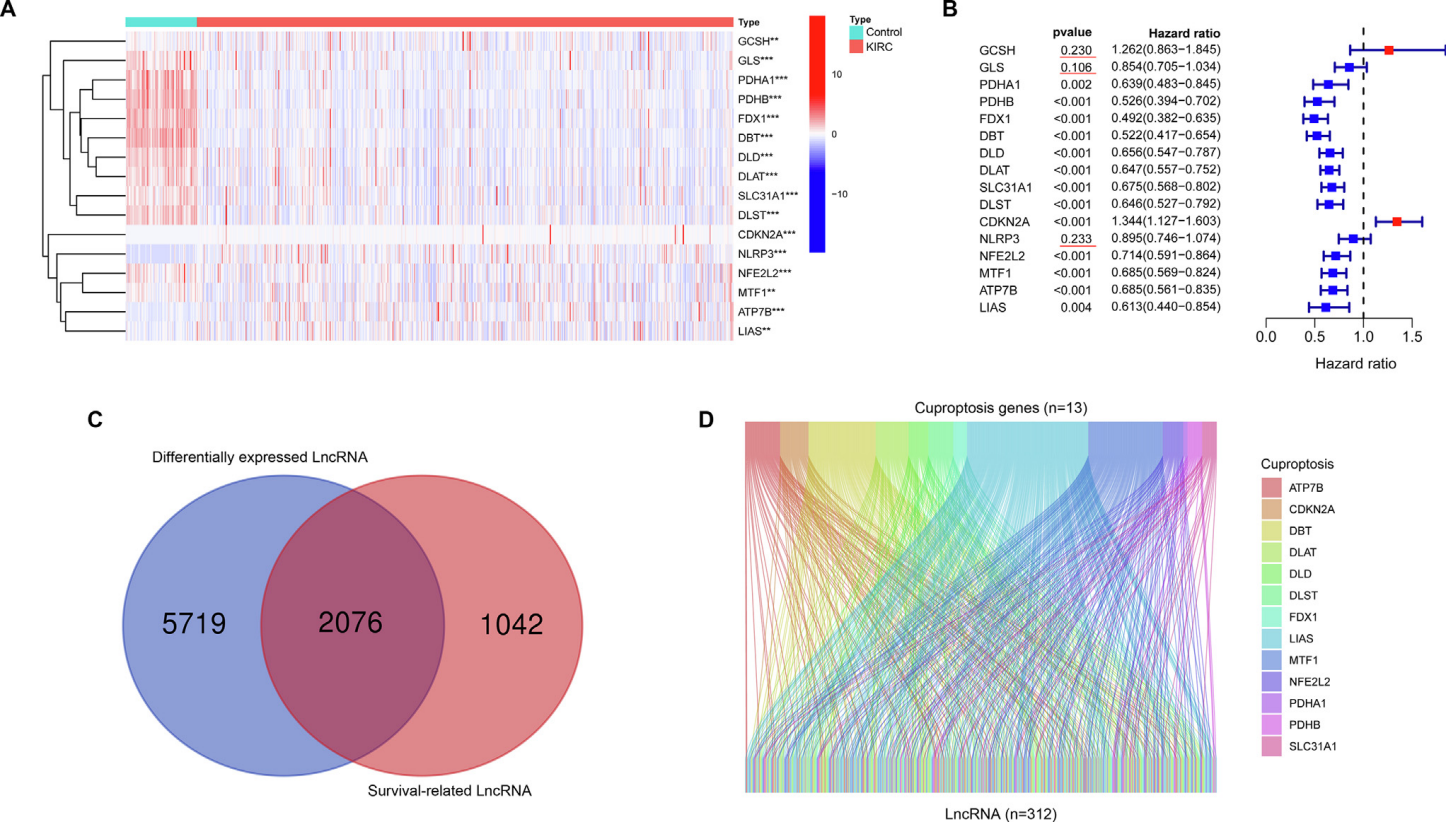
**Identification of CRlncRNAs. (A)** Differential expression analysis of CRGs in the TCGA-KIRC. **(B)** Uni-Cox regression analysis revealed that the CRGs significantly correlated with clinical prognosis. **(C)** Venn diagram to identify the common lncRNAs of differentially expressed CRGs and prognosis-related CRGs. **(D)** Sankey diagram for 13 CRGs and 321 CRlncRNAs.

The detailed flow chart for risk model construction and subsequent analyses is shown in Fig. 1. We extracted the expression information of mRNAs and lncRNAs from TCGA-KIRC RNA transcriptome datasets, and we found that 16 CRGs (Fig. 2A) and 7795 lncRNAs (Supplementary Table 1) were differentially expressed in the KIRC samples when compared with controls through Wilcoxon test analysis (FDR < 0.05). After conducting uni-Cox regression analysis, we identified 13 CRGs (Fig. 2B) and 3118 lncRNAs (Supplementary Table 2) which were highly correlated with overall survival (*p* < 0.05). According to expression and uni-Cox regression analysis, we got 13 CRGs (PDHA, PDHB, FDX1, DBT, DLD, DLAT, SLC31A1, DLST, CDKN2A, NFE2L2, MTF1, ATP7B and LIAS) and 2076 lncRNAs (Fig. 2C). Finally, the spearman correlation analysis was performed to analyze the correlation between 2076 lncRNAs and 13 CRGs, and CRlncRNAs was defined as lncRNAs significantly related to at least one CRGs (|spearman correlation| ≥ 0.4 and *p* value < 0.001). The coexpression network was visualized using the Sankey diagram, and we got 312 CRlncRNAs (Fig. 2D).

### 3.2 Construction and validation of an 8-CRlncRNAs risk model

**Fig. 3:**
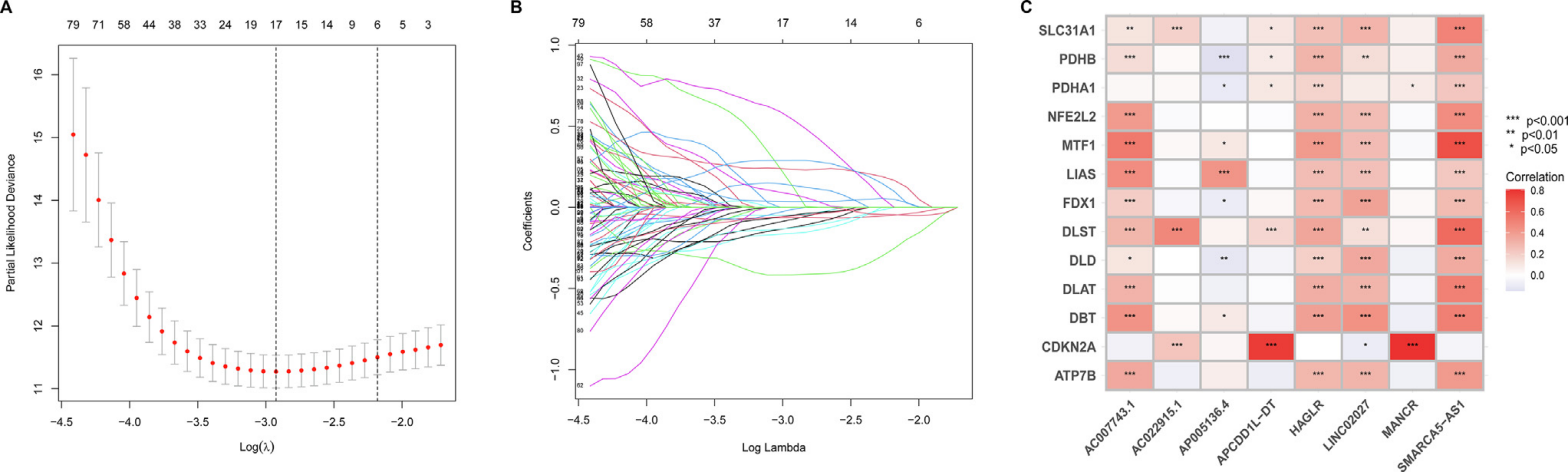
**Construction of an 8-CRlncRNAs risk model. (A)** Selecting the best tuning parameters (log λ) of OS-related for KIRC by using LASSO regression analysis. **(B)** LASSO coefficient spectrum of 17 CRlncRNA. **(C)** Heatmap for the correlations between 13 CRGs and 8 CRlncRNAs.

532 KIRC patients of the entire TCGA set were randomly divided into training set (n = 266) and testing set (n = 266). The train set was used to construct an CRlncRNAs risk model, and the entire set and testing set were used to validate this model. The clinical characteristics of the train set and test set were shown in Table 1, and statistical analysis indicated that there were no statistical differences in age, gender, grade, stage and TNM grade between the train set and test set (*p* < 0.05). LASSO regression analysis is often used to construct a prognostic model in high-dimensional data with an inferior correlation and prominent forecasted value to avoid overfitting. In the train set, 312 CRlncRNAs were subjected to LASSO regression, and we found 17 CRlncRNAs were distinctly related to the OS of KIRC patient (Fig. 3A and B). Then, we found that 8 CRlncRNAs (AC007743.1, AC022915.1, AP005136.4, APCDD1L-DT, HAGLR, LINC02027, MANCR and SMARCA5-AS1) were independently correlated with OS by using multi-Cox analysis, and those CRlncRNAs were used to calculate the risk score of each KIRC patient. The correlation between 13 CRGs and 8 CRlncRNAs in the the entire set was shown in Fig. 3C.

**Table 1:** The clinical characteristics of the train set and test set.

Features	Total	Test	Train	*p* value
Age				1
<60	245 46.05 %)	123 (46.24 %)	122 (45.86 %)	
>=60	287 (53.95 %)	143 (53.76 %)	144 (54.14 %)	
Gender				0.7164
Female	187 (35.15 %)	91 (34.21 %)	96 (36.09 %)	
Male	345 (64.85 %)	175 (65.79 %)	170 (63.91 %)	
Grade				0.8182
G1	14 (2.63 %)	6 (2.26 %)	8 (3.01 %)	
G2	228 (42.86 %)	110 (41.35 %)	118 (44.36 %)	
G3	206 (38.72 %)	107 (40.23 %)	99 (37.22 %)	
G4	76 (14.29 %)	39 (14.66 %)	37 (13.91 %)	
Unknown	8 (1.5 %)	4 (1.5 %)	4 (1.5 %)	
Stage				0.8738
Stage I	266 (50 %)	137 (51.5 %)	129 (48.5 %)	
Stage II	57 (10.71 %)	26 (9.77 %)	31 (11.65 %)	
Stage III	123 (23.12 %)	61 (22.93 %)	62 (23.31 %)	
Stage IV	83 (15.6 %)	41 (15.41 %)	42 (15.79 %)	
Unknown	3 (0.56 %)	1 (0.38 %)	2 (0.75 %)	
T				0.778
T1	272 (51.13 %)	138 (51.88 %)	134 (50.38 %)	
T2	69 (12.97 %)	33 (12.41 %)	36 (13.53 %)	
T3	180 (33.83 %)	88 (33.08 %)	92 (34.59 %)	
T4	11 (2.07 %)	7 (2.63 %)	4 (1.5 %)	
M				0.689
M0	421 (79.14 %)	216 (81.2 %)	205 (77.07 %)	
M1	79 (14.85 %)	38 (14.29 %)	41 (15.41 %)	
Unknown	32 (6.02 %)	12 (4.51 %)	20 (7.52 %)	
N				0.8464
N0	240 (45.11 %)	121 (45.49 %)	119 (44.74 %)	
N1	16 (3.01 %)	9 (3.38 %)	7 (2.63 %)	
Unknown	276 (51.88 %)	136 (51.13 %)	140 (52.63 %)

**Fig. 4:**
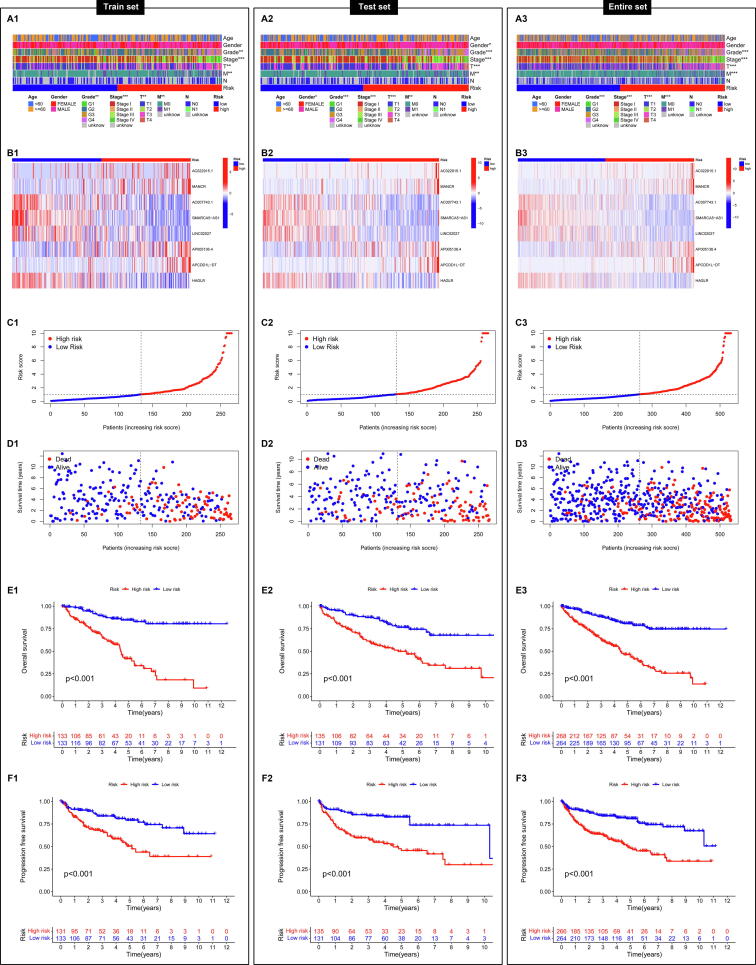
**The clinical characteristics, expression of 8 CRlncRNAs, risk score, survival time, OS and PFS in the two groups of the train set, test set and entire set. (A)** The clinical characteristics in the two groups of the train set (A1), test set (A2) and entire set (A3). **(B)** The expression of 8 CRlncRNAs in the two groups of the train set (B1), test set (B2) and entire set (B3). **(C)** The risk score in the two groups of the train set (C1), test set (C2) and entire set (C3). **(D)** The survival time in the two groups of the train set (D1), test set (D2) and entire set (D3). **(E)** The OS in the two groups of the train set (E1), test set (E2) and entire set (E3). **(F)** The PFS in the two groups of the train set (F1), test set (F2) and entire set (F3).

**Fig. 5:**
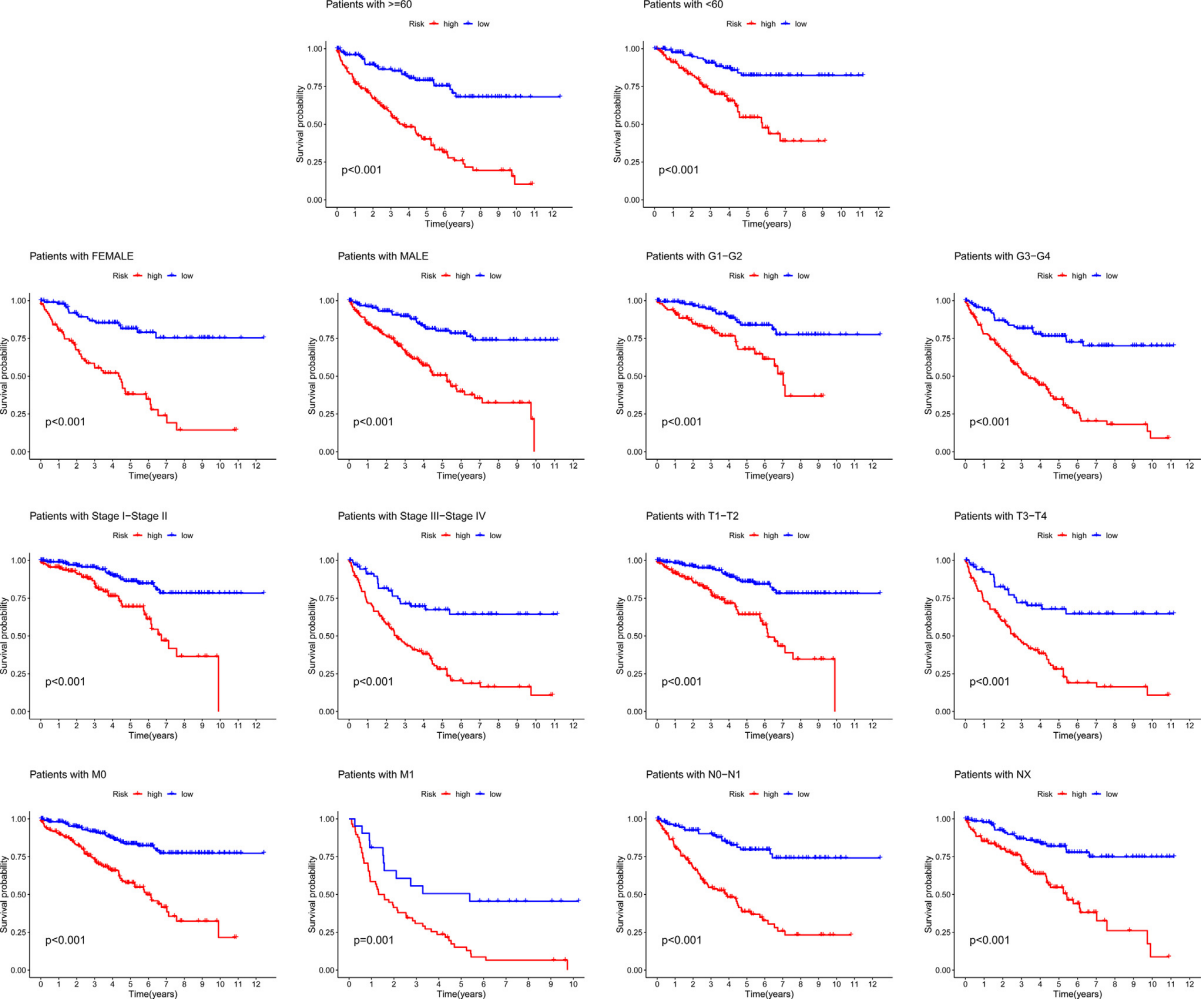
**Survival curves stratified by age, gender, grade, stage, T, N, or M between the high-risk group and low-risk group in the entire set**.

According to the level of risk score, KIRC patients could be divided into high-risk group and low-risk group in the train set, test set and entire set. The analyses of clinical characteristics indicated that there were statistical differences in grade, stage, T grade and M grade between the high-risk group and low-risk group in the train set (Fig. 4A1), test set (Fig. 4A2) and entire set (Fig. 4A3). The expression of 8 CRlncRNAs (Fig. 4B), risk score (Fig. 4C), survival time (Fig. 4D), OS (Fig. 4E) and PFS (Fig. 4F) in the two groups were compared in the train set, test set and entire set. The results indicated that patients in the high-risk group had higher score and worse prognosis than in the low-risk group. Moreover, we analyzed the OS between the high-risk group and low-risk group in the entire set according to the subgroups classified by age, gender, grade, stage, or TMN grade, and found that patients in the high-risk group had worse prognosis than in the low-risk group (Fig. 5). Those data indicated that this 8-CRlncRNAs risk model could be a good model to predict the prognosis of KIRC patient.

**Fig. 6:**
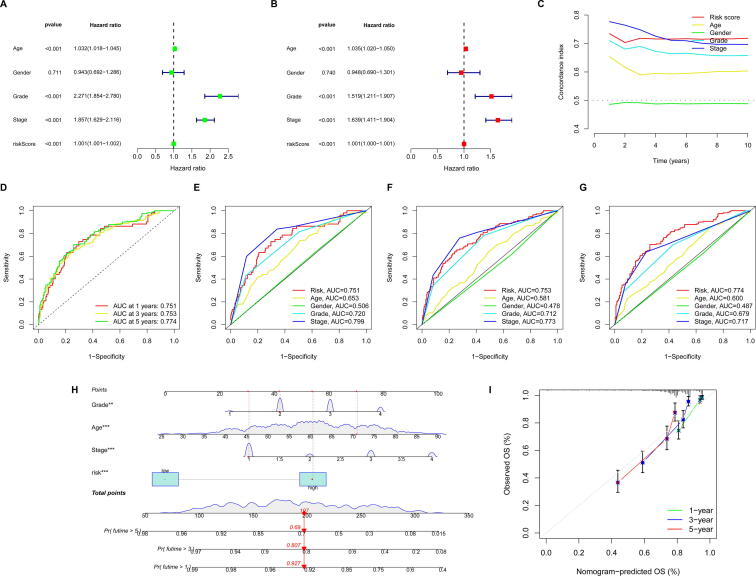
**Assessment of the risk model and construction of nomogram. (A)** Uni-Cox regression analysis of risk score and clinical factors with OS. **(B)** Multi-Cox regression analysis of risk score and clinical factors with OS. **(C)** C-index analysis of risk score and clinical factors with OS. **(D)** The ROC curves for 1-, 3-, and 5-years OS rate of risk score in the entire set. **(E-G)** The ROC curves for 1-, 3-, and 5-years OS rate of risk score and clinical factors in the entire set. **(H)** The nomogram that integrated the age, grade, stage and risk score to predict the 1-, 3-, and 5-years OS rate. **(I)** The calibration curves for 1-, 3-, and 5-years OS.

The uni-Cox regression analysis indicated that age (HR = 1.032, *p* < 0.001), grade (HR = 2.271, *p* < 0.001), stage (HR = 1.857, *p* < 0.001) and risk score (HR = 1.001, *p* < 0.001) were significantly associated with OS (Fig. 6A). The multi-Cox regression analysis indicated that age (HR = 1.035, *p* < 0.001), grade (HR = 1.519, *p* < 0.001), stage (HR = 1.639, *p* < 0.001) and risk score (HR = 1.001, *p* < 0.001) were significantly associated with OS (Fig. 6B). C-index analysis indicated that CRlncRNAs risk scores had the good predictive effect on the prognosis of KIRC patients (Fig. 6C). ROC curves were utilized to evaluate the accuracy of the risk model, and the results showed that the 1-, 3-, and 5-years area under the ROC curve of the entire set were 0.751, 0.753, and 0.774, respectively (Fig. 6D). At the 1-year ROC and the 3-year ROC, the predictive ability of risk score was only worse than stage, while at the 5-year ROC, risk score had a better predictive ability compared with other clinical factors (Fig. 6E–G). Moreover, to illustrate whether the prediction outcome showed good consistence with the practical, four independent prognostic factors, including age, grade, stage and risk score (all *p* < 0.05 in uni-Cox and multi-Cox) were used to set up a nomogram for predicting the 1, 3, 5-years OS rate (Fig. 6H), and the calibration plots indicated that the nomogram was well concordant with the prediction for 1, 3, and 5 years (Fig. 6I).

**Fig. 7:**
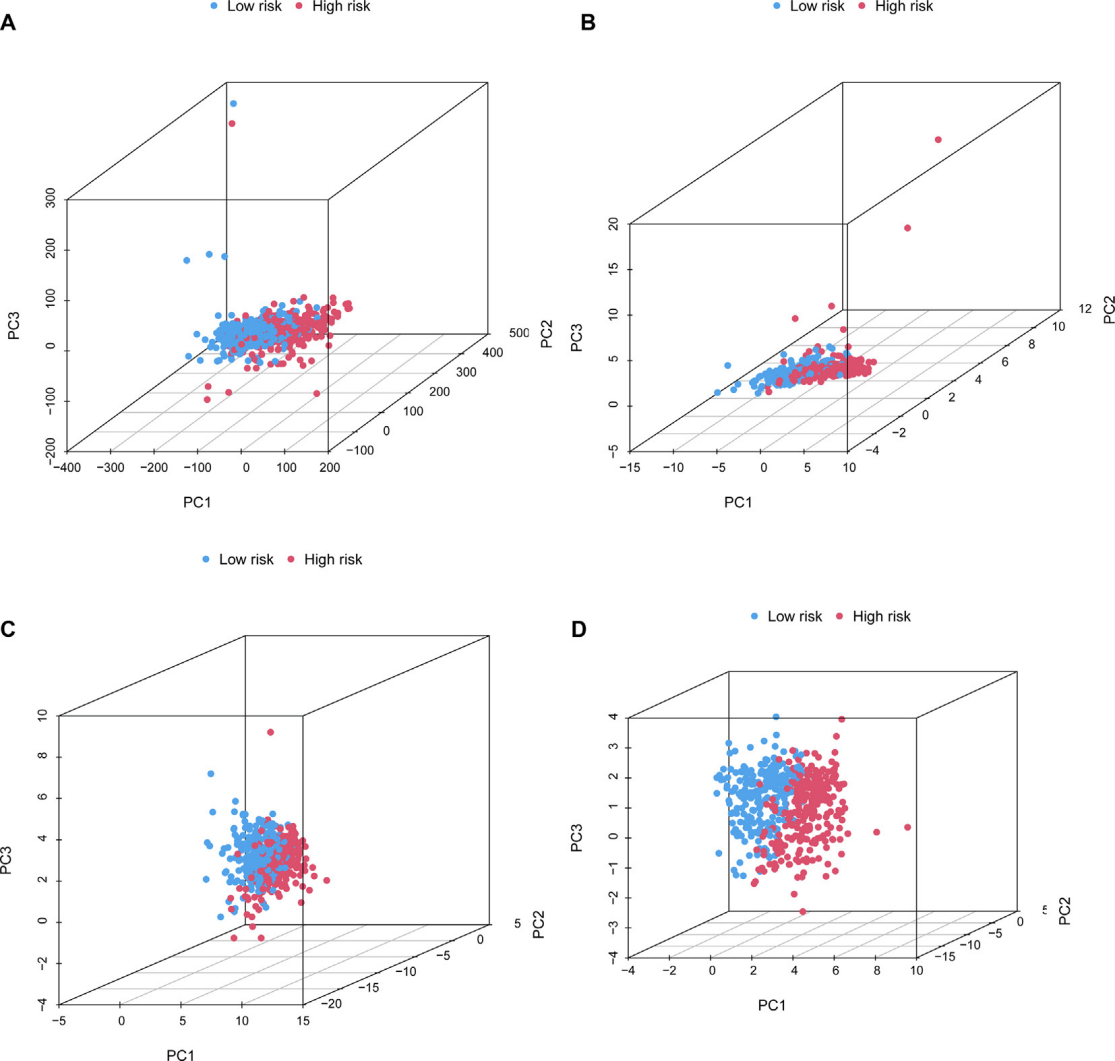
**Principal component analysis. (A)** PCA between the high- and low-risk groups based on all gene expression profiles. **(B)** PCA between the high- and low-risk groups based on 13 CRGs. **(C)** PCA between the high- and low-risk groups based on 8 CRlncRNAs. **(D)** PCA between the high- and low-risk groups based on risk model.

Finally, PCA was conducted to test the difference between the low-risk and high-risk groups based on all gene expression profiles (Fig. 7A), 13 CRGs (Fig. 7B), 9 CRlncRNAs (Fig. 7C) and risk model Figure (Fig. 7D). PCA results indicated that this risk model could distinguish the prognosis of KIRC patients.

### 3.3 Functional enrichment analysis

**Fig. 8:**
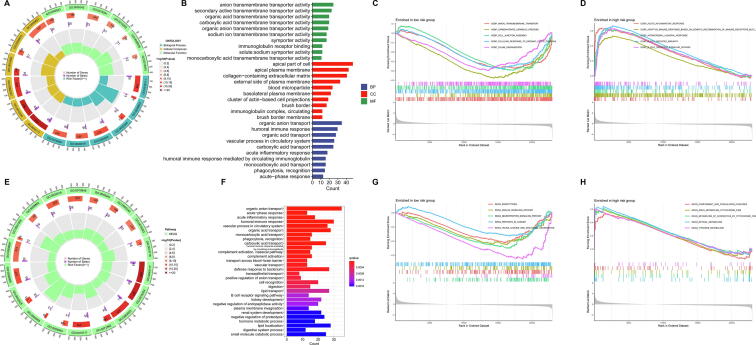
**Functional enrichment for differentially expressed genes between the low-risk group and high-risk group. (A)** The circle diagram enriched in the GO analysis. **(B)** The top 30 significant terms of GO functional enrichment. **(C)** The top 5 GO pathway in the low-risk group. **(D)** The top 5 GO pathway in the high-risk group. **(E)** The circle diagram enriched in the KEGG analysis. **(F)** The top 30 significant terms of KEGG functional enrichment. **(G)** The top 5 KEGG pathway in the low-risk group. **(H)** The top 5 KEGG pathway in the high-risk group.

According to the level of risk score, 532 KIRC patients could be divided into high-risk group and low-risk group. We found 711 differentially expressed genes between high-risk group and low-risk group with the criteria of |log2 FC| > 1 and *p* < 0.05 (Supplementary Table 3), and functional enrichment analysis of those genes were performed. The GO terms revealed that these genes were significantly associated with anion transmembrane transporter activity, apical part of cell and organic anion transport in the biological processes (BP), cellular components (CC) and molecular functions (MF), respectively (Fig. 8A, B). Functional annotation of GO terms was further performed using GSEA, and the top 5 pathway significantly enriched in the low-risk group and high-risk group were shown in the Fig. 8C and D, respectively. The KEGG terms revealed that these genes were significantly associated with organic anion transport (Fig. 8E, F). Functional annotation of KEGG terms was further performed using GSEA, and the top 5 pathway significantly enriched in the low-risk group and high-risk group were shown in the Fig. 8G and H, respectively.

### 3.4 Relationship between the immune and risk model

**Fig. 9:**
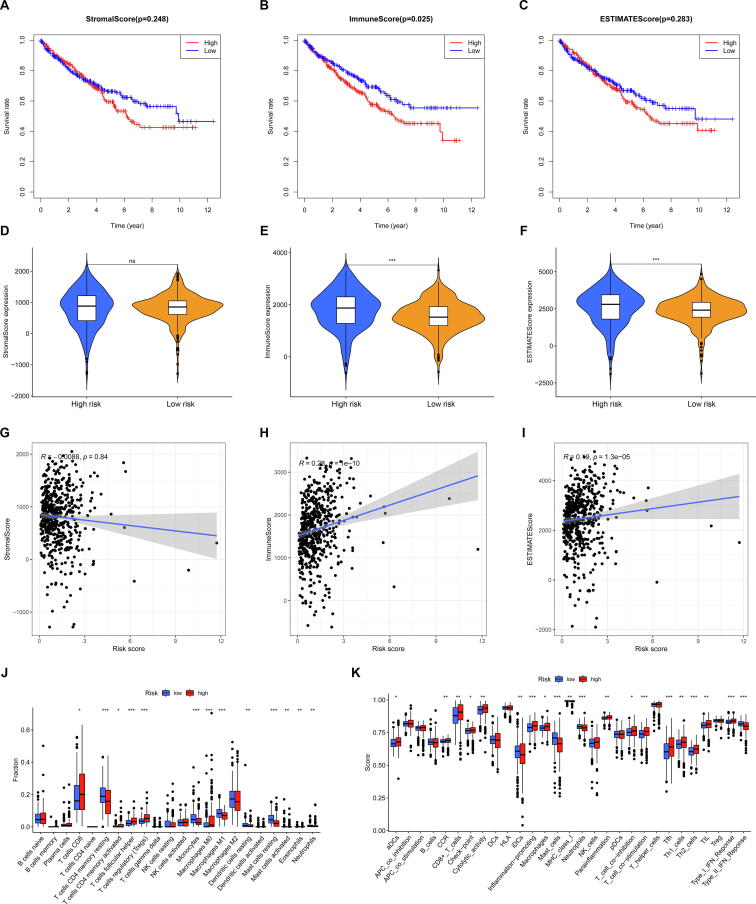
**Relationship between the immune and risk model.** Kaplan–Meier analysis to explore the effects of stromal score **(A)**, immune score **(B)** and ESTIMATE score **(C)** on the prognosis of KIRC patients. Comparison of the stromal score **(D)**, immune score **(E)** and ESTIMATE score **(F)** in the high- and low-risk groups. Spearman correlation analysis between the risk score and the stromal score **(G)**, immune score **(H)** and ESTIMATE score **(I)**. Comparison of the immune cell infiltration **(E)** and immune function **(K)** in the high- and low-risk groups.

The ESTIMATE algorithm was used to calculate the microenvironment scores of KIRC (including stromal, immune and ESTIMATE scores). We divided KIRC patients into high- and low groups according to the median value of three kinds of scores and conducted Kaplan–Meier analysis to explore their effects on the prognosis of KIRC. We found that stromal score and ESTIMATE score could not predict prognosis, and KIRC patients with lower immune score had a better prognosis (Fig. 9A–C). According to comparing the differences of the three kinds of tumor microenvironment scores between low-risk group and high-risk group, we found that there was no statistic difference in the stromal score between high-risk and low-risk groups (Fig. 9D), but immune score (Fig. 9E) and ESTIMATE score (Fig. 9F) were obviously lower in the low-risk group than in the high-risk group. Moreover, according to spearman correlation test, we found that the obviously positive association of risk score with immune score and ESTIMATE score (Fig. 9G–I).

**Supplementary Fig. 1:**
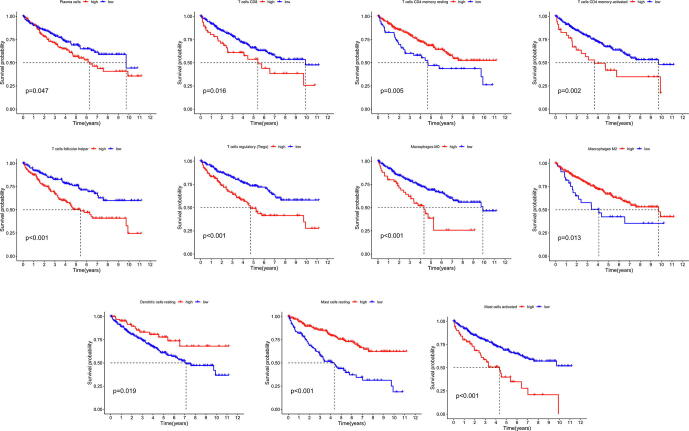
Many immune cells were associated with prognosis of KIRC patients.

**Supplementary Fig. 2:**
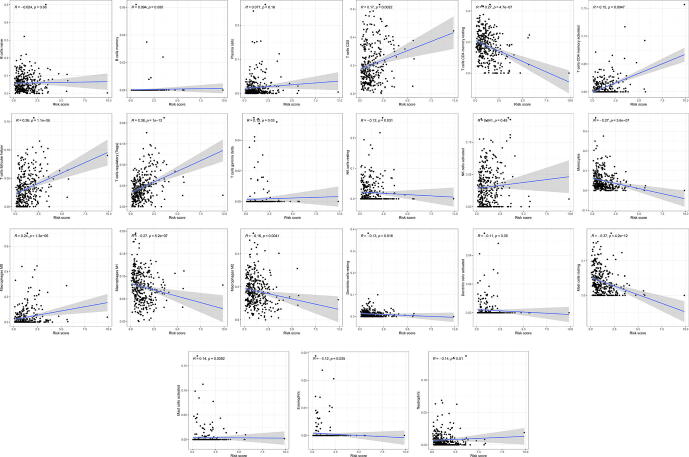
Spearman correlation analysis between the risk score and the 22 types of immune cells.

**Supplementary Fig. 3:**
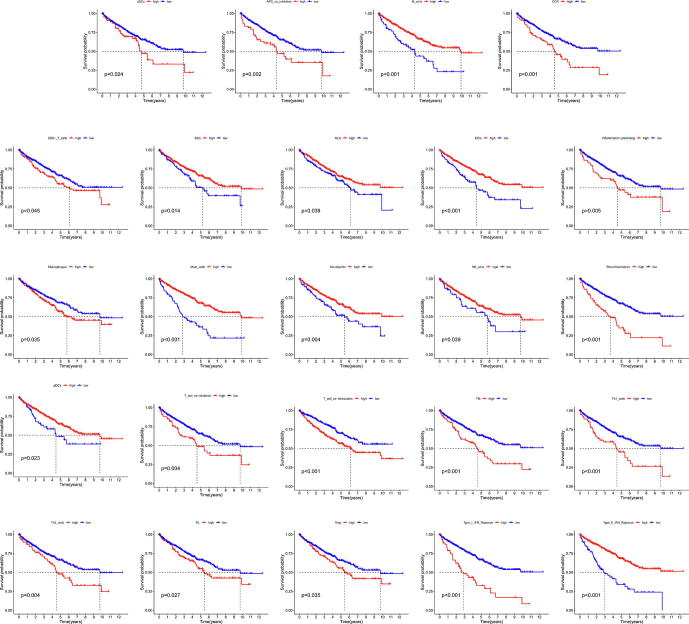
Many immune functions were associated with prognosis of KIRC patients.

**Supplementary Fig. 4:**
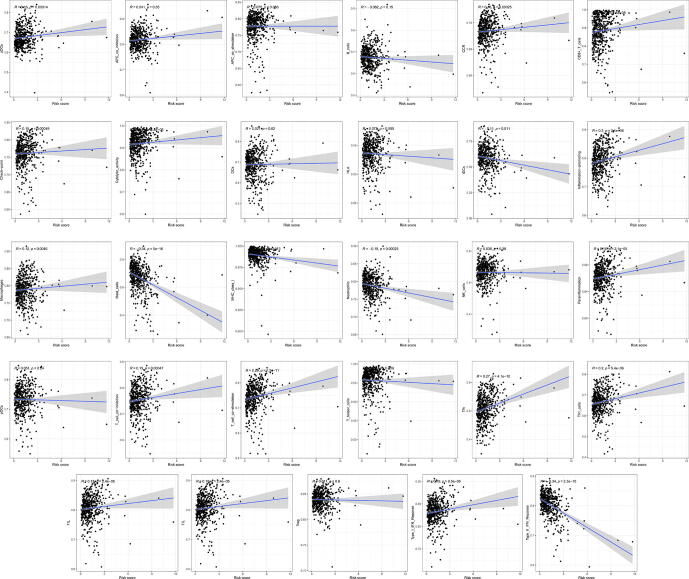
Spearman correlation analysis between the risk score and the immune function score.

The above data of tumor microenvironment analysis indicated that immune was related with prognosis of KIRC and the risk model we constructed could be use to assess immune. Therefore, we performed the analysis of immune cell infiltration and immune function. For immune cell infiltration, we divided KIRC patients into high- and low groups according to the median value of 22 types of immune cells, and found that many types of immune cells, such as plasma cells, T cell CD8, macrophages M0, T cells follicular helper, etc., were associated with prognosis of KIRC patients (Supplementary Fig. 1). According to comparing the differences of 22 types of immune cells between low-risk group and high-risk group, we found that many types of immune cells, such as T cell CD8, T cells CD4 memory resting, T cells follicular helper, etc., were differential infiltration in the low-risk group and high-risk group (Fig. 9J). According to spearman correlation test, we found that many types of immune cells, such as, T cell CD8, T cells CD4 memory resting, T cells follicular helper, etc., were associated with risk score (Supplementary Fig. 2). For immune function, we divided KIRC patients into high- and low groups according to the median value of immune function score, and found that many immune function, such as aDCs, APC_co_inhibition, inflammation-promoting, type_I_IFN_Repons, etc., were associated with prognosis of KIRC patients (Supplementary Fig. 3). According to comparing the differences of immune function score between low-risk group and high-risk group, we found that many types of immune function, such as CD8+_T_cells, iDCs, type_I_IFN_Repons, etc., in the low-risk group were differential when compared with high-risk group (Fig. 9K). According to spearman correlation test, we found that many types of immune function, such as, CCR, iDCs, inflammation-promoting, etc., were associated with risk score (Supplementary Fig. 4).

### 3.5 Relationship between the gene mutation and risk model

**Fig. 10:**
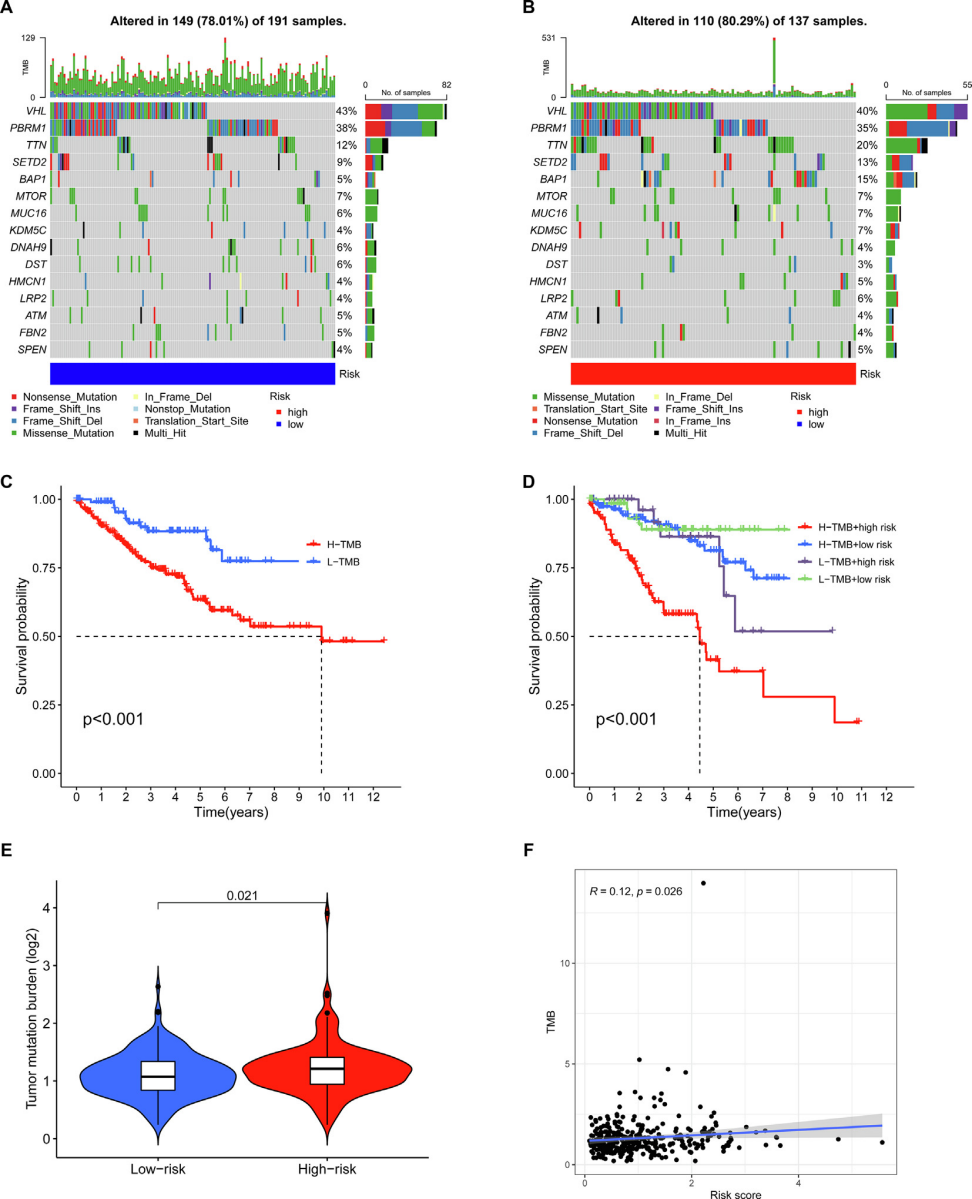
**Relationship between the gene mutation and risk model. (A)** Top-15 gene mutation frequency in low risk group. **(B)** Top-15 gene mutation frequency in high risk group. **(C)** Survival curves of the high-TMB group and low-TMB group. **(D)** Survival curves of the combined the risk score and TMB. **(E)** Comparison of the TMB in the high- and low-risk groups. **(F)** Spearman correlation analysis between the risk score and the TMB.

We analyzed tumor mutation burden and gene mutation frequency in the low-risk group and high-risk group. As shown in the waterfall plots which were used to visualize the mutation frequency and mutation type of the top 15 genes, VHL had the highest gene mutation frequency in the low-risk group (Fig. 10A) and high-risk group (Fig. 10B), and the main mutation type of VHL in the low-risk group was missense mutation and frameshift deletion mutation (Fig. 10A), and the main mutation type of VHL in the high-risk group was missense mutation (Fig. 10B). Kaplan–Meier analysis indicated that patients with low TMB had better prognosis than patients with high TMB (Fig. 10C). Moreover, we combined the risk score and TMB to analyze the impact on OS, and found that patients with low TMB and low risk score had the best prognosis, while patients with high TMB and high risk score had the worst prognosis (Fig. 10D). According to comparing the differences of the TMB between low-risk group and high-risk group, we found that TMB was obviously lower in the low-risk group than in the high-risk group (Fig. 10E). Moreover, according to spearman correlation test, we found that the TBM was positively associated with risk score (Fig. 10F).

### 3.6 Immunotherapy response and chemotherapeutic response

**Fig. 11:**
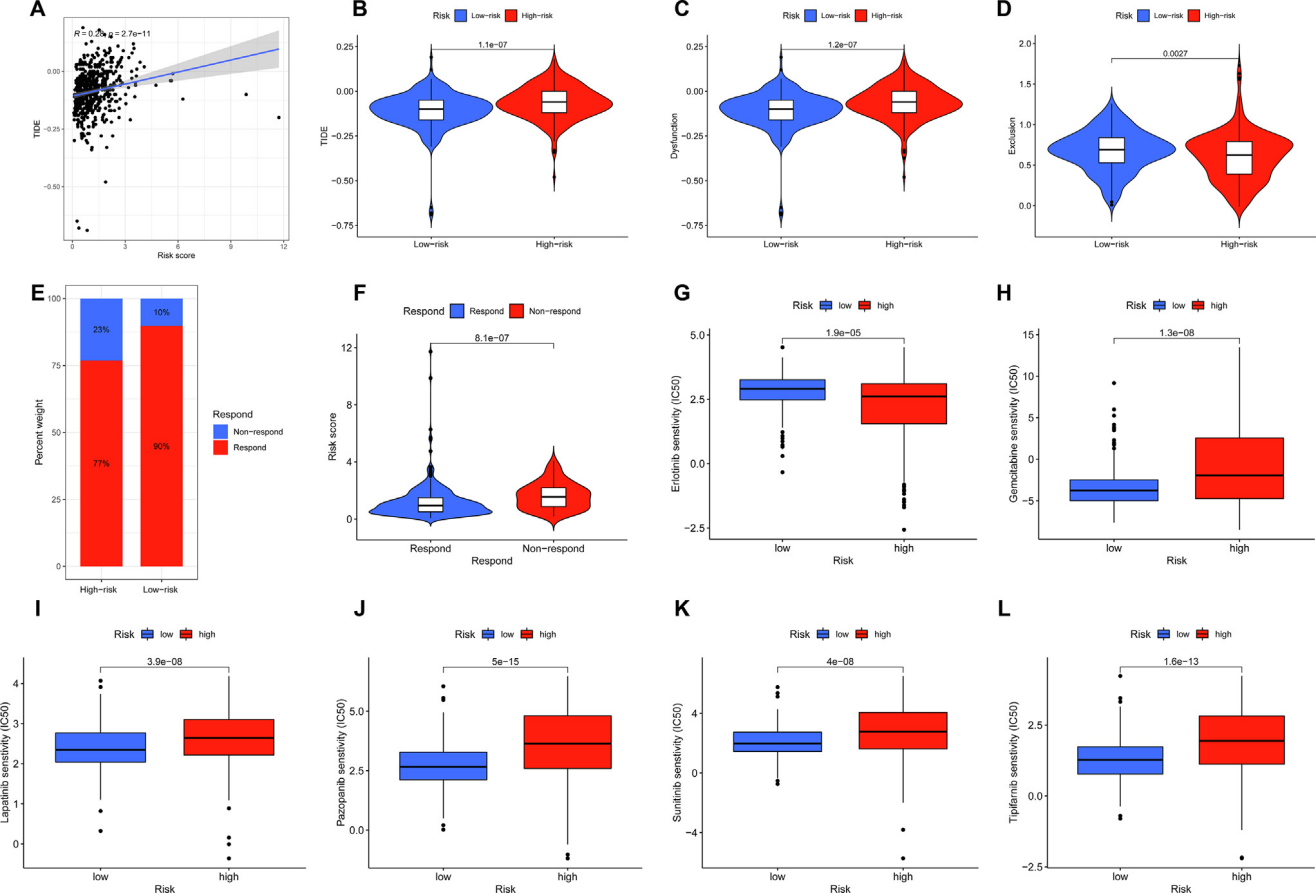
**Immunotherapy response and chemotherapeutic response. (A)** Relationship between risk score and TIDE score. Comparison of the TIDE score **(B)**, dysfunction score **(C)** and exclusion score **(D)** in the high- and low-risk groups. **(E)** The proportion of the immunotherapeutic responders between the high- and low-risk groups. **(F)** Comparison of risk score between respond group and non-respond group. **(G-L)** Sensitivity analysis of ommon chemotherapeutic drugs of KIRC in he high- and low-risk groups.

We assessed the predictive capability of our risk model in the immunotherapy response using the TIDE algorithm, and the detailed output of TIDE algorithm of TCGA-KIRC was shown in Supplementary Table 4. The result revealed that risk score was positively correlated with TIDE score (Fig. 11A) and the patients in the high-risk group had higher TIDE score (Fig. 11B), higher dysfunction score (Fig. 11C) and lower exclusion score (Fig. 11D) than the patients in the low-risk patients. Moreover, the patients in the high-risk group had a relatively lower proportion of immunotherapeutic responders compared with the low-risk group (77 % versus 90 %, Fig. 11E), and the patients who did not respond to immunotherapy had higher risk score when compared with patients who responded to immunotherapy (Fig. 11F). Those data suggested that the patients in the high-risk group had higher immune escape risk and lower immunotherapy response. For evaluation of chemotherapeutic response, we investigated the differential IC50 of chemotherapeutic drugs between the high- and low-risk groups by using pRRophetic algorithm, and we identified 86 kinds of chemotherapeutic drugs with differential IC50 between the high- and low-risk groups (Supplementary File 1). Fig. 11G–L showed differential IC50 of common chemotherapeutic drugs for KIRC, including erlotinib, gemcitabine, lapatinib, pazopanib, sunitinib and tipifarnib.

## 4 Discussion

Recently, a reserach by Tsvetkov et al. proposed a new form of copper-induced cell death. The report revealed that the overload intracellular copper can induce the aggregation of lipoylated dihydrolipoamide S-acetyltransferase (DLAT), which is associated with the mitochondrial tricarboxylic acid (TCA) cycle, resulting in proteotoxic stress and leading to a novel form of cell death termed cuproptosis.^4^ Tsvetkov et al. firstly found that elesclomol, a copper ionophore, was used to treat cancer, killed cells when loaded with copper, whereas elesclomol alone did not induce cell death, suggesting that cell death was caused by copper toxicity, which implied that targeted copper toxicity, or cuproptosis might serve as a therapeutic component in antitumor strategies.^4^, ^21^, ^22^

lncRNAs have been shown to participate in various considerable biological processes, such as cell proliferation and differentiation, gene regulation and translation, RNA splicing, regulation of microRNAs, and protein folding.^23^ Yang et al. in 2021 verified that the lncRNAs, FGD5-AS1 and AL391121.1 expression were decreased in VHL mutant tissues compared with VHL non-mutant tissues. Moreover, high expression group of FGD5-AS1 had significantly longer OS and DFS than their respective low expression groups in VHL mutant KIRC, confirming that lncRNA FGD5-AS1 was significantly associated with VHL and can serve as a novel biomarker of KIRC.^24^ Song et al. proved that lncRNA expression profiling data, the overall analysis revealed that two novel lncRNA signatures (DNM1P35 and MIR155HG) were found to be significantly correlated with KIRC patient's overall analysis.^25^ Those meant that lncRNAs might play an important role in the prognosis in patients with KIRC.

Ferroptosis was reported to be closely associated with the biological process of KIRC, influencing its proliferation, invasion, and metastasis, in which crucial regulatory roles of lncRNAs were identified in the ferroptosis-related biological process of malignant tumor cells.^26^, ^27^ Zhou et al. verified 8 ferroptosis-related lncRNAs to identify the prognosis of patients with KIRC.^28^ Tang et al. filtered 14 pyroptosis-Related lncRNAs with different expression patterns that were linked to KIRC prognosis. What’s more, by using Kaplan–Meier analysis, they identified a signature of high-risk lncRNAs relatedto poor prognosis for KIRC.^12^ Bian et al. functionally analyses exhibited that pathways related to TCA cycle were enriched, and the cuproptosis-related genes were also proven to be associated with the grading and staging of KIRC.^9^ But they have not analysised the cuproptosis-related lncRNAs in predicting the prognosis in patients with KIRC yet.

Zheng et al. in 2021 identified lncRNA signatures that were associated with LSCC patient survival outcomes. They revealed prognostic capabilities for the lncRNAs, which suggested that LINC02555, APCDD1L-DT and OTX2-AS1 might be significantly associated with patient survival.^29^ It suggested lncRNAs could predicted the prognosis of cancer. In our study, we firstly filtered 13 differential CRGs (ATP7B, CDKN2A, DBT, DLAT, DLD, DLST, FDX1, LIAS, MTF1, NFE2L2, PDHA1, PDHB and SLC31A1) in KIRC, and further explored 8-CRlncRNAs (AC004148.1, AC006116.9, AC124854.1, APCDD1L-DT, FAM225B, LINC00886, LINC00944 and ZEB2-AS1) risk model to predict the prognosis of KIRC patient. Secondly, we validated the accuracy and sensitivity of this risk model. Thirdly, tumor microenvironment analysis indicated that immune was related with prognosis of KIRC and the risk model we constructed could be use to assess immune. Then, we analyzed relationship between risk score and immune cell infiltration, immune function and gene mutation, respectively. Moreover, we also assessed the immunotherapy response and chemotherapeutic response in this risk model. Lastly, we concluded that the 8-CRlncRNAs risk model we established might predict the prognosis and therapy of KIRC.

## 5 Conclusions

The 8-CRlncRNAs risk model was identified to predict the prognoses, tumor immune, immunotherapy response and chemotherapeutic response in KIRC patients. There were several shortcomings in our research. Firstly, we validated that there are 8 CRlncRNAs related prognosis and therapy, there was no data set with sufficient sample size and clinical prognosis information for further verification, which is urgently warranted in future research. Secondly, although the prognostic score focusing on the CRlncRNAs expression signature showed a good performance in the prediction of KIRC, some other significant lncRNAs with predictive values were not considered in this study, which also needed further study. Thirdly, considering the prognostic signature was built and validated by exploiting data from public databases, more biological evidence was needed besides the statistical evidence we offered. Even so, this study systematically analyzed of CRlncRNAs in KIRC and our results would also provide novel insights and therapeutic strategies related to cuproptosis for cancer prevention and treatment.

## Availability of data and material

All data generated or analyzed during this study are included in this published article.

## Authors' contributions

YH and YW was a major contributor to writing the manuscript. YW and HZ was a major contributor to the bioinformatics analysis. JL, HZ, FW and GZ contribute equally to this work. The authors read and approved the final manuscript.

## Declaration of competing interest

The authors declare that they have no known competing financial interests or personal relationships that could have appeared to influence the work reported in this paper.

## Appendix A Supplementary material

Supplementary Table 1 7795 differentially expressed lncRNAs in the TCGA-KIRC patients.

Supplementary Table 2 3118 lncRNAs which were highly correlated with overall survival.

Supplementary Table 3 711 differentially expressed genes between high-risk group and low-risk group.

Supplementary Table 4 TIDE algorithm of TCGA-KIRC patients.
