# Supplementary figures and images for: Identification of cuproptosis-related lncRNAs signature for predicting the prognosis in patients with kidney renal clear cell carcinoma
Source: J Genet Eng Biotechnol. 2024 Jan 30;22(1):100338. doi: 10.1016/j.jgeb.2023.100338 (PMC10860879; doi:10.1016/j.jgeb.2023.100338)

Risk 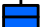 low 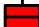 high

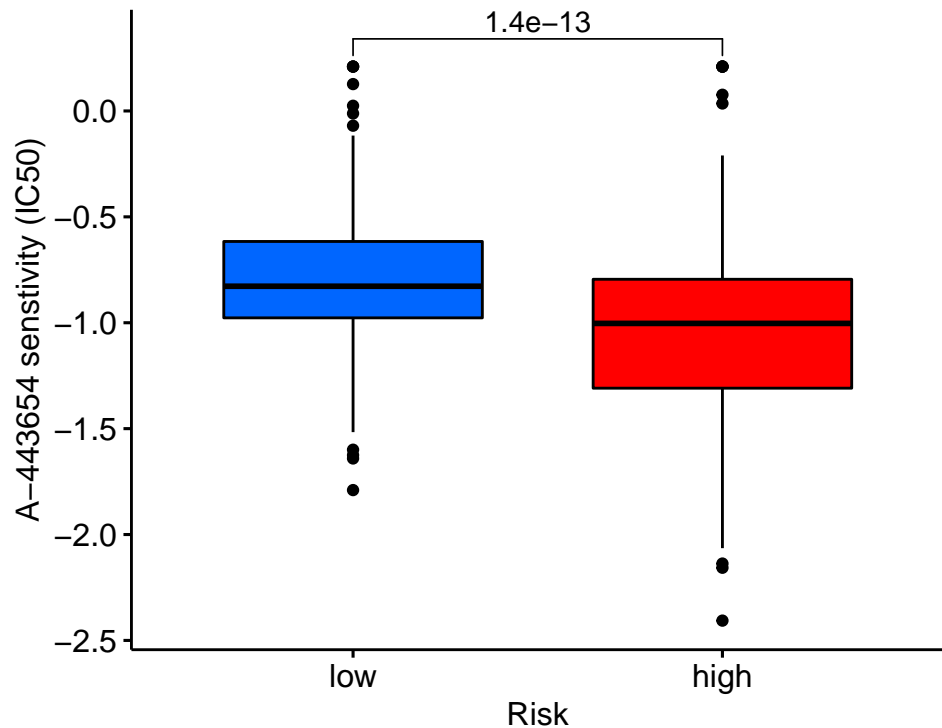

Supplement: Supplementary data 5 [file mmc5.zip › durgSenstivity.A-443654.pdf]

Risk 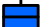 low 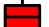 high

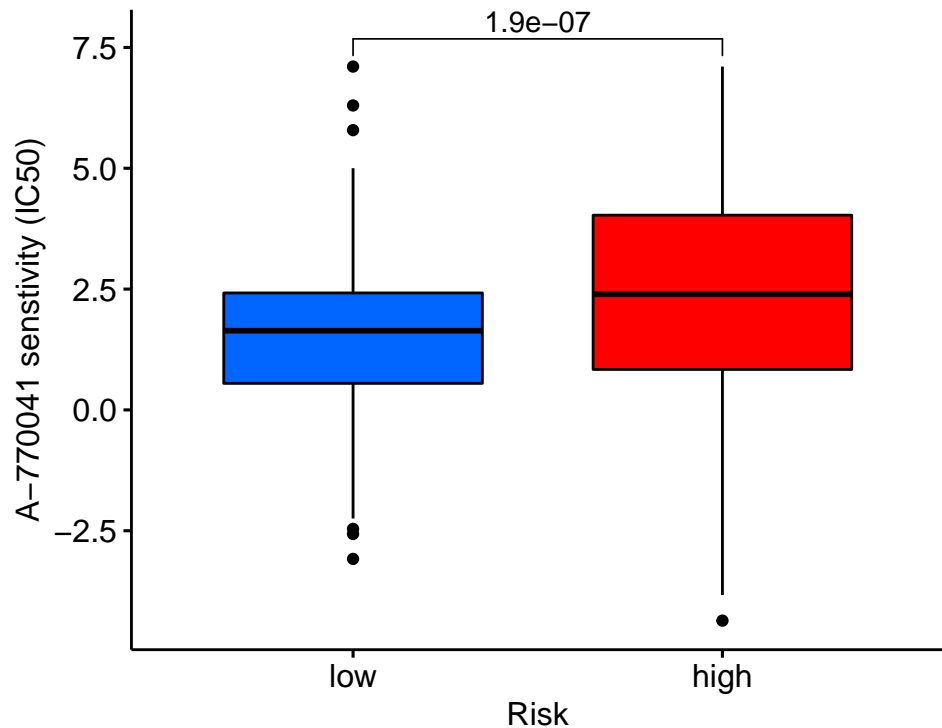

Supplement: Supplementary data 5 [file mmc5.zip › durgSenstivity.A-770041.pdf]

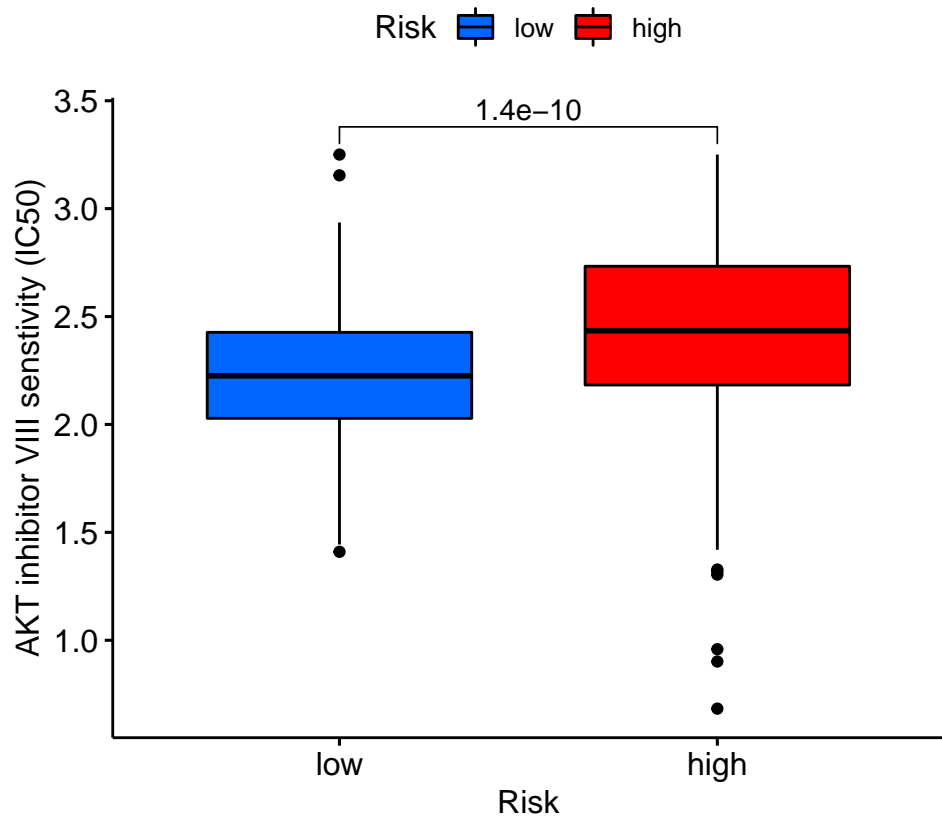

Supplement: Supplementary data 5 [file mmc5.zip › durgSenstivity.AKT inhibitor VIII.pdf]

AP-24534 sensitivity (IC50)

Risk 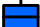 low 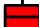 high

$2.7\text{e-}11$

low

high

Risk

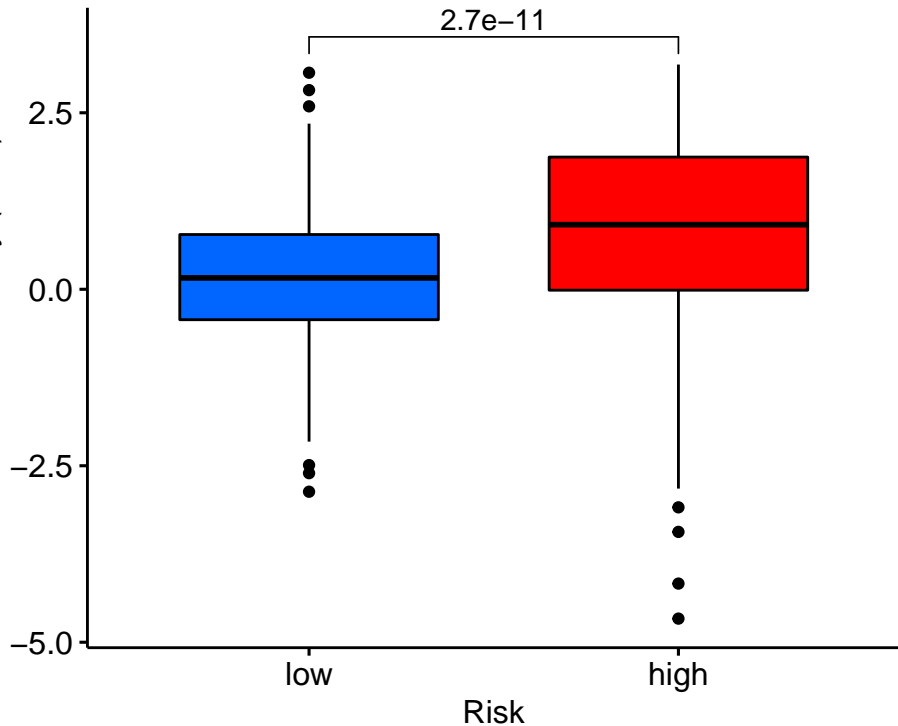

Supplement: Supplementary data 5 [file mmc5.zip › durgSenstivity.AP-24534.pdf]

Risk 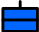 low 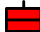 high

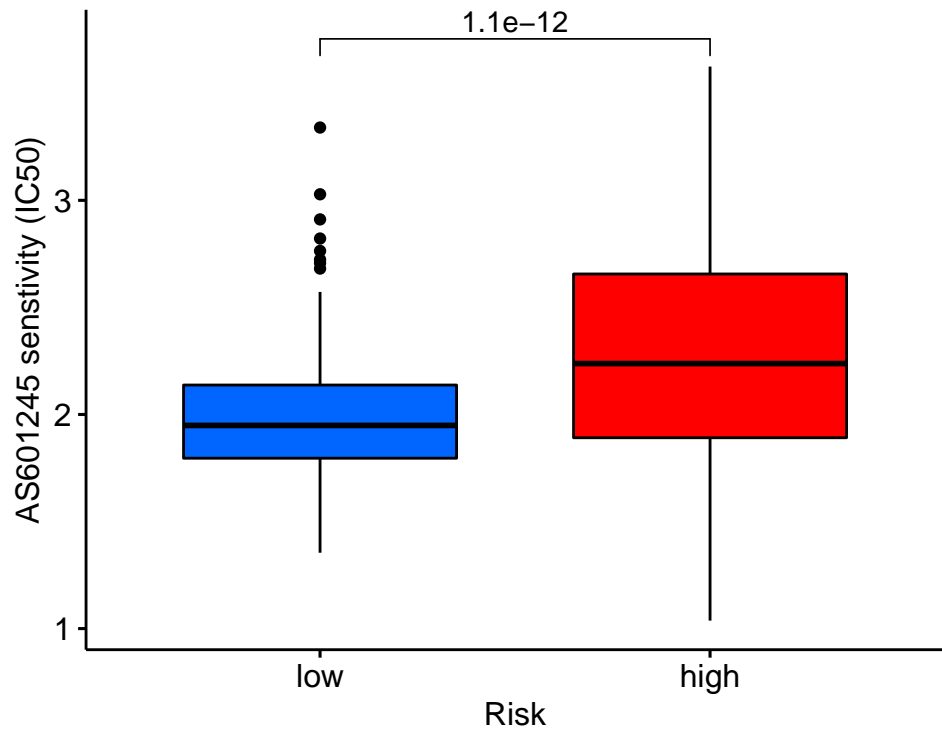

Supplement: Supplementary data 5 [file mmc5.zip › durgSenstivity.AS601245.pdf]

Risk 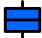 low 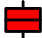 high

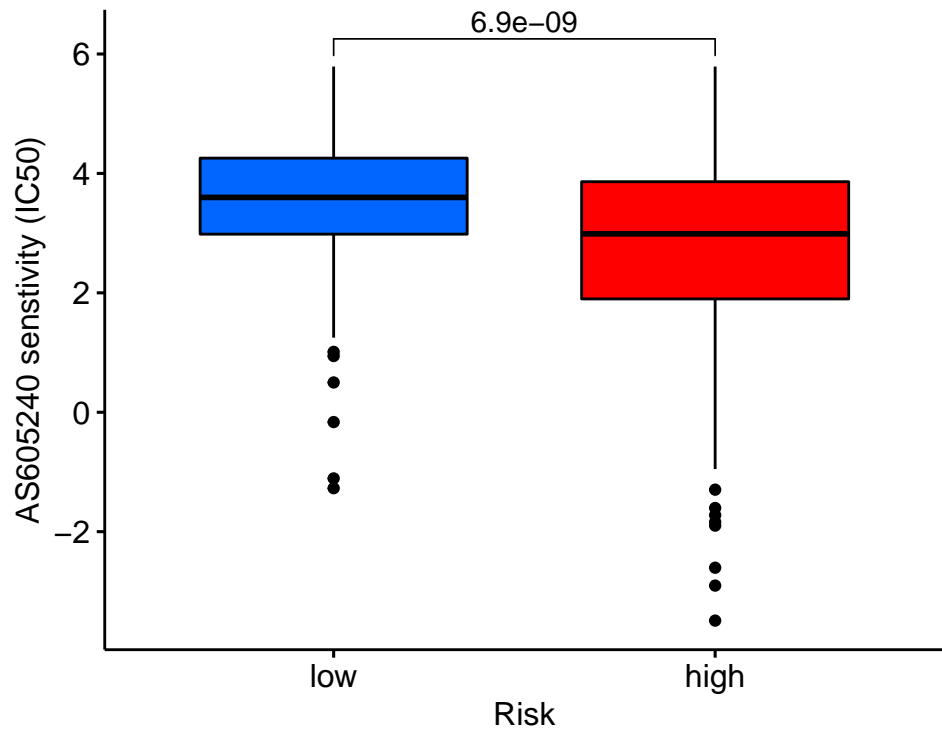

Supplement: Supplementary data 5 [file mmc5.zip › durgSenstivity.AS605240.pdf]

Risk 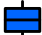 low 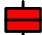 high

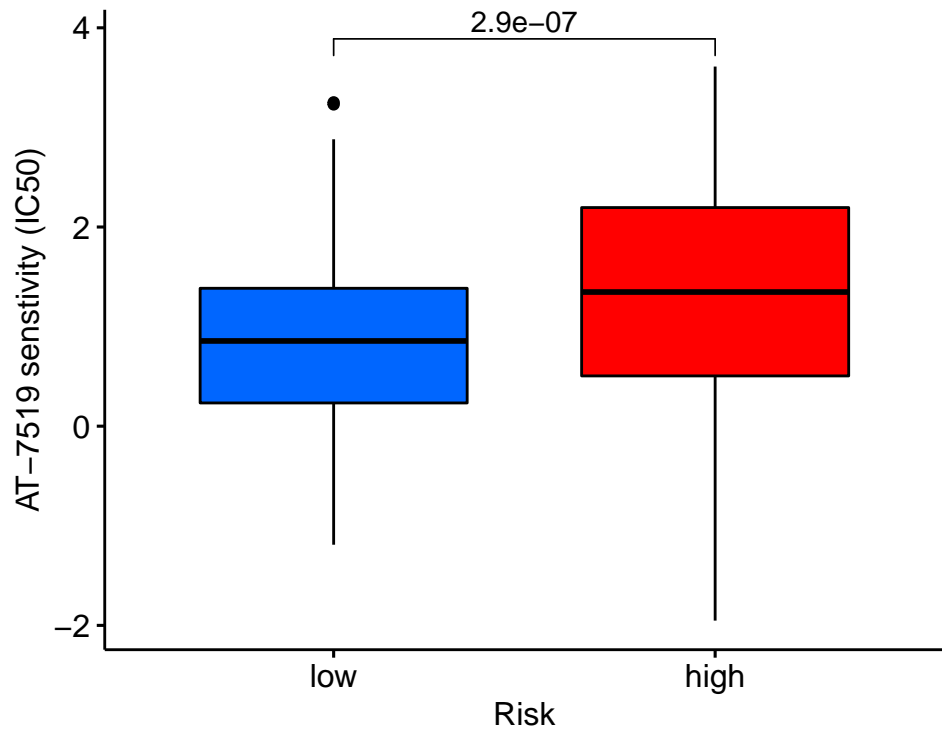

Supplement: Supplementary data 5 [file mmc5.zip › durgSenstivity.AT-7519.pdf]

Risk 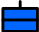 low 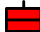 high

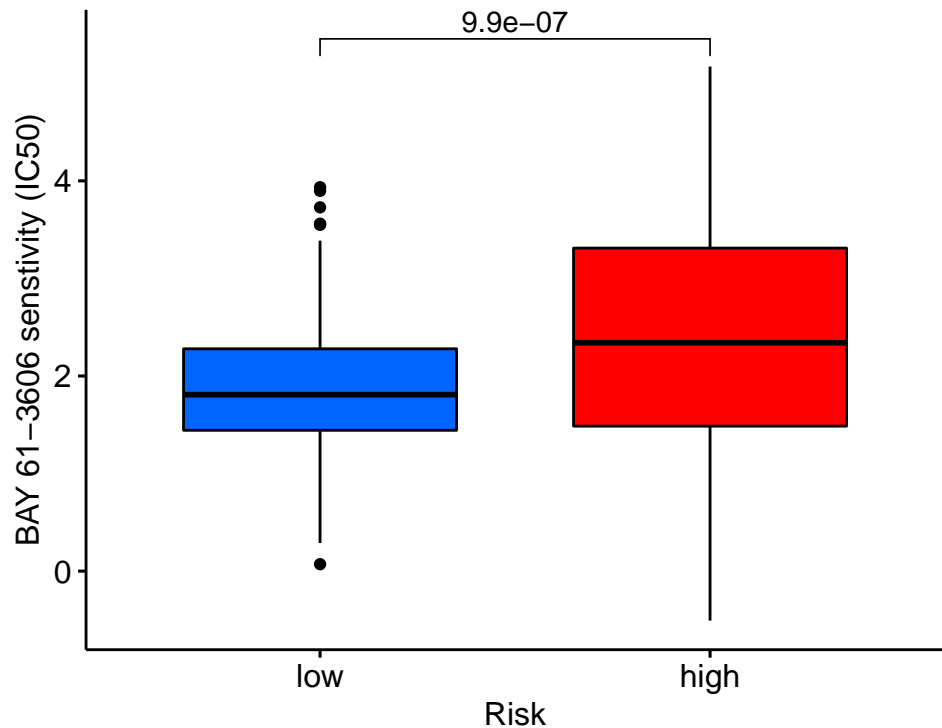

Supplement: Supplementary data 5 [file mmc5.zip › durgSenstivity.BAY 61-3606.pdf]

Risk 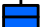 low 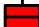 high

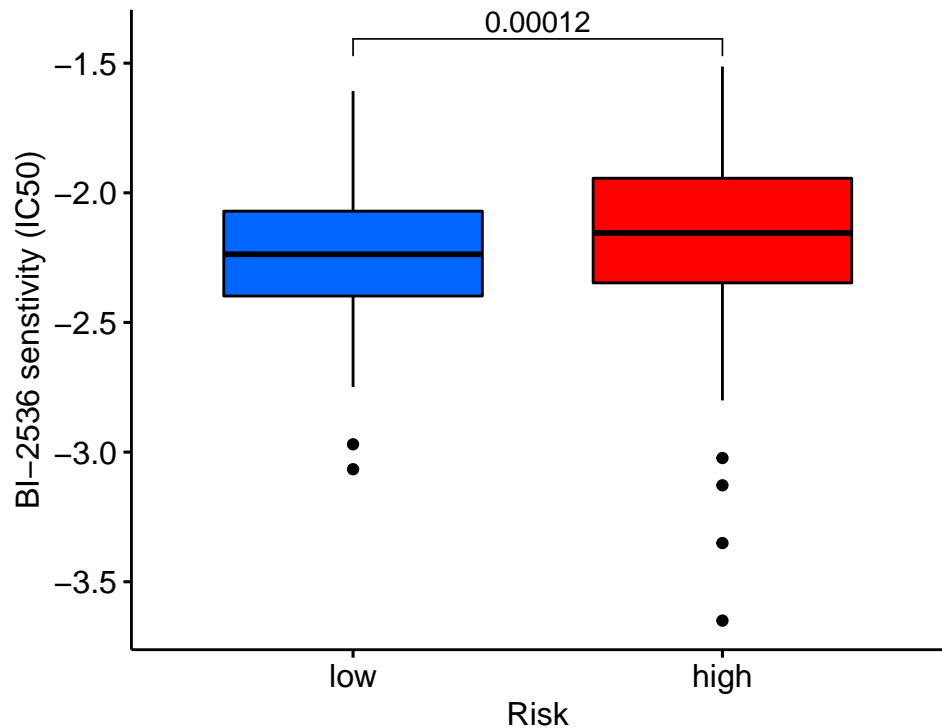

Supplement: Supplementary data 5 [file mmc5.zip › durgSenstivity.BI-2536.pdf]

Risk 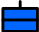 low 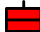 high

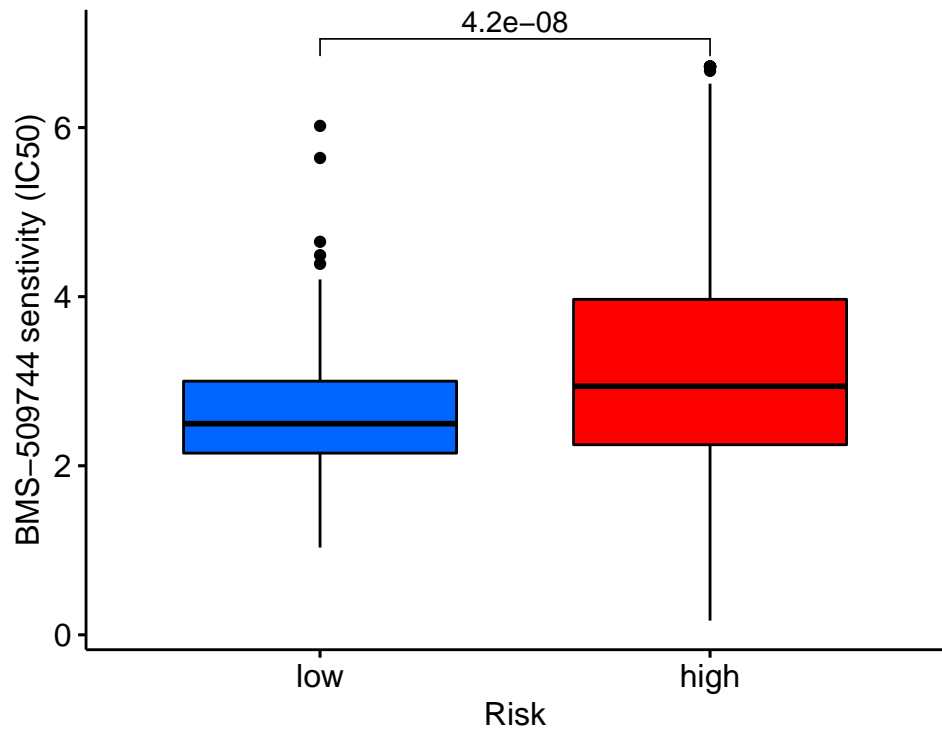

Supplement: Supplementary data 5 [file mmc5.zip › durgSenstivity.BMS-509744.pdf]

BMS-754807 sensitivity (IC50)

Risk low high

0.00079

low

high

Risk

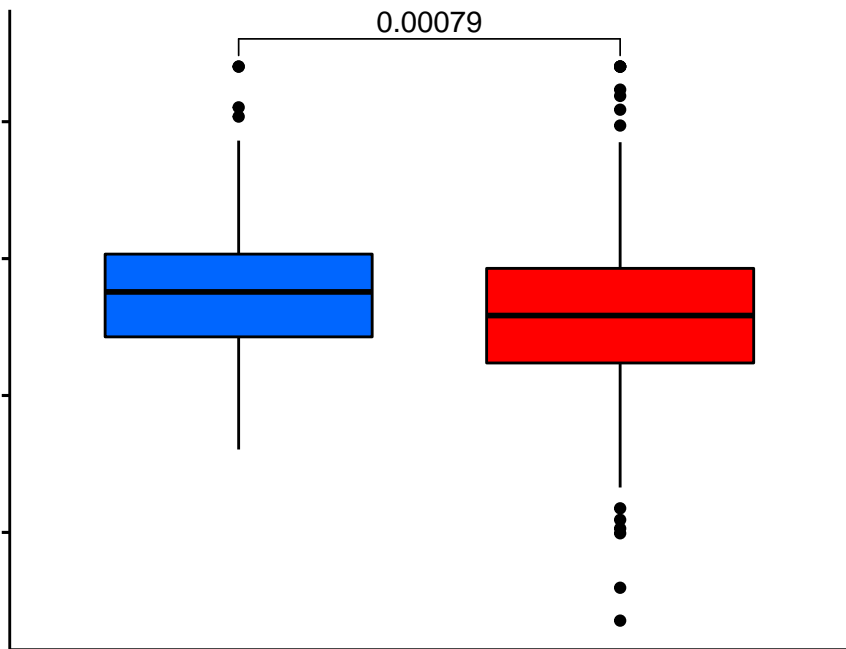

Supplement: Supplementary data 5 [file mmc5.zip › durgSenstivity.BMS-754807.pdf]

Risk 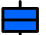 low 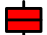 high

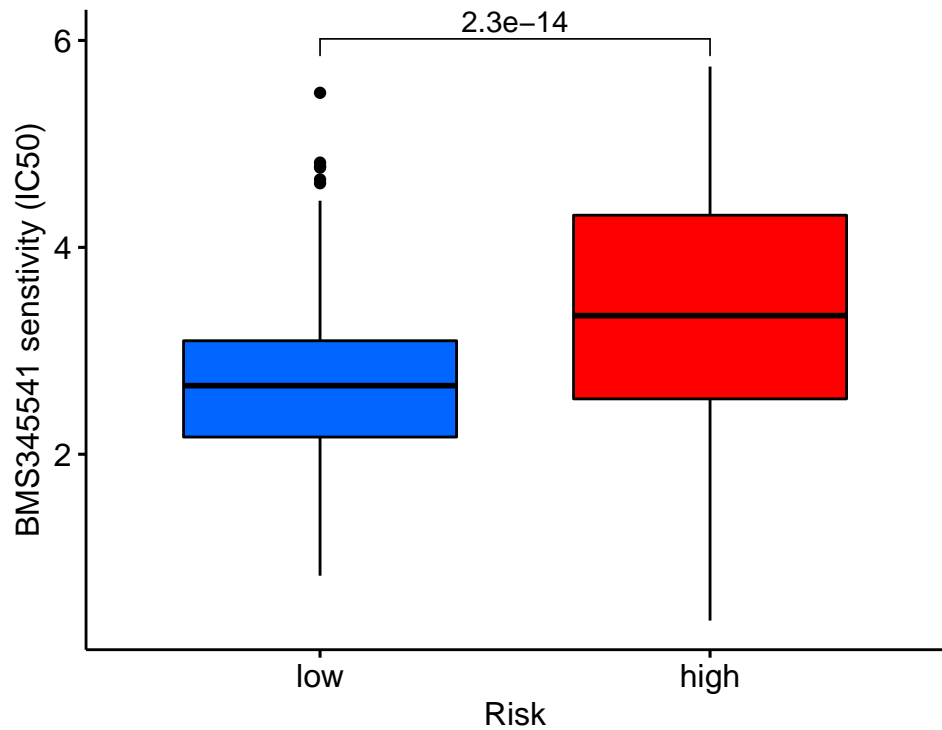

Supplement: Supplementary data 5 [file mmc5.zip › durgSenstivity.BMS345541.pdf]

Risk 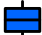 low 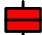 high

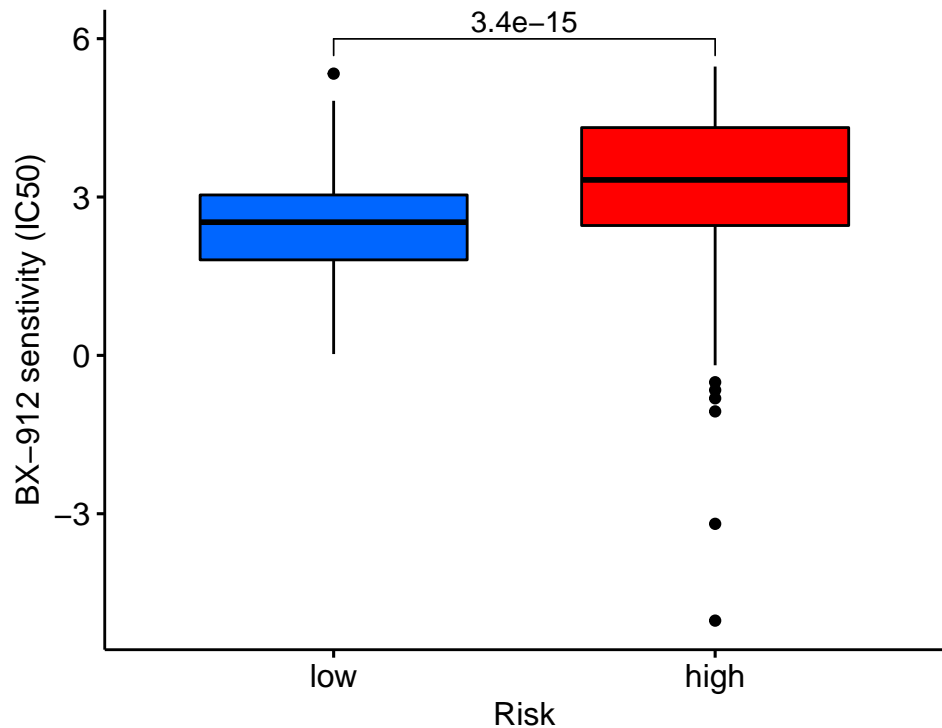

Supplement: Supplementary data 5 [file mmc5.zip › durgSenstivity.BX-912.pdf]

Risk 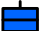 low 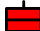 high

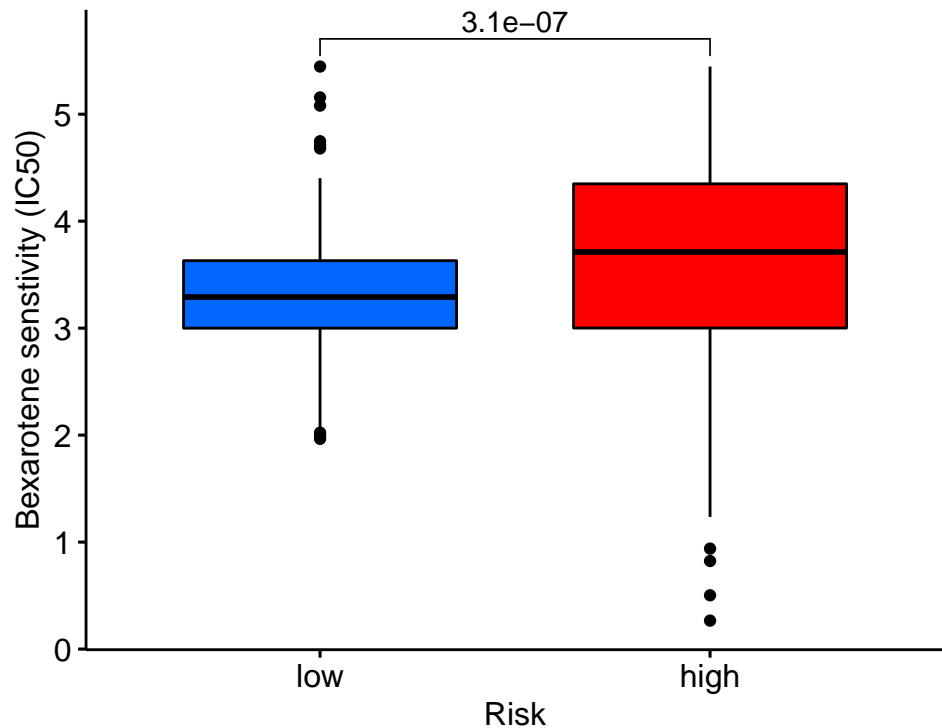

Supplement: Supplementary data 5 [file mmc5.zip › durgSenstivity.Bexarotene.pdf]

Risk 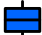 low 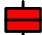 high

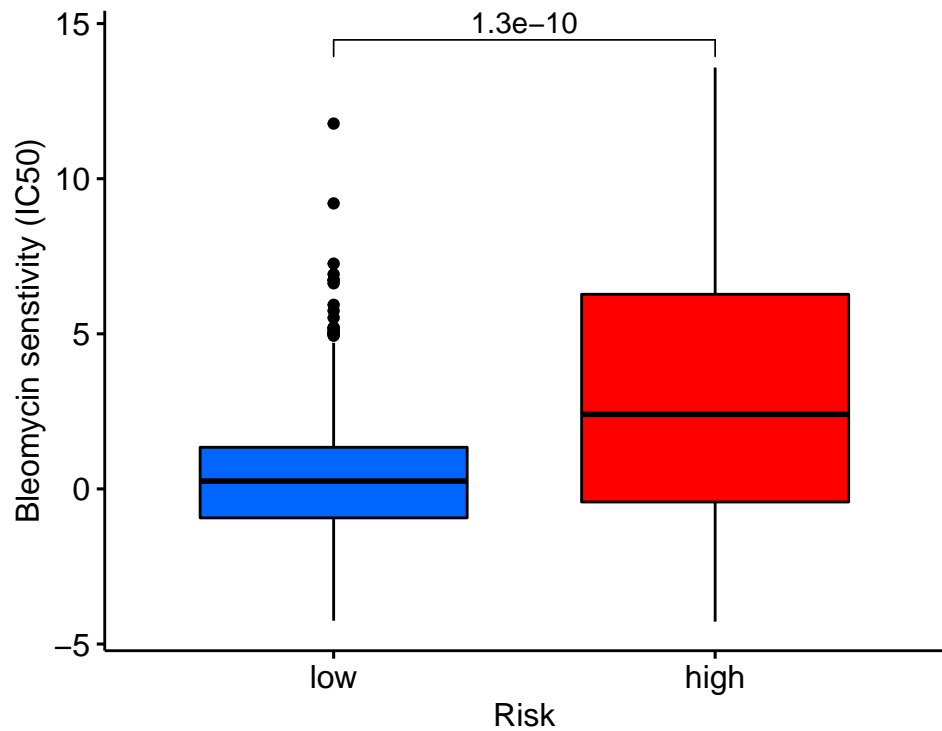

Supplement: Supplementary data 5 [file mmc5.zip › durgSenstivity.Bleomycin.pdf]

Risk 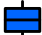 low 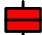 high

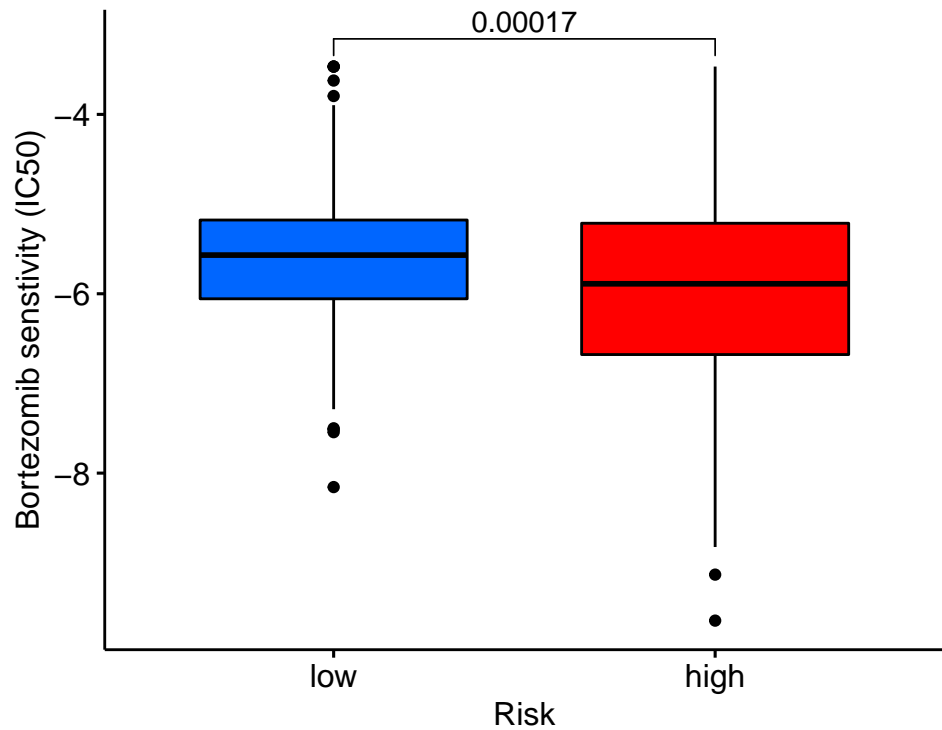

Supplement: Supplementary data 5 [file mmc5.zip › durgSenstivity.Bortezomib.pdf]

Risk 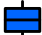 low 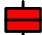 high

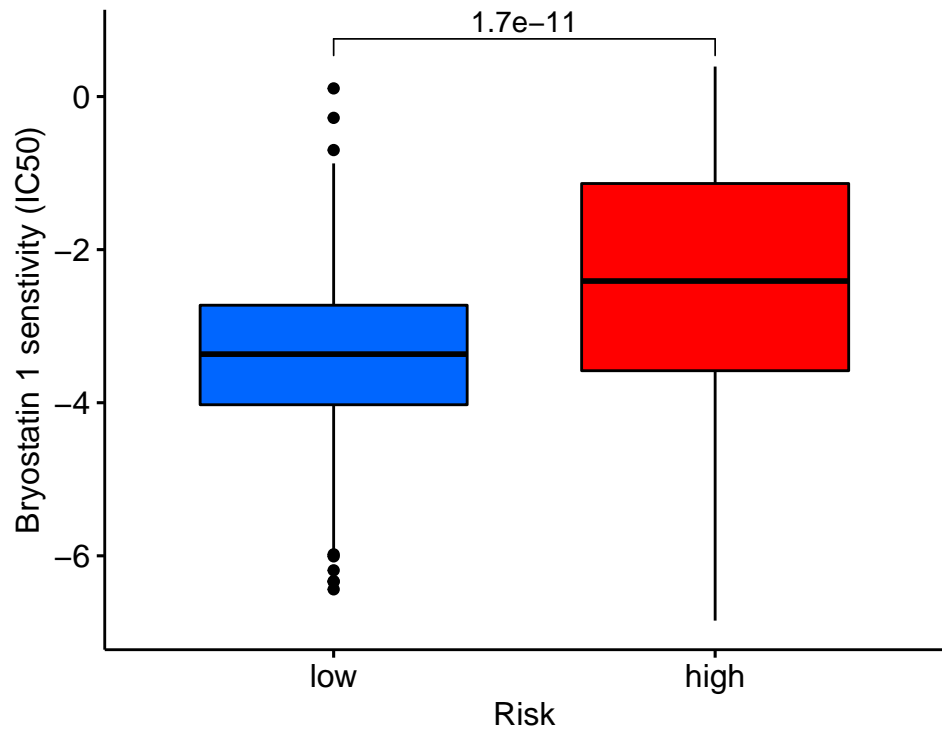

Supplement: Supplementary data 5 [file mmc5.zip › durgSenstivity.Bryostatin 1.pdf]

Risk 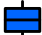 low 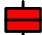 high

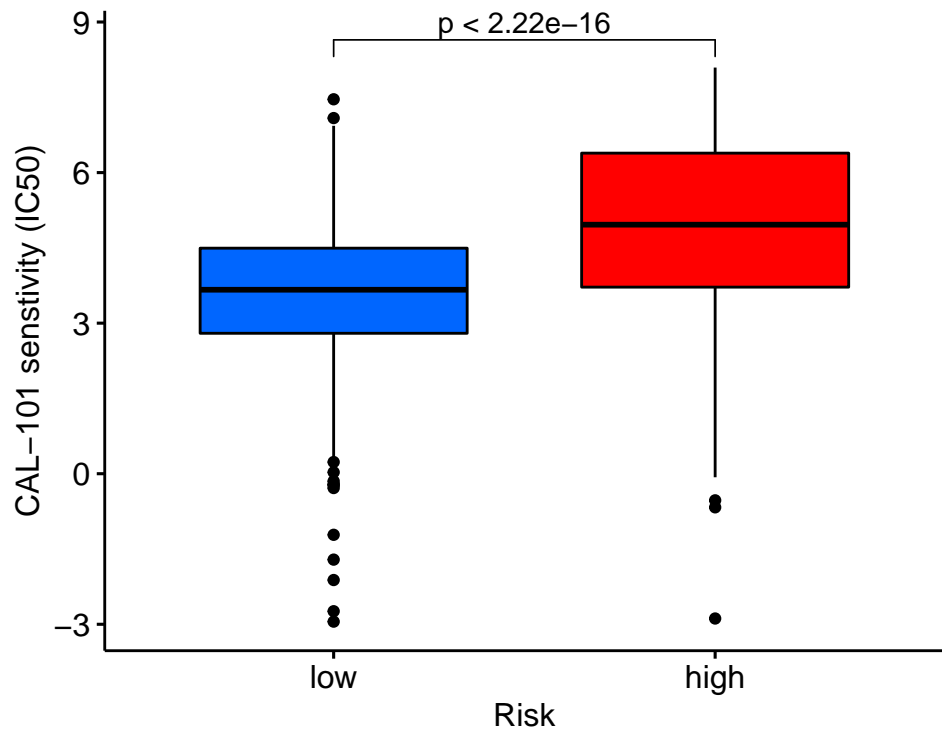

Supplement: Supplementary data 5 [file mmc5.zip › durgSenstivity.CAL-101.pdf]

Risk 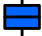 low 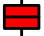 high

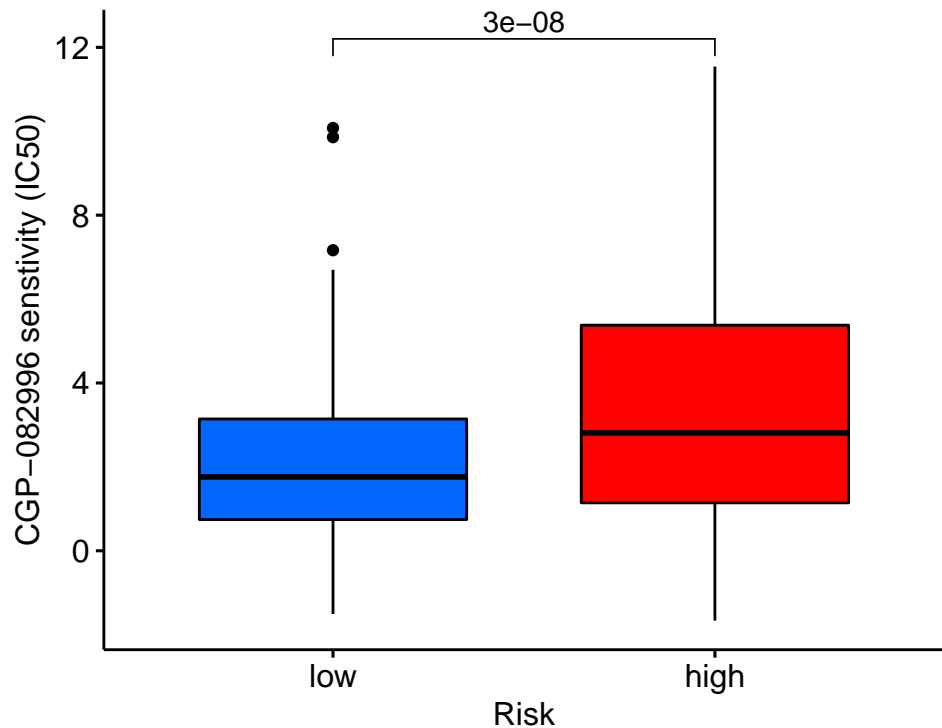

Supplement: Supplementary data 5 [file mmc5.zip › durgSenstivity.CGP-082996.pdf]

CGP-60474 sensitivity (IC50)

Risk 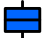 low 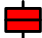 high

1.2e-05

low

high

Risk

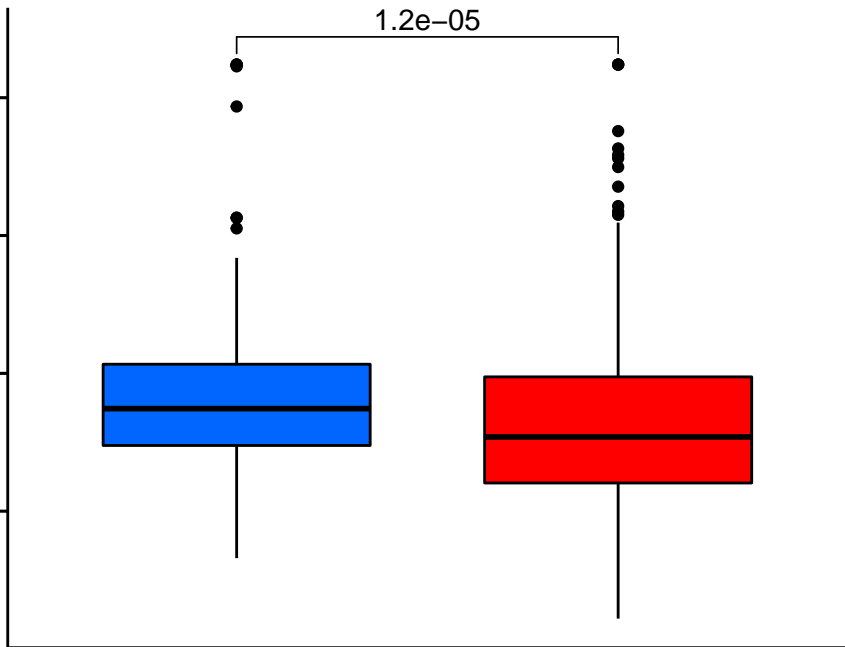

Supplement: Supplementary data 5 [file mmc5.zip › durgSenstivity.CGP-60474.pdf]

Risk 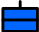 low 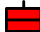 high

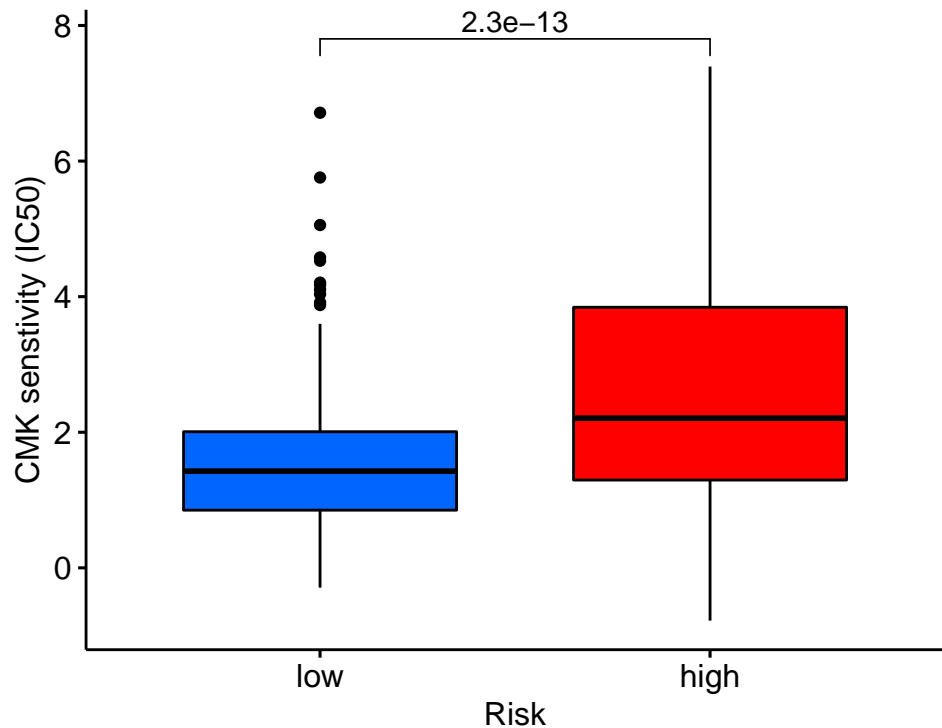

Supplement: Supplementary data 5 [file mmc5.zip › durgSenstivity.CMK.pdf]

Risk 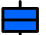 low 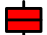 high

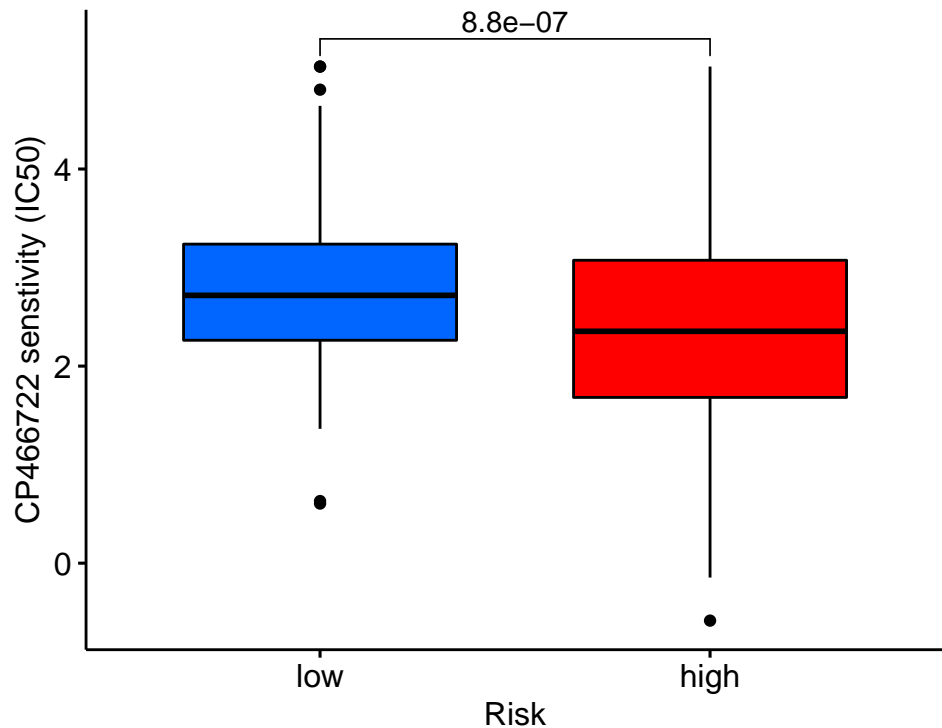

Supplement: Supplementary data 5 [file mmc5.zip › durgSenstivity.CP466722.pdf]

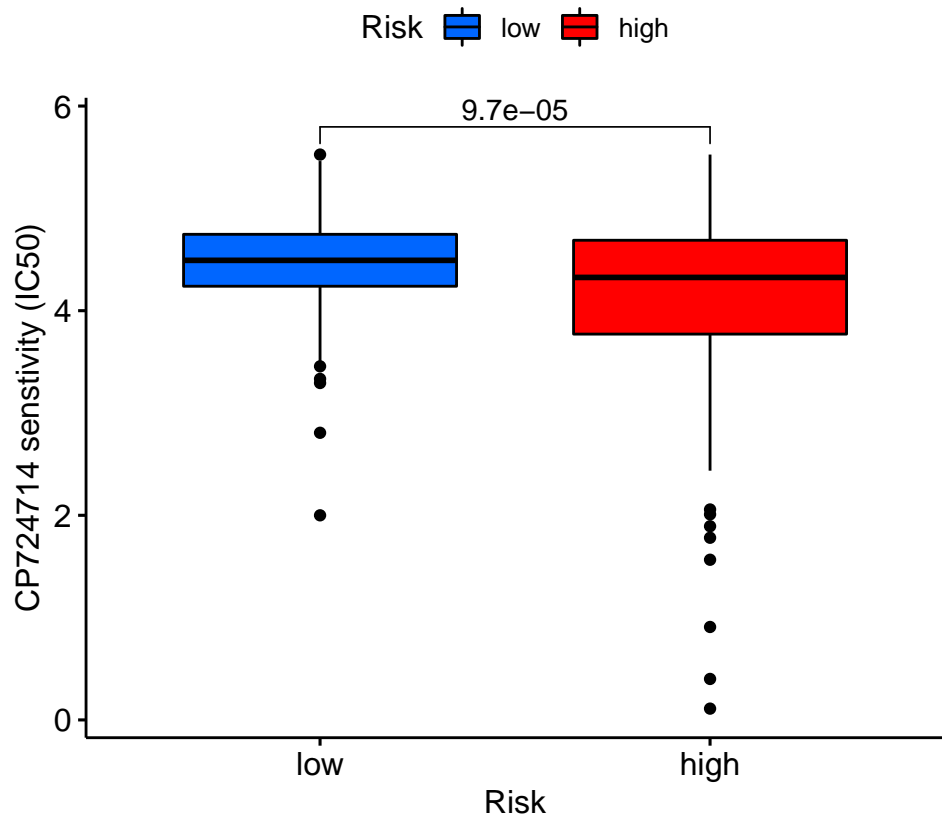

Supplement: Supplementary data 5 [file mmc5.zip › durgSenstivity.CP724714.pdf]

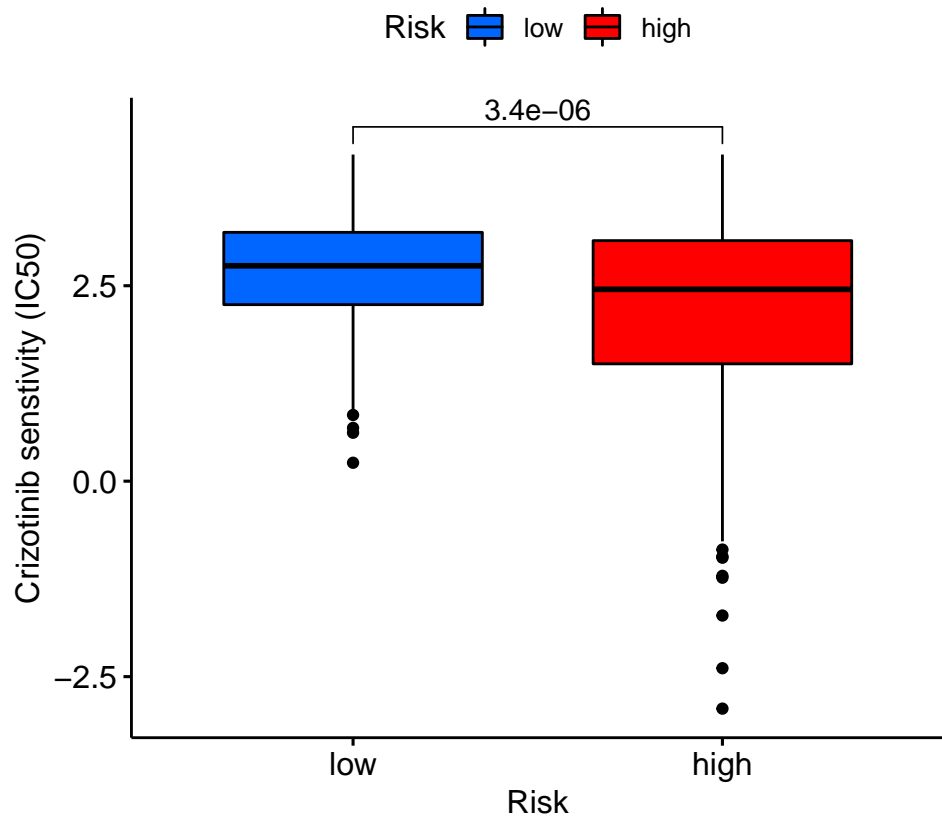

Supplement: Supplementary data 5 [file mmc5.zip › durgSenstivity.Crizotinib.pdf]

Risk 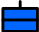 low 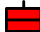 high

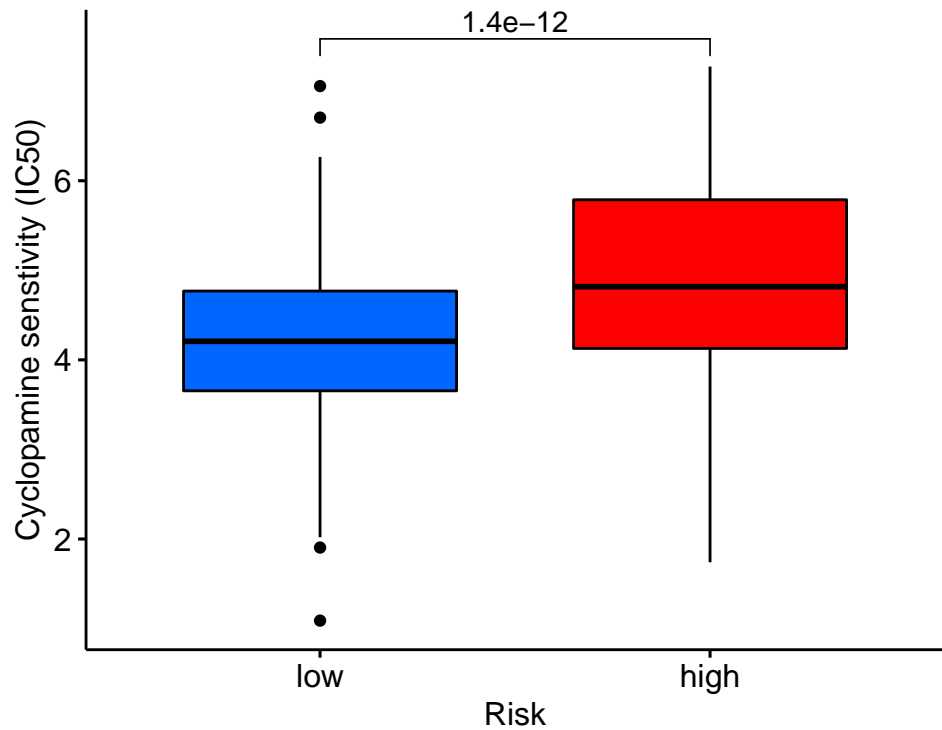

Supplement: Supplementary data 5 [file mmc5.zip › durgSenstivity.Cyclopamine.pdf]

Risk 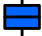 low 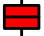 high

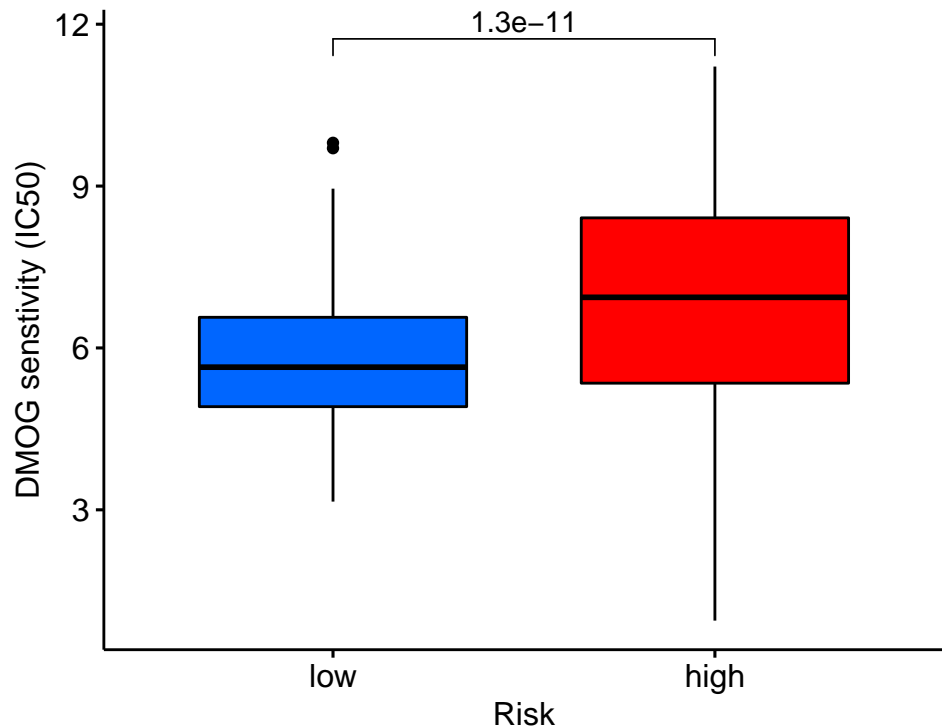

Supplement: Supplementary data 5 [file mmc5.zip › durgSenstivity.DMOG.pdf]

Risk 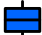 low 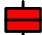 high

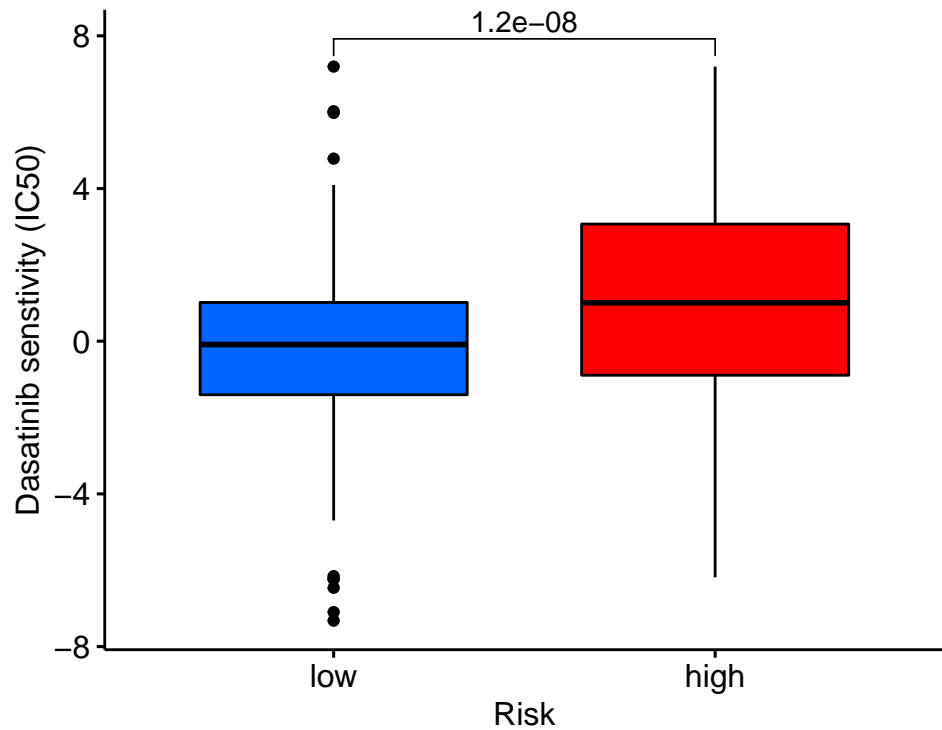

Supplement: Supplementary data 5 [file mmc5.zip › durgSenstivity.Dasatinib.pdf]

Epothilone B sensitivity (IC50)

Risk 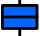 low 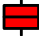 high

$2.1 \times 10^{-7}$

low

high

Risk

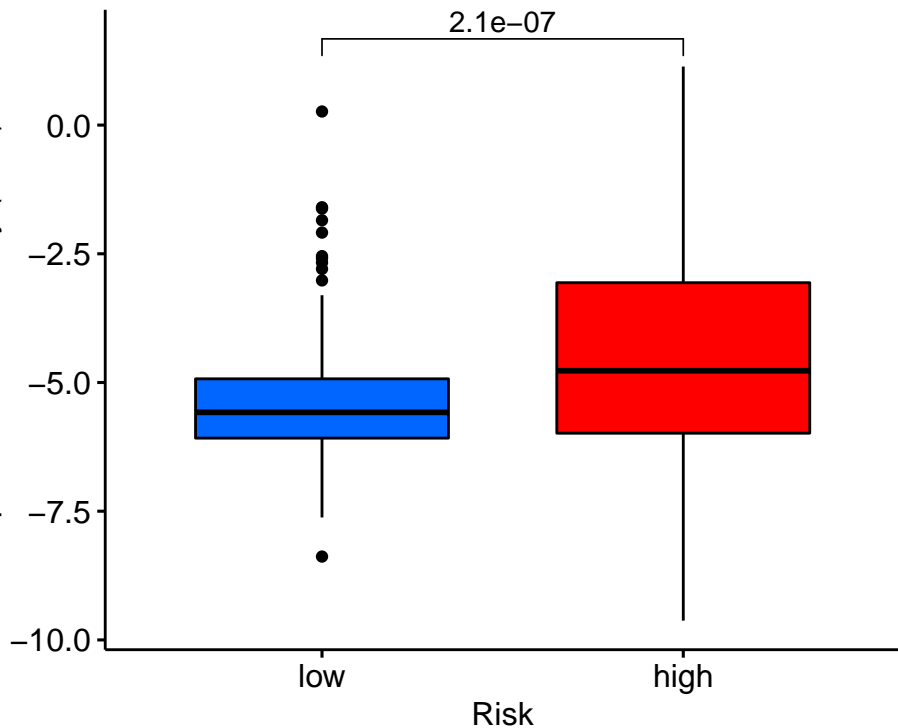

Supplement: Supplementary data 5 [file mmc5.zip › durgSenstivity.Epothilone B.pdf]

Risk 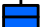 low 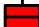 high

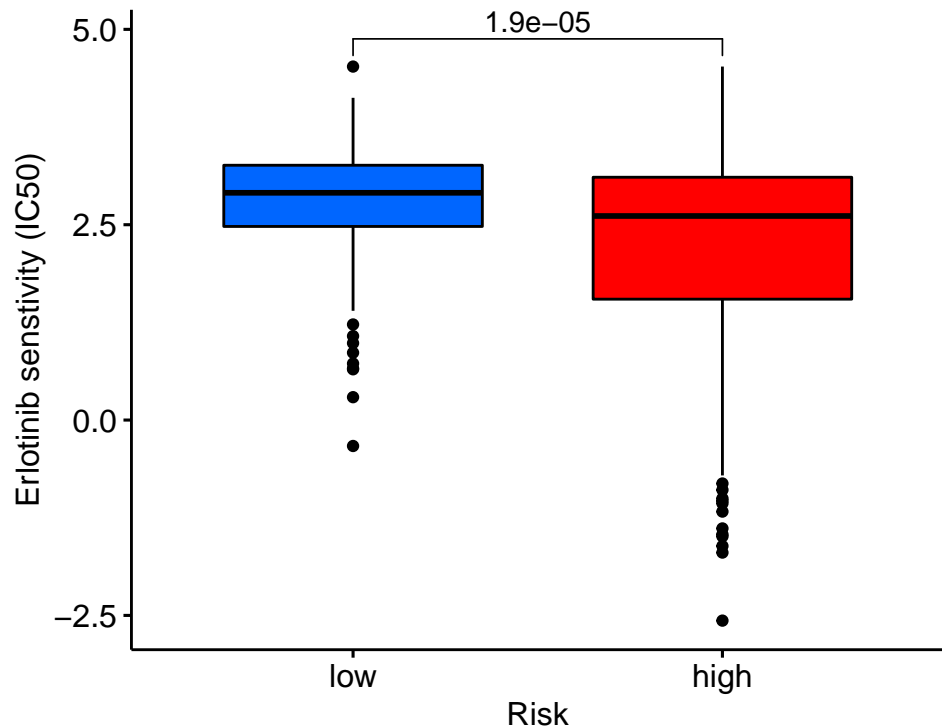

Supplement: Supplementary data 5 [file mmc5.zip › durgSenstivity.Erlotinib.pdf]

Risk 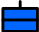 low 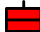 high

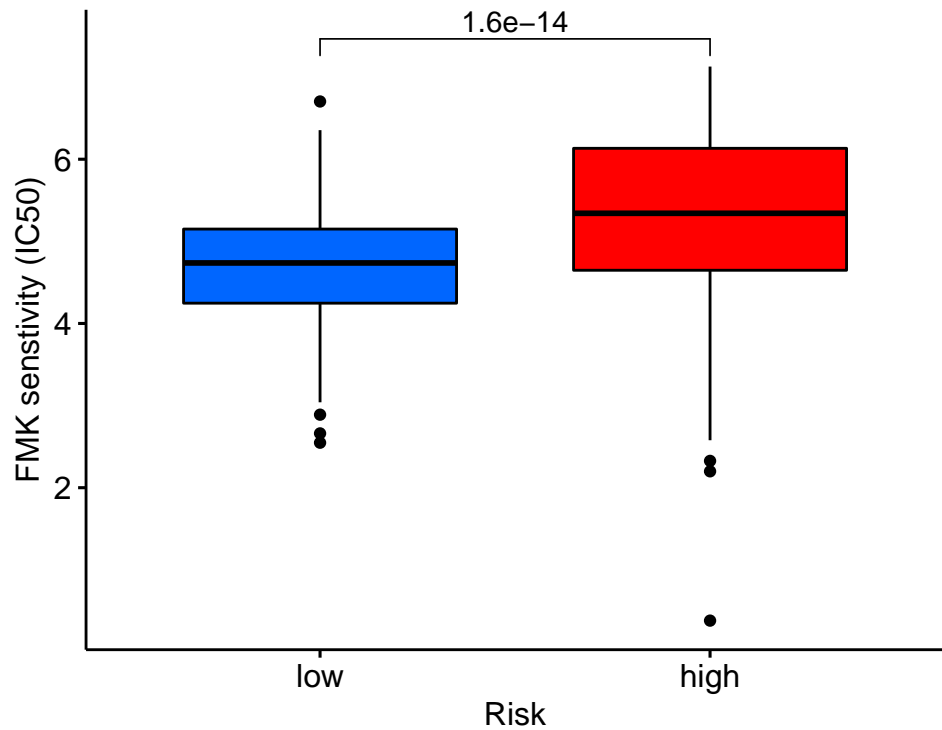

Supplement: Supplementary data 5 [file mmc5.zip › durgSenstivity.FMK.pdf]

Risk 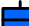 low 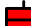 high

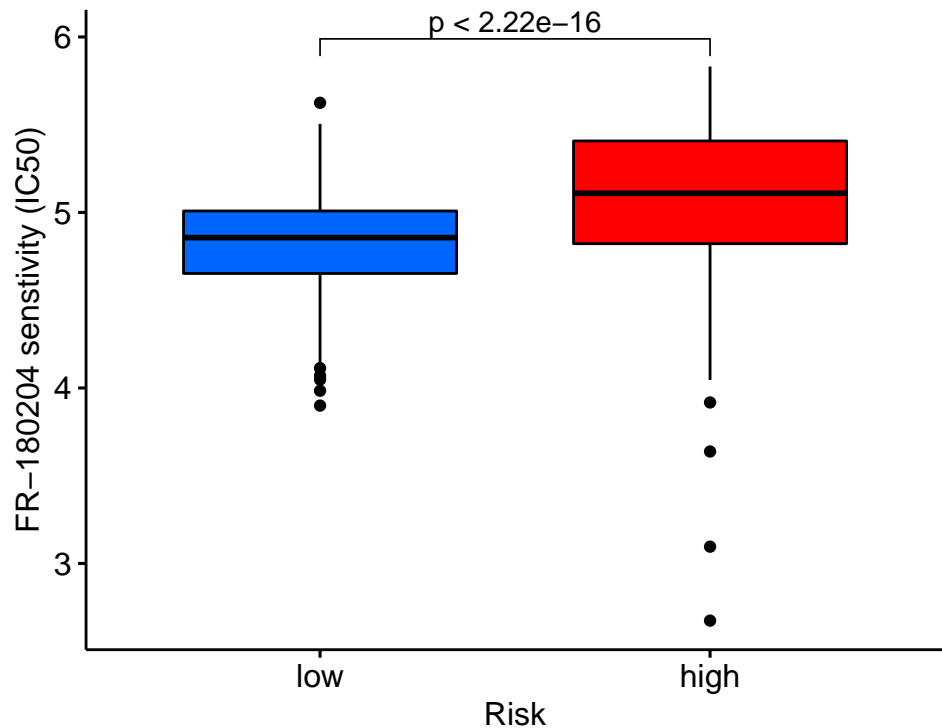

Supplement: Supplementary data 5 [file mmc5.zip › durgSenstivity.FR-180204.pdf]

Risk 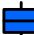 low 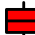 high

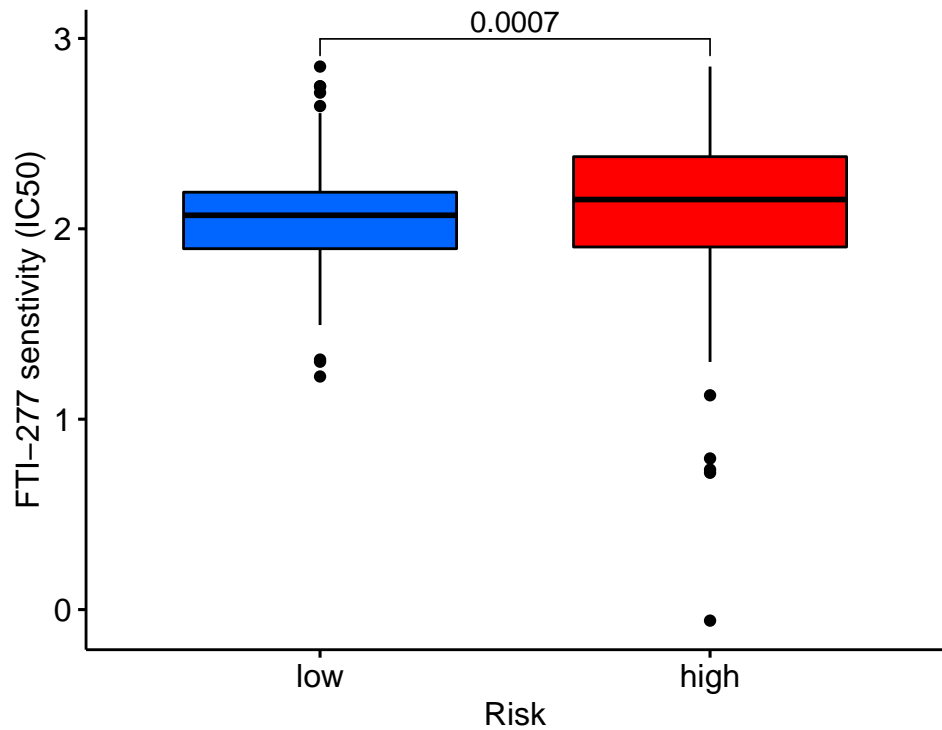

Supplement: Supplementary data 5 [file mmc5.zip › durgSenstivity.FTI-277.pdf]

GSK-650394 sensitivity (IC50)

Risk 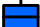 low 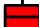 high

$3.1\text{e-}09$

low

high

Risk

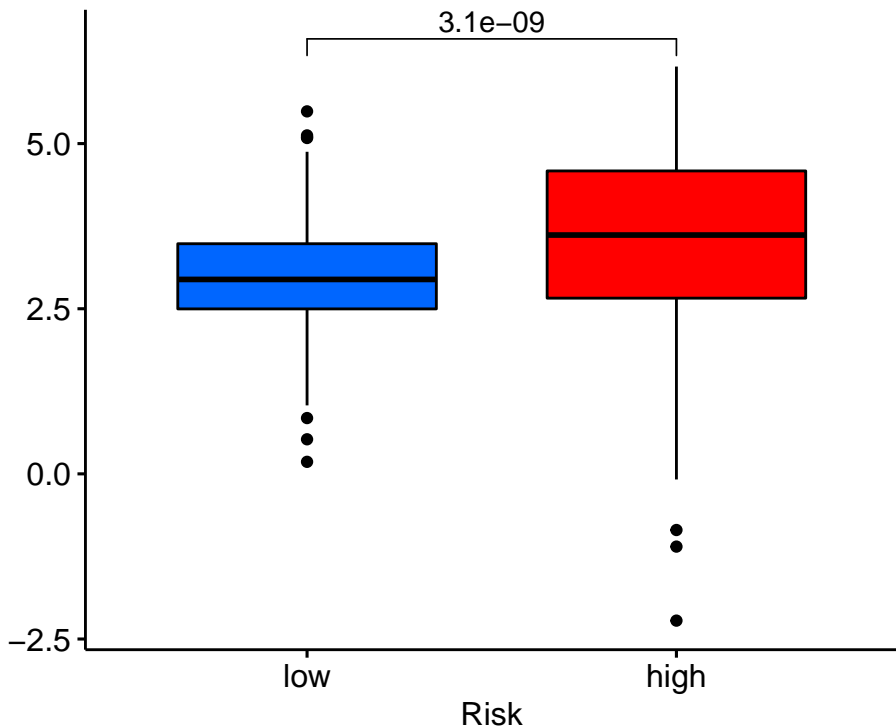

Supplement: Supplementary data 5 [file mmc5.zip › durgSenstivity.GSK-650394.pdf]

Risk 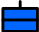 low 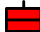 high

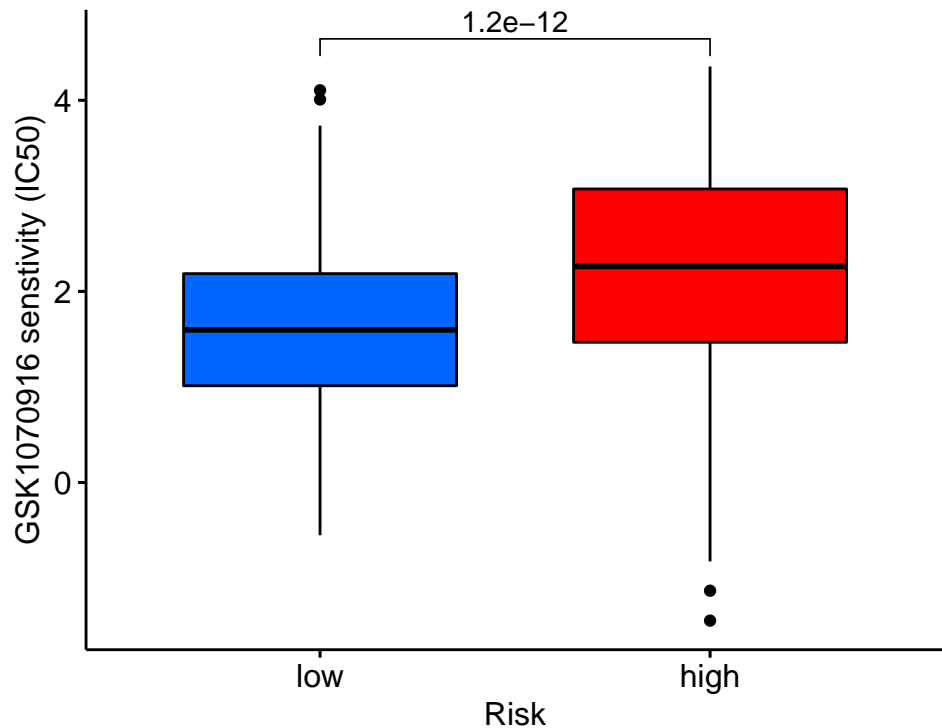

Supplement: Supplementary data 5 [file mmc5.zip › durgSenstivity.GSK1070916.pdf]

Risk 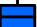 low 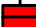 high

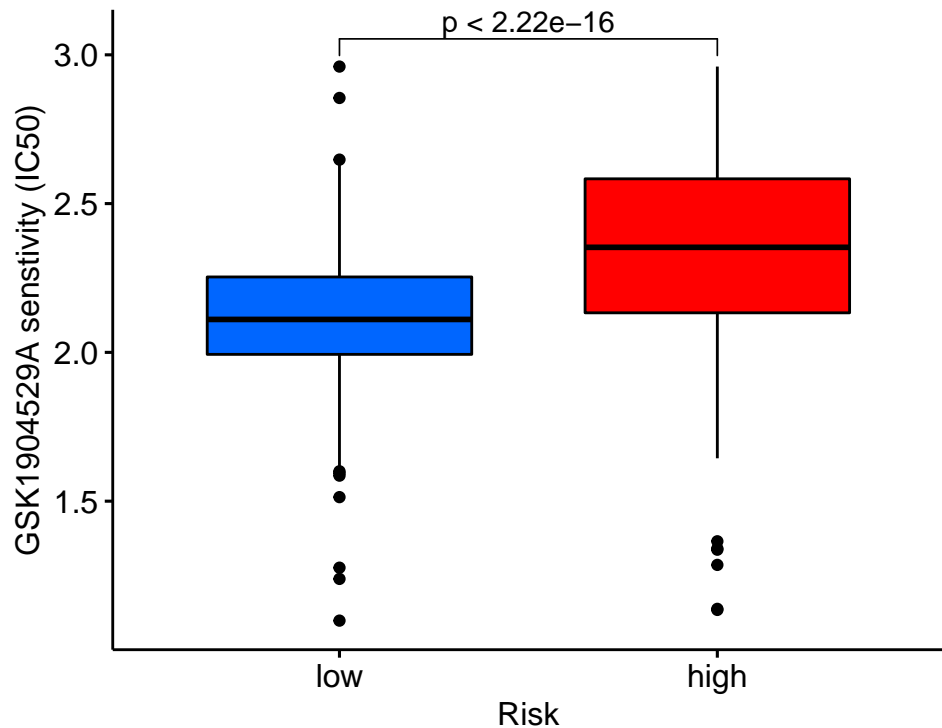

Supplement: Supplementary data 5 [file mmc5.zip › durgSenstivity.GSK1904529A.pdf]

Risk 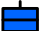 low 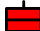 high

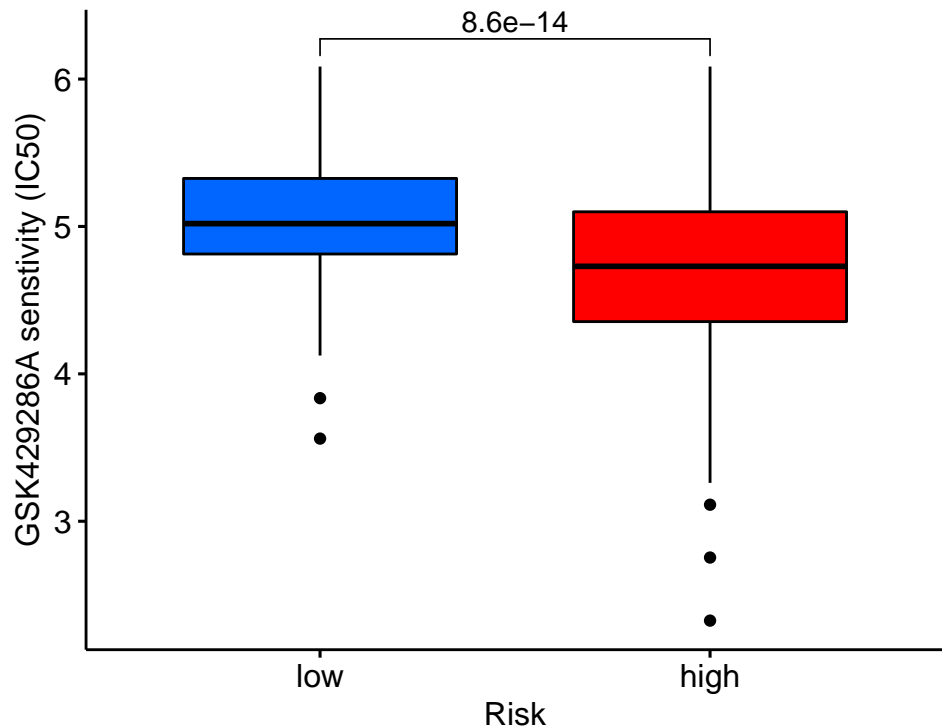

Supplement: Supplementary data 5 [file mmc5.zip › durgSenstivity.GSK429286A.pdf]

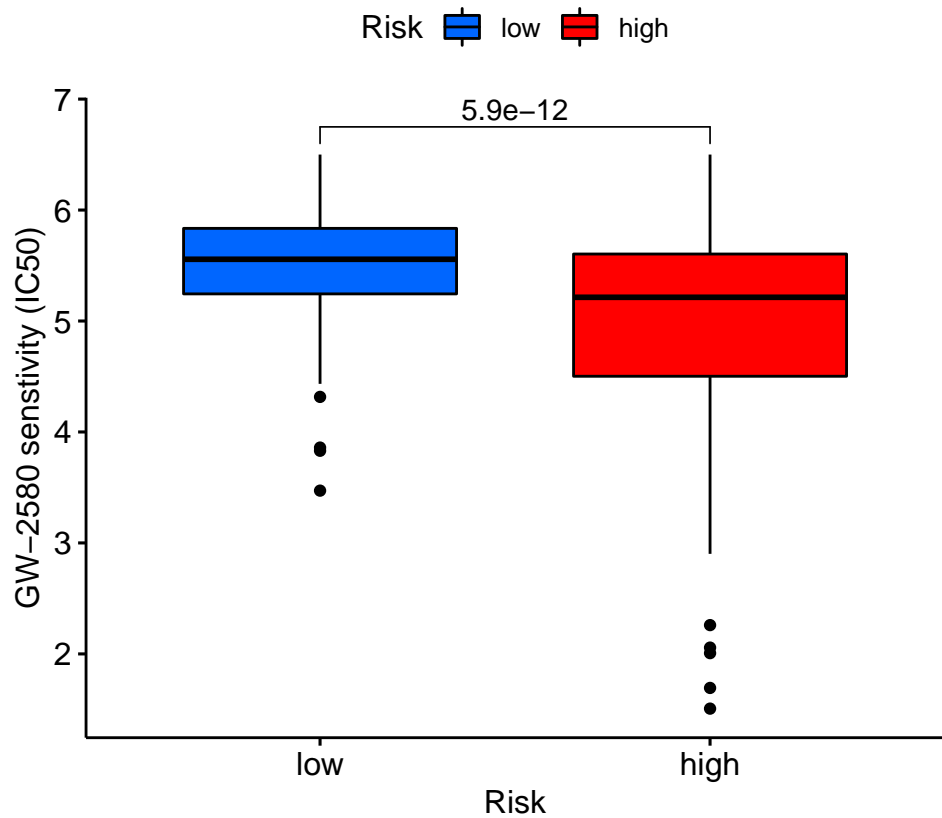

Supplement: Supplementary data 5 [file mmc5.zip › durgSenstivity.GW-2580.pdf]

Risk 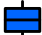 low 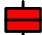 high

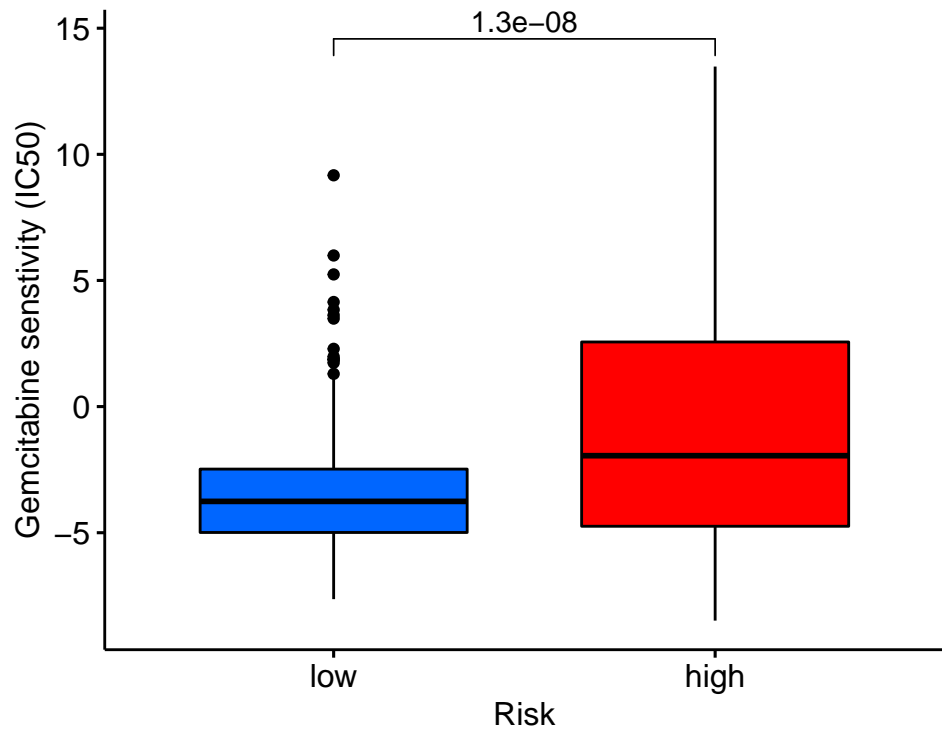

Supplement: Supplementary data 5 [file mmc5.zip › durgSenstivity.Gemcitabine.pdf]

Risk 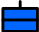 low 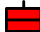 high

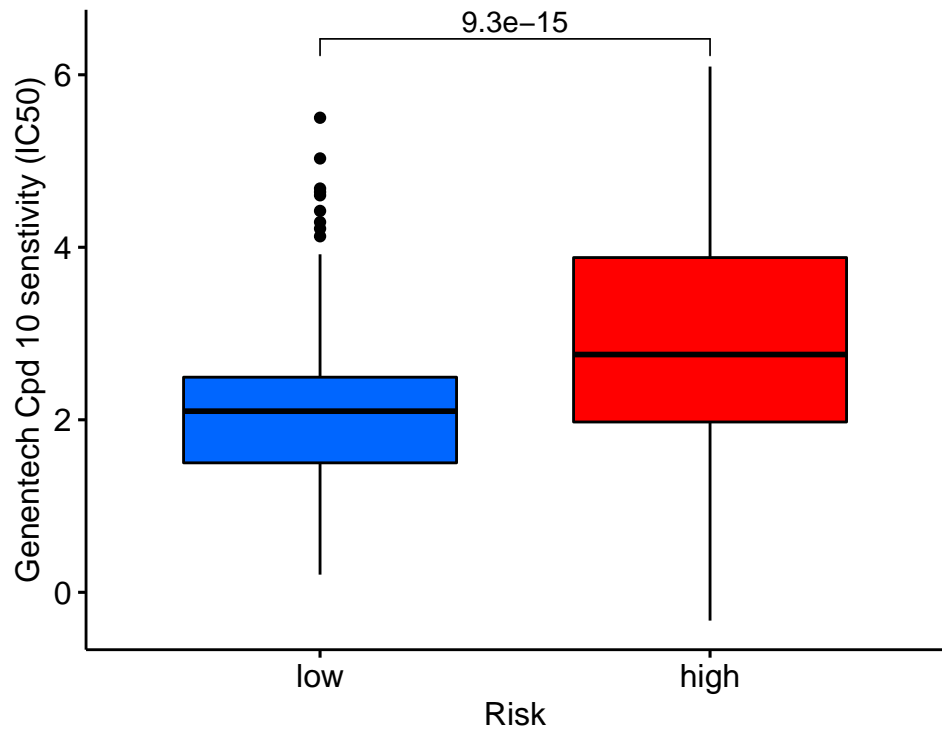

Supplement: Supplementary data 5 [file mmc5.zip › durgSenstivity.Genentech Cpd 10.pdf]

Risk 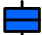 low 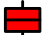 high

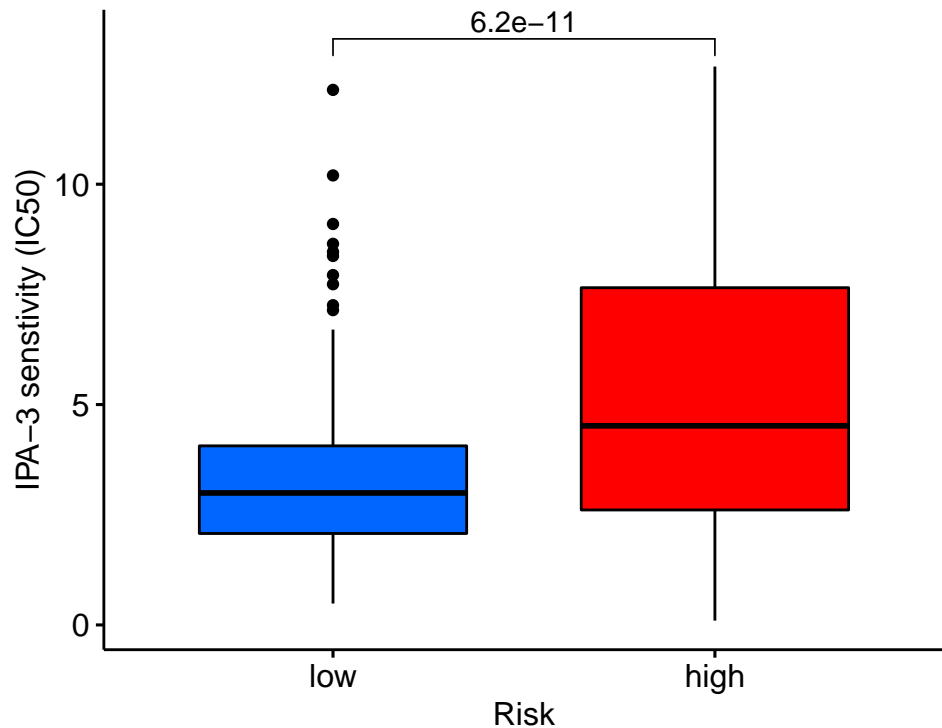

Supplement: Supplementary data 5 [file mmc5.zip › durgSenstivity.IPA-3.pdf]

Risk 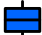 low 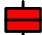 high

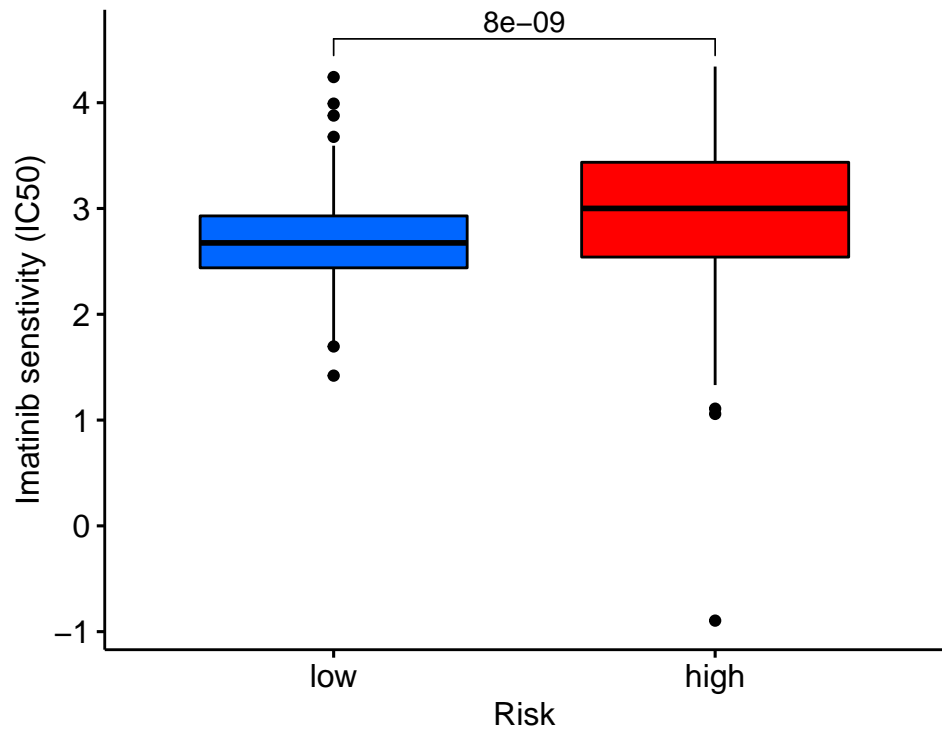

Supplement: Supplementary data 5 [file mmc5.zip › durgSenstivity.Imatinib.pdf]

Risk 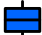 low 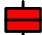 high

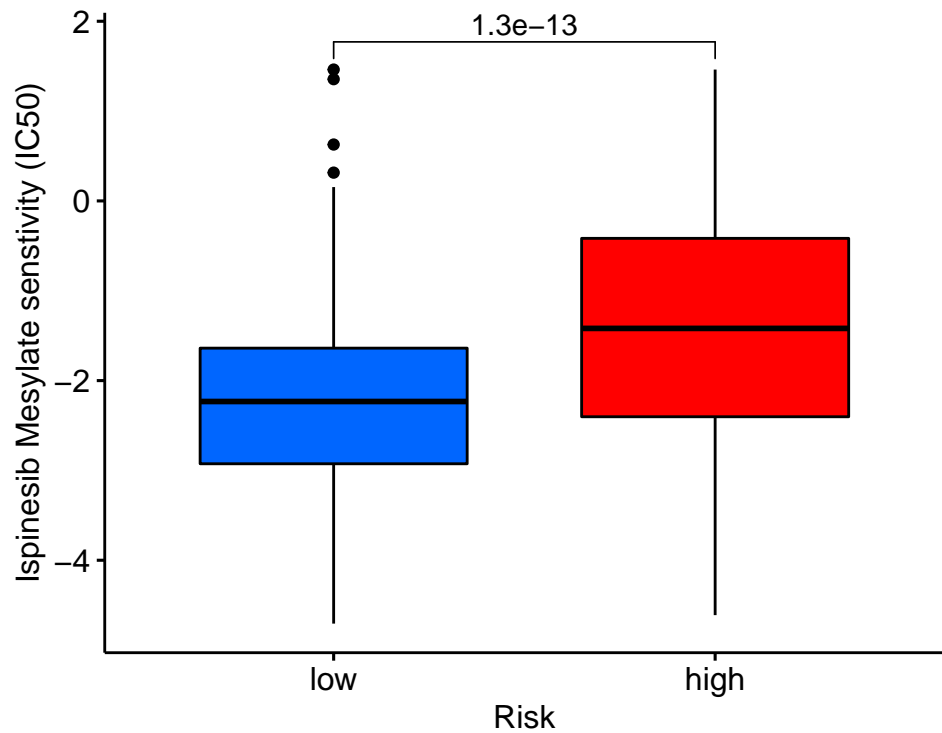

Supplement: Supplementary data 5 [file mmc5.zip › durgSenstivity.Ispinesib Mesylate.pdf]

Risk 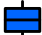 low 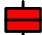 high

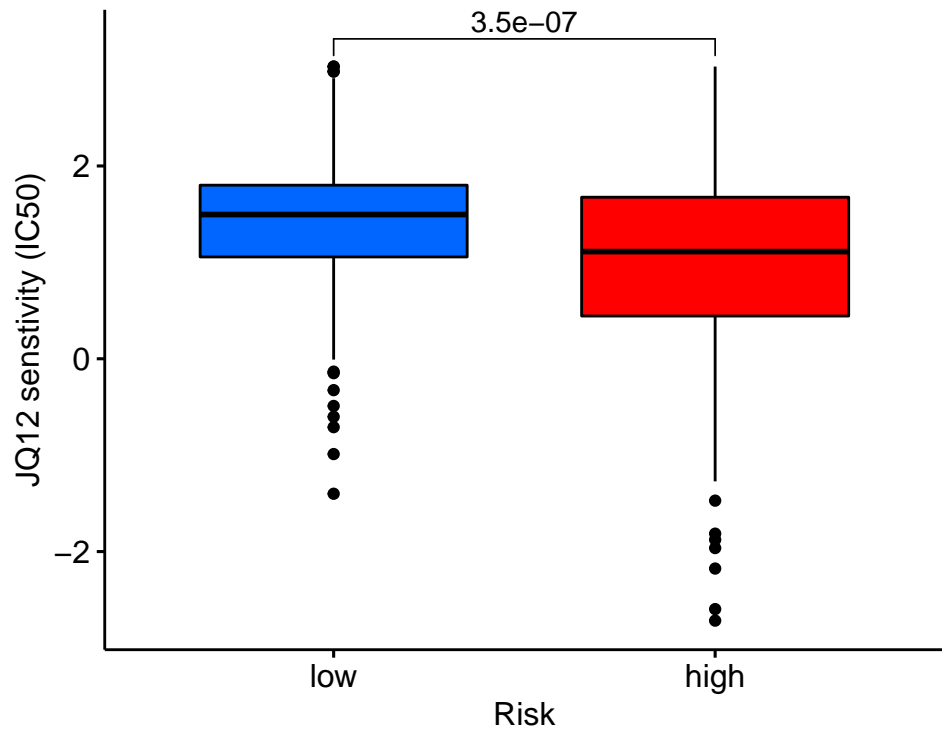

Supplement: Supplementary data 5 [file mmc5.zip › durgSenstivity.JQ12.pdf]

Risk 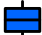 low 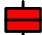 high

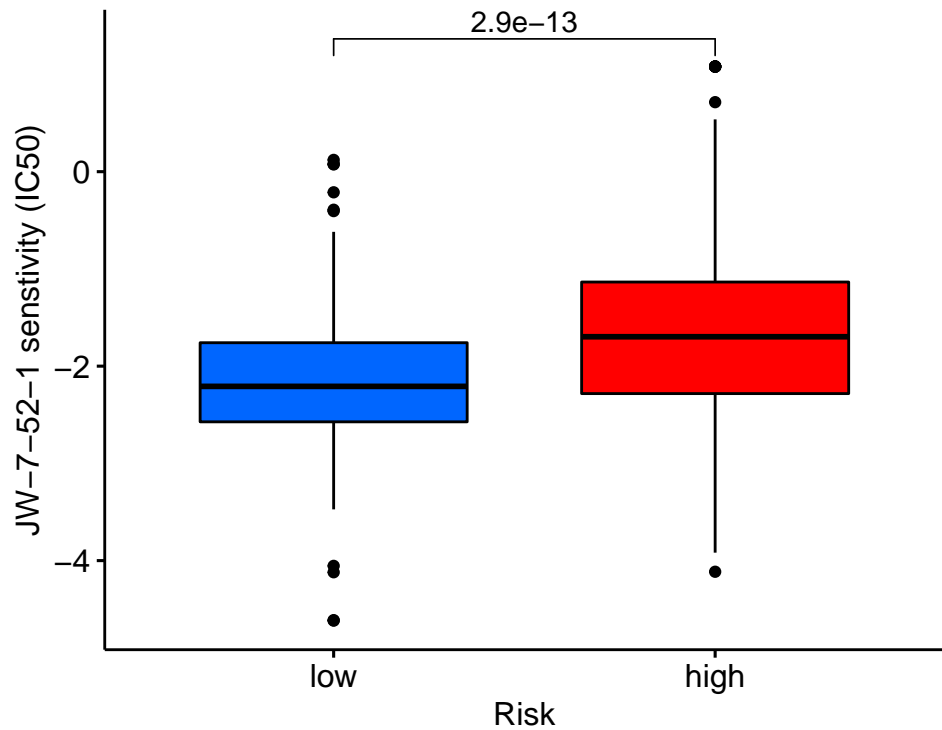

Supplement: Supplementary data 5 [file mmc5.zip › durgSenstivity.JW-7-52-1.pdf]

Risk 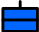 low 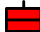 high

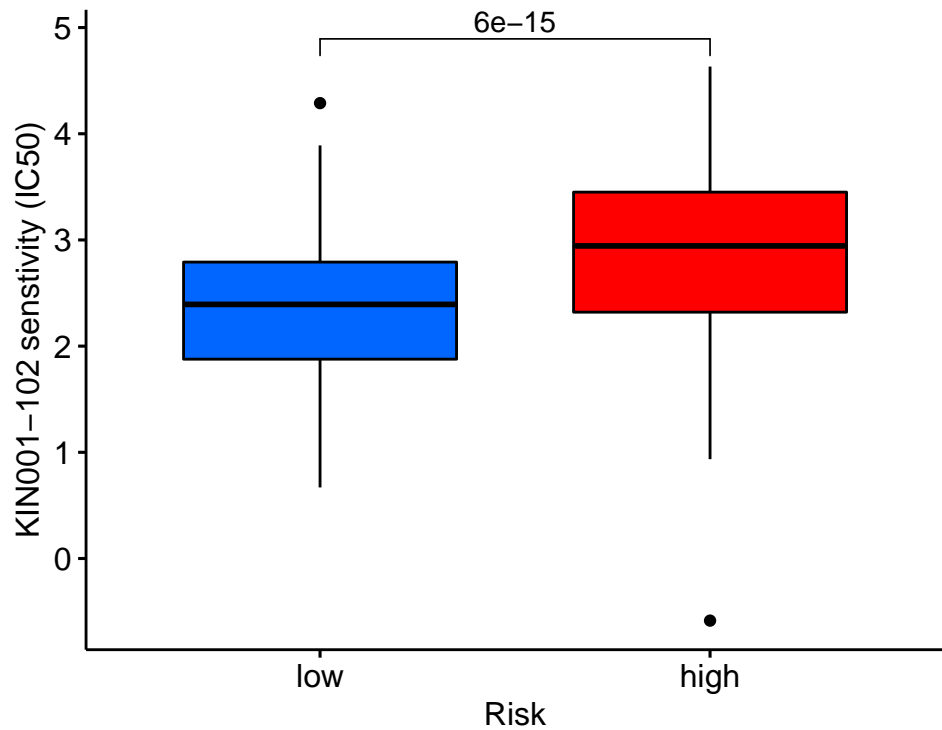

Supplement: Supplementary data 5 [file mmc5.zip › durgSenstivity.KIN001-102.pdf]

Risk 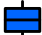 low 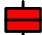 high

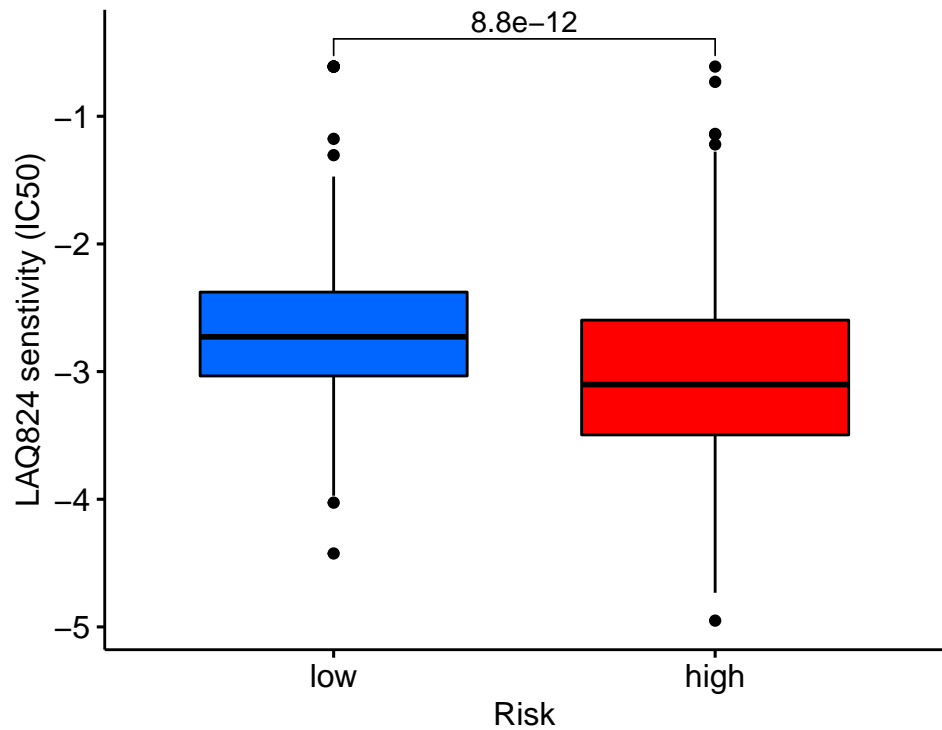

Supplement: Supplementary data 5 [file mmc5.zip › durgSenstivity.LAQ824.pdf]

Risk 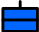 low 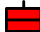 high

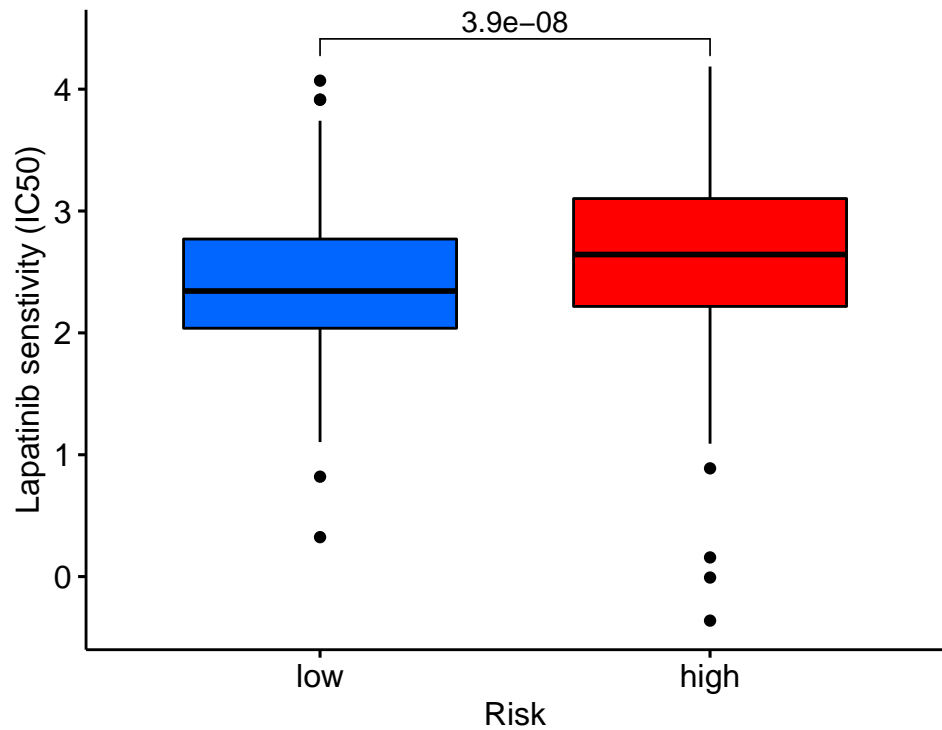

Supplement: Supplementary data 5 [file mmc5.zip › durgSenstivity.Lapatinib.pdf]

Risk 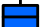 low 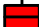 high

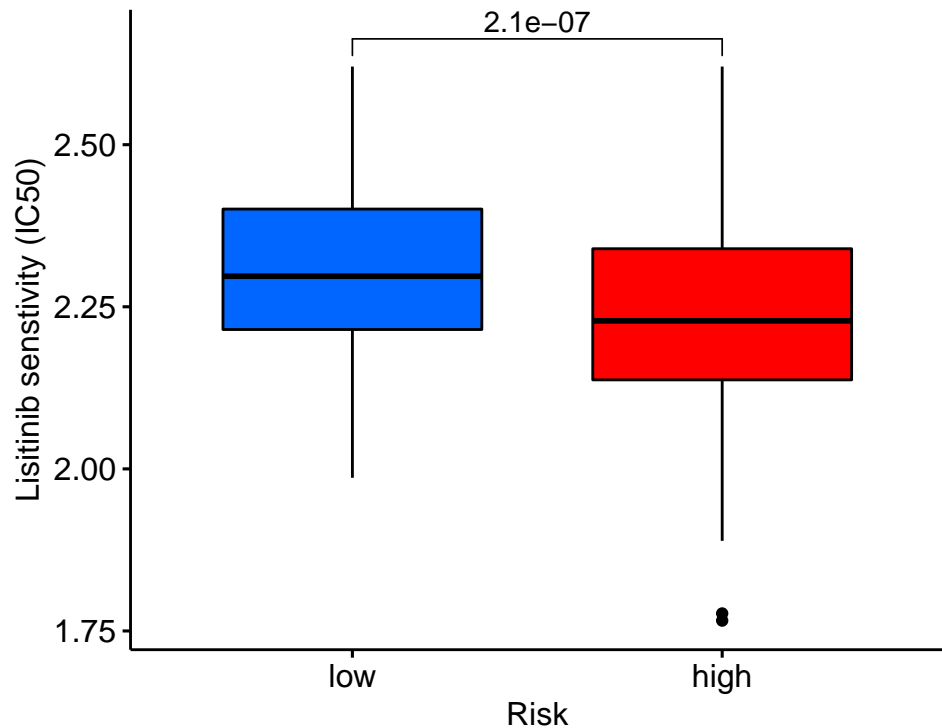

Supplement: Supplementary data 5 [file mmc5.zip › durgSenstivity.Lisitinib.pdf]

Risk 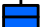 low 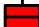 high

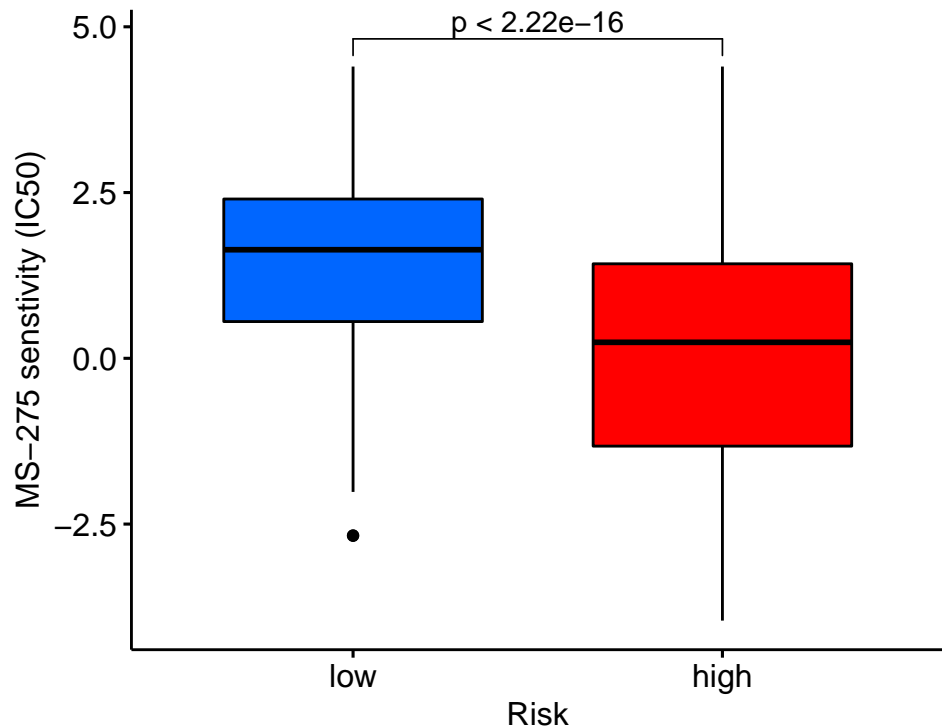

Supplement: Supplementary data 5 [file mmc5.zip › durgSenstivity.MS-275.pdf]

Risk 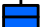 low 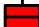 high

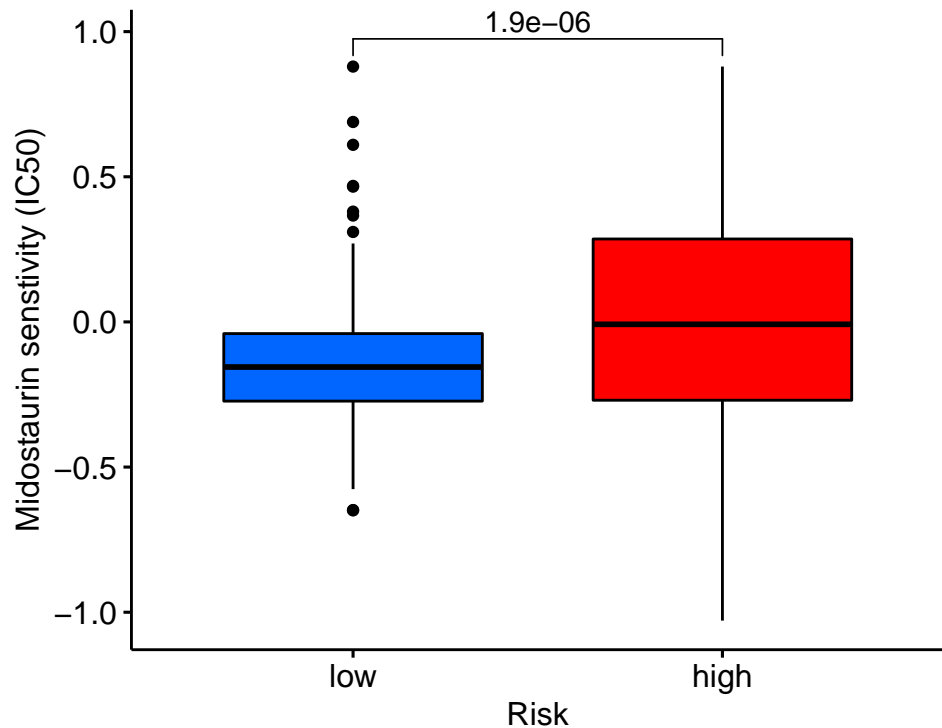

Supplement: Supplementary data 5 [file mmc5.zip › durgSenstivity.Midostaurin.pdf]

Risk 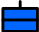 low 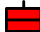 high

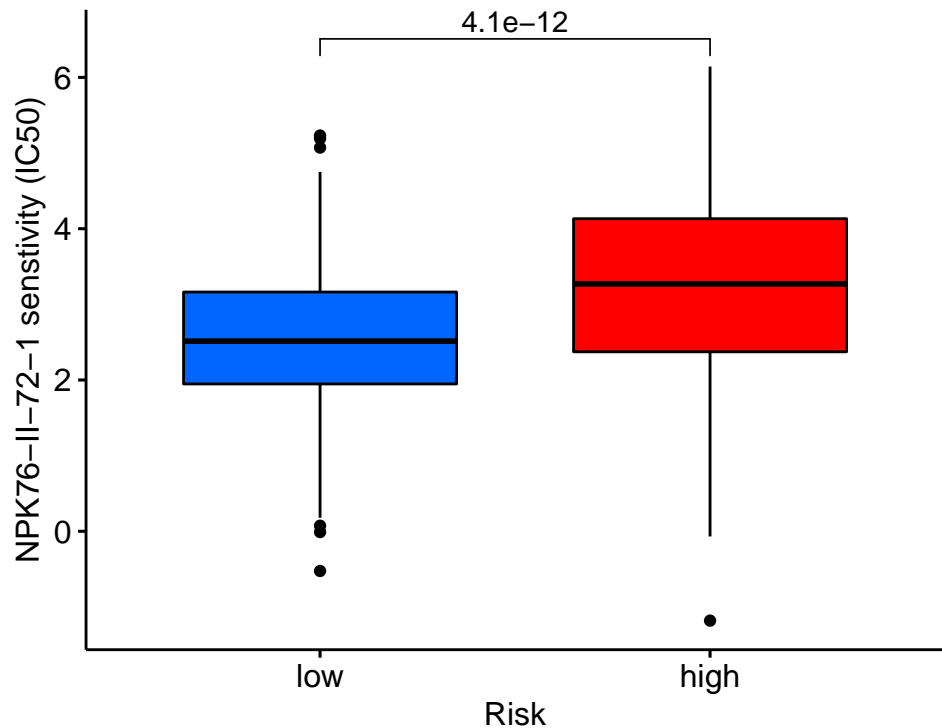

Supplement: Supplementary data 5 [file mmc5.zip › durgSenstivity.NPK76-II-72-1.pdf]

Risk 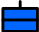 low 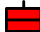 high

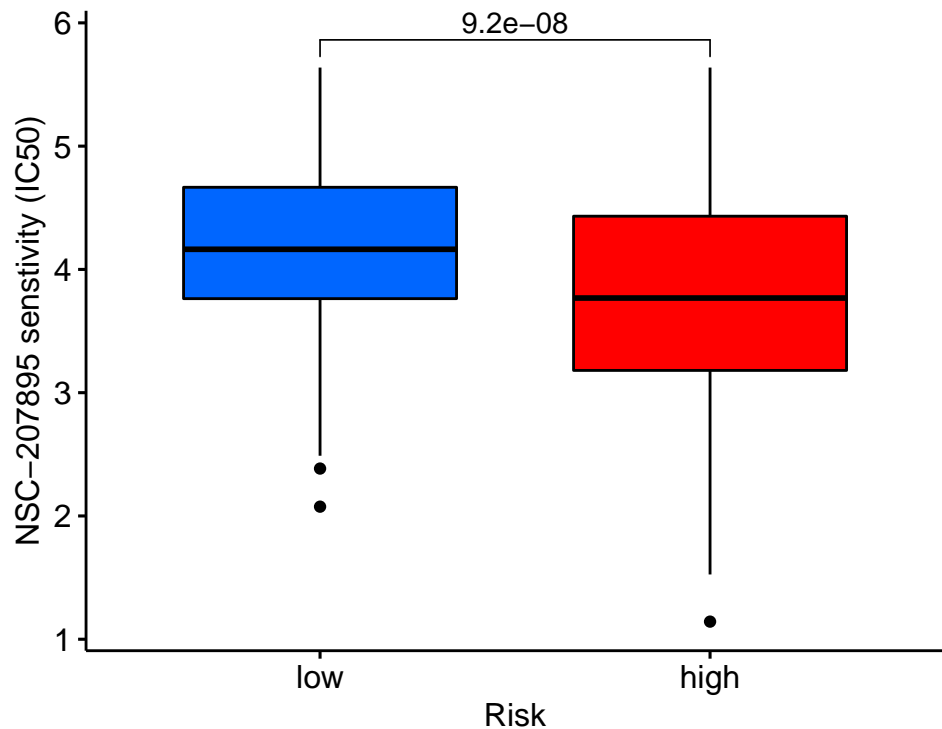

Supplement: Supplementary data 5 [file mmc5.zip › durgSenstivity.NSC-207895.pdf]

Risk 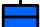 low 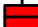 high

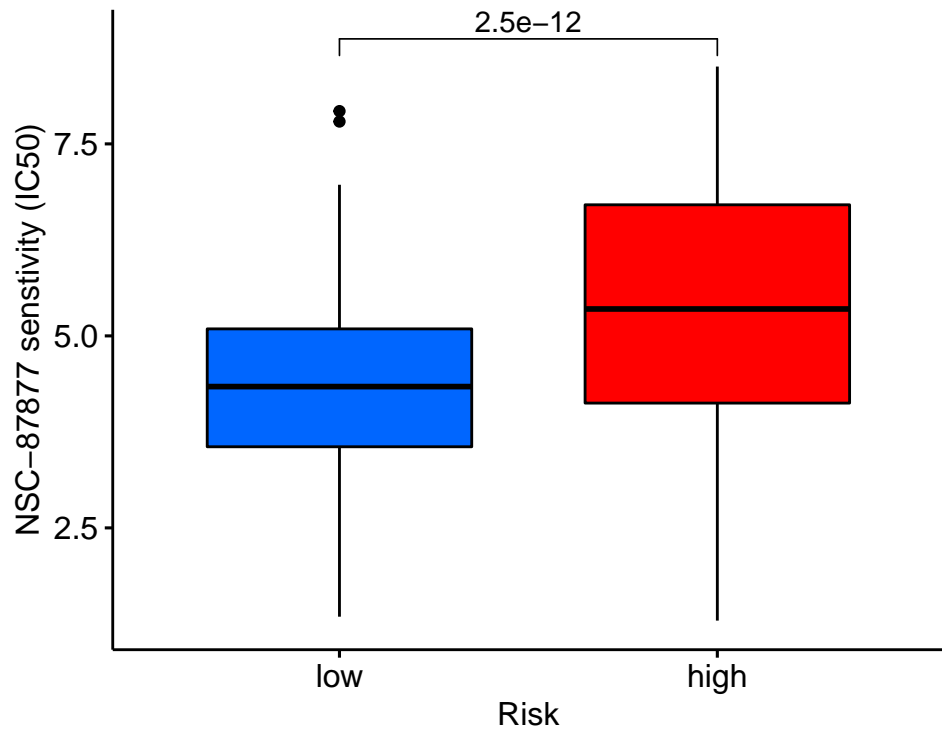

Supplement: Supplementary data 5 [file mmc5.zip › durgSenstivity.NSC-87877.pdf]

Risk 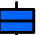 low 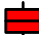 high

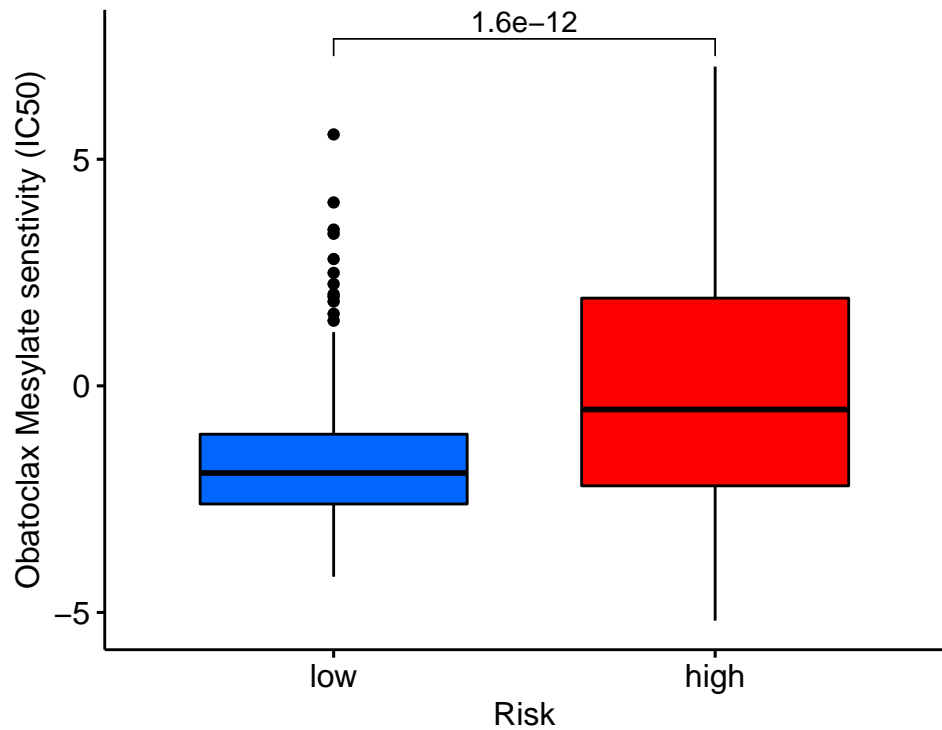

Supplement: Supplementary data 5 [file mmc5.zip › durgSenstivity.Obatoclax Mesylate.pdf]

Risk 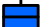 low 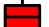 high

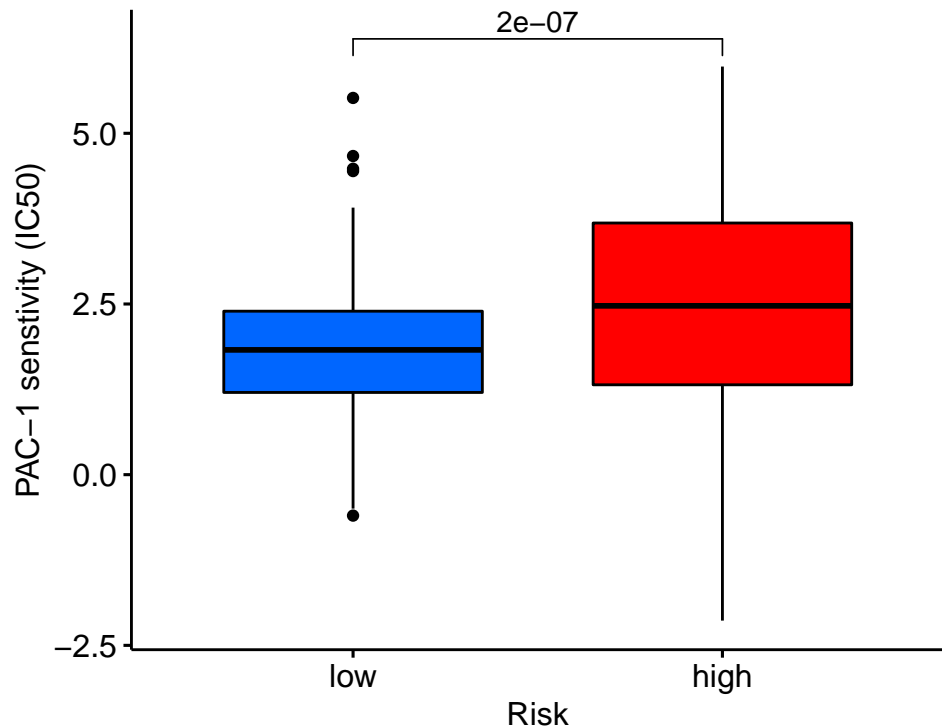

Supplement: Supplementary data 5 [file mmc5.zip › durgSenstivity.PAC-1.pdf]

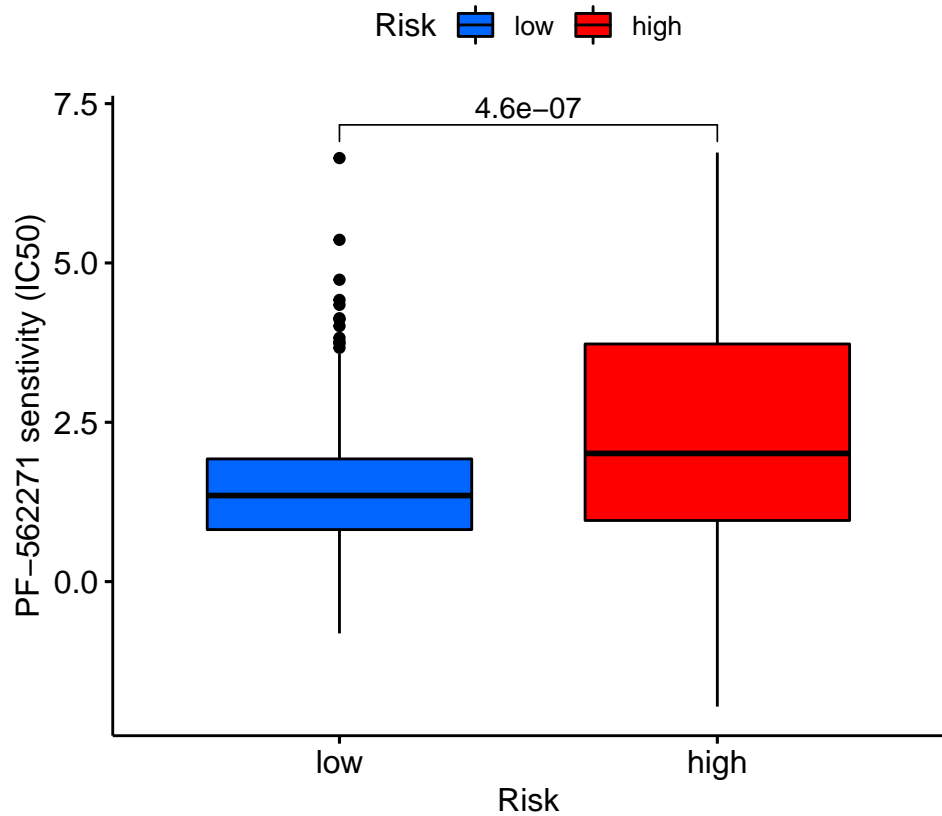

Supplement: Supplementary data 5 [file mmc5.zip › durgSenstivity.PF-562271.pdf]

Risk 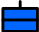 low 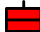 high

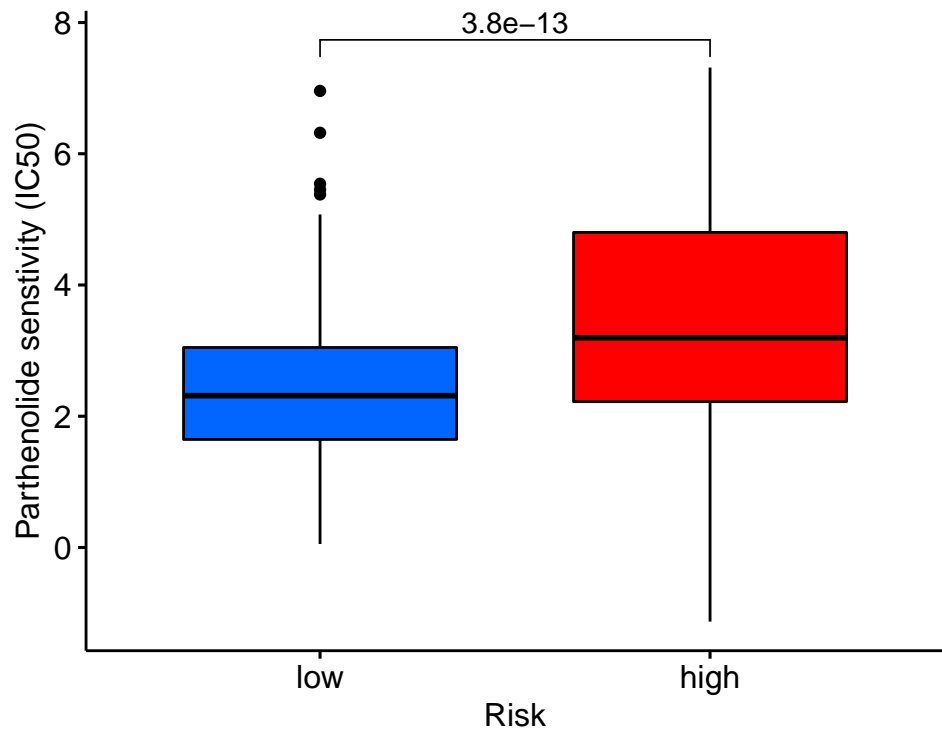

Supplement: Supplementary data 5 [file mmc5.zip › durgSenstivity.Parthenolide.pdf]

Risk 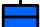 low 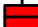 high

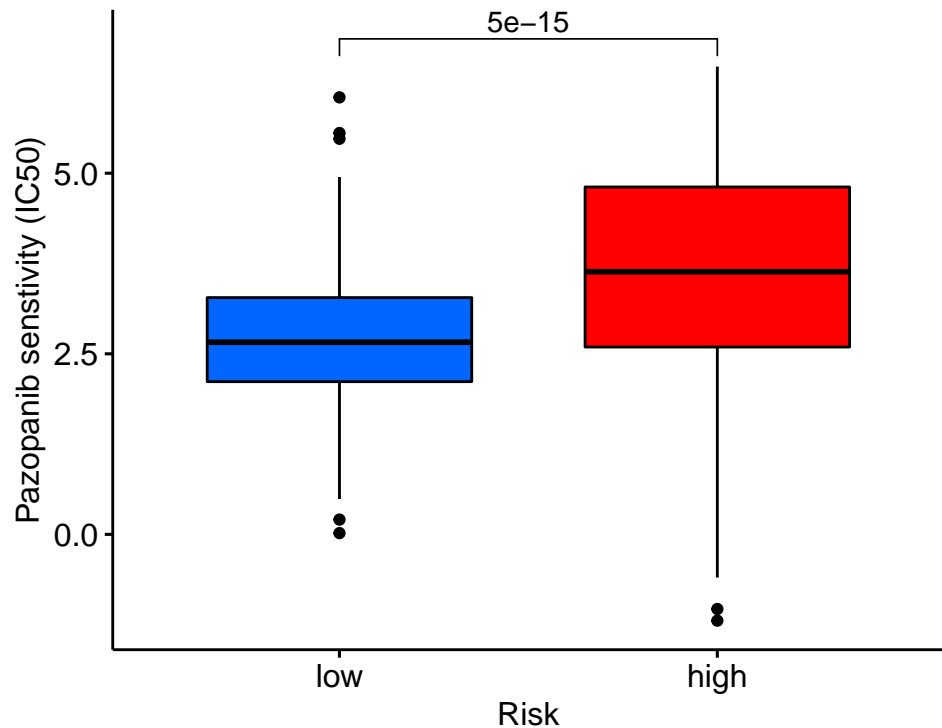

Supplement: Supplementary data 5 [file mmc5.zip › durgSenstivity.Pazopanib.pdf]

Risk 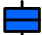 low 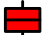 high

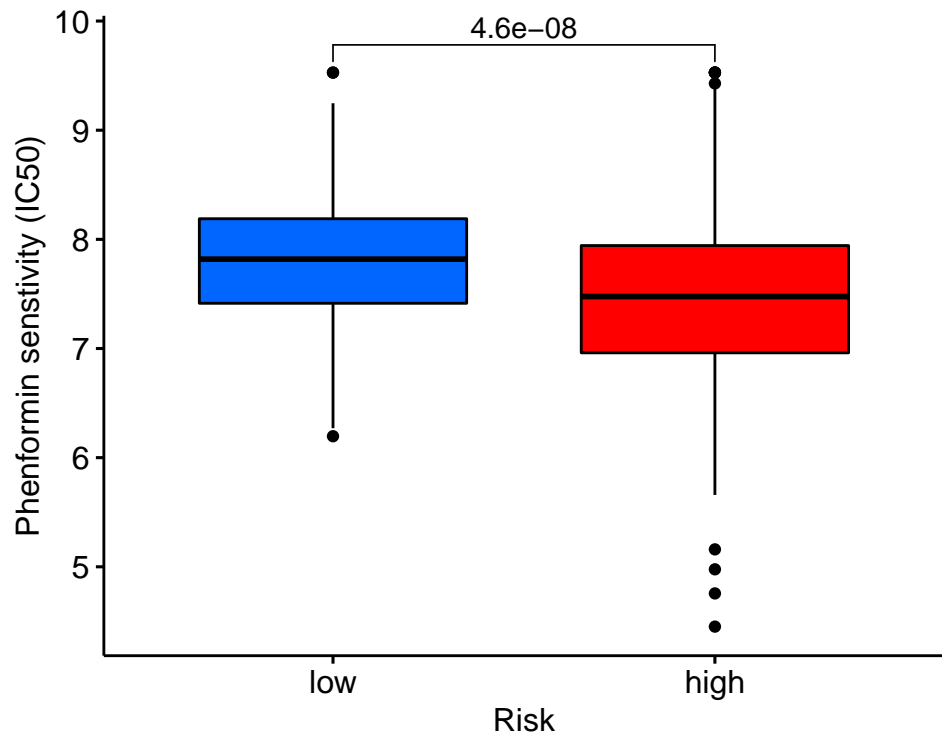

Supplement: Supplementary data 5 [file mmc5.zip › durgSenstivity.Phenformin.pdf]

Risk 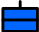 low 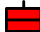 high

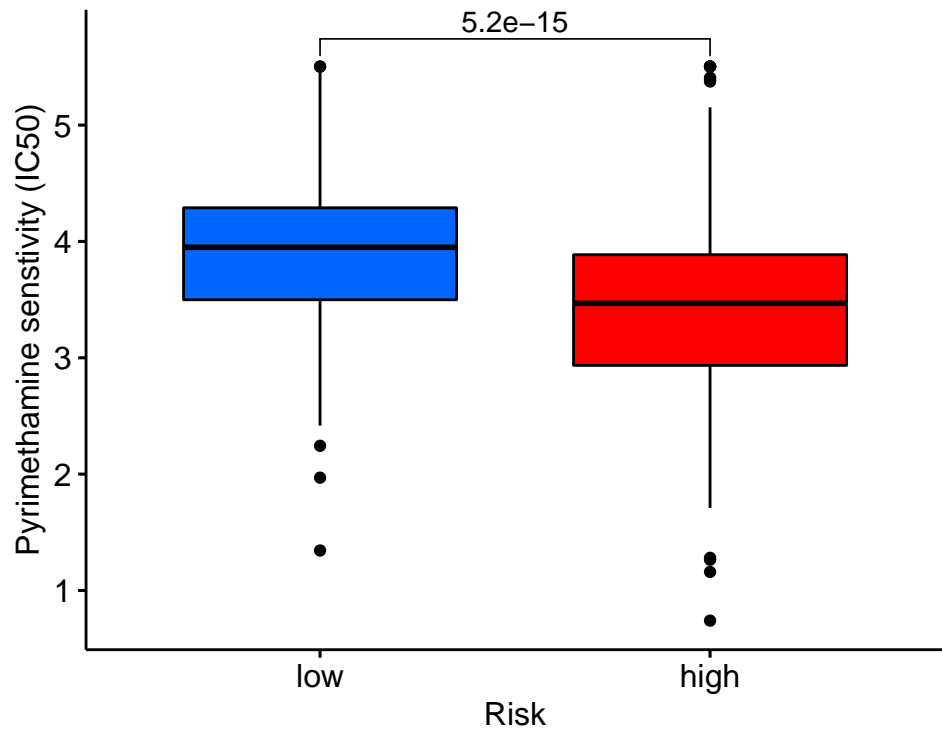

Supplement: Supplementary data 5 [file mmc5.zip › durgSenstivity.Pyrimethamine.pdf]

Risk 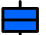 low 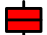 high

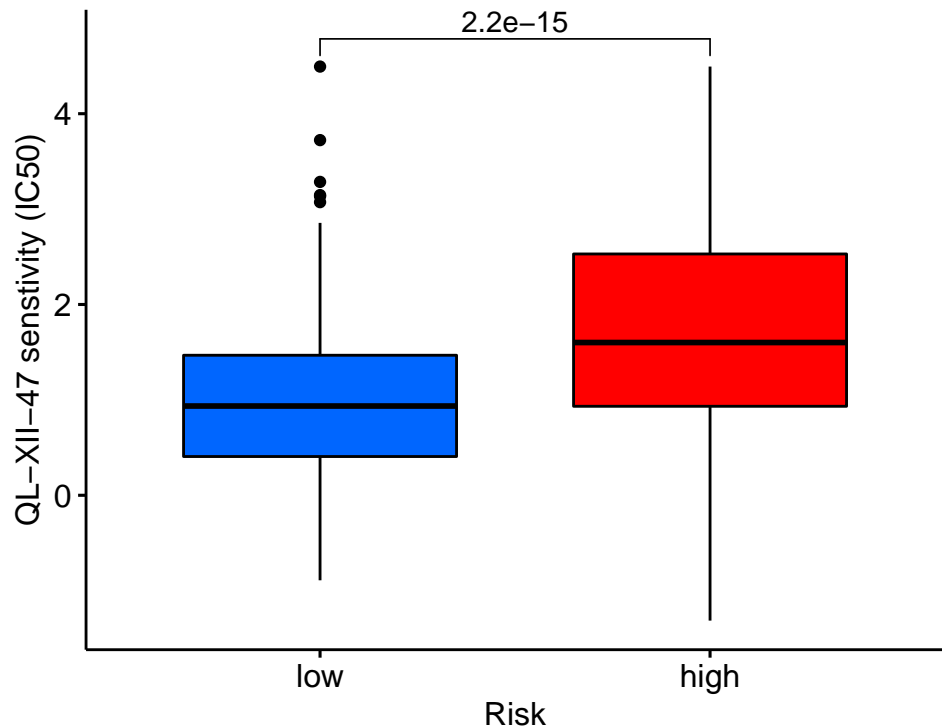

Supplement: Supplementary data 5 [file mmc5.zip › durgSenstivity.QL-XII-47.pdf]

Risk 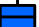 low 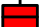 high

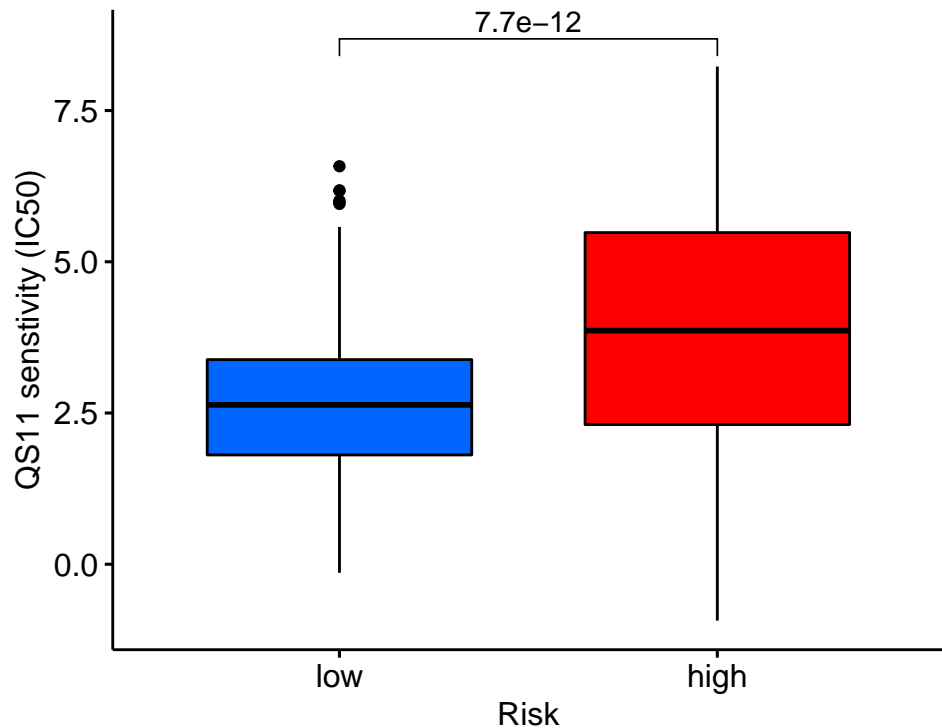

Supplement: Supplementary data 5 [file mmc5.zip › durgSenstivity.QS11.pdf]

Risk 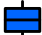 low 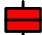 high

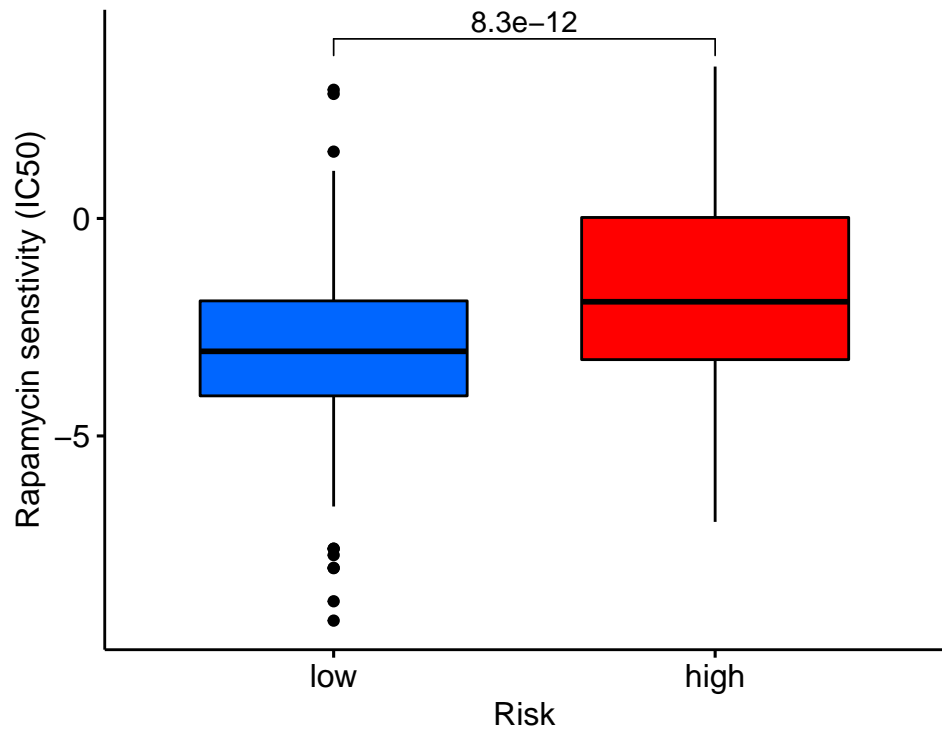

Supplement: Supplementary data 5 [file mmc5.zip › durgSenstivity.Rapamycin.pdf]

Risk 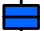 low 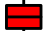 high

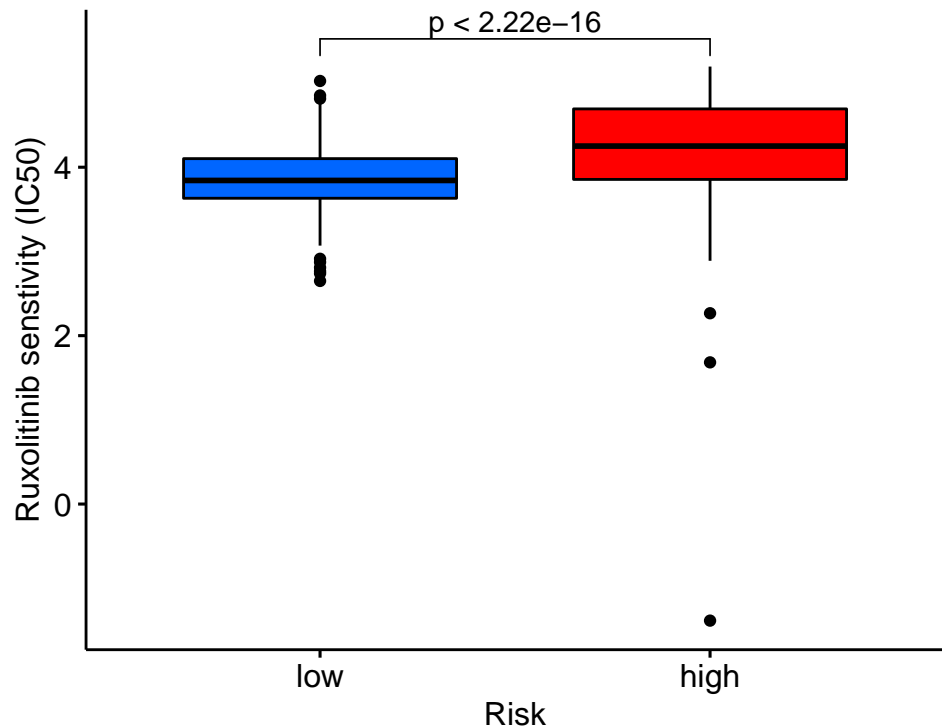

Supplement: Supplementary data 5 [file mmc5.zip › durgSenstivity.Ruxolitinib.pdf]

Risk 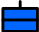 low 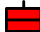 high

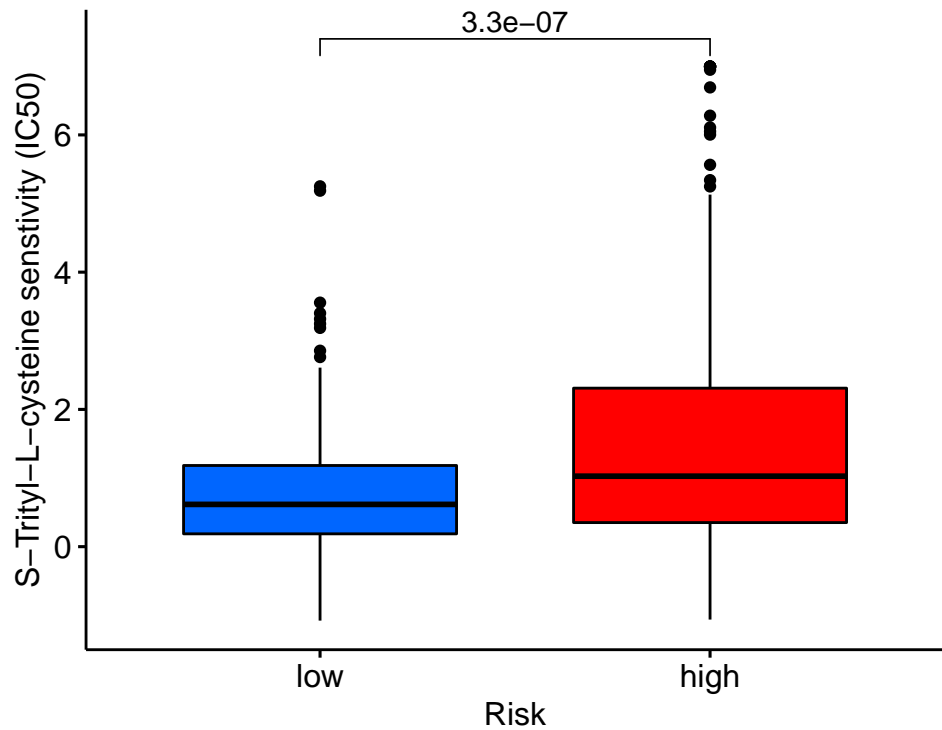

Supplement: Supplementary data 5 [file mmc5.zip › durgSenstivity.S-Trityl-L-cysteine.pdf]

Risk 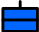 low 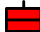 high

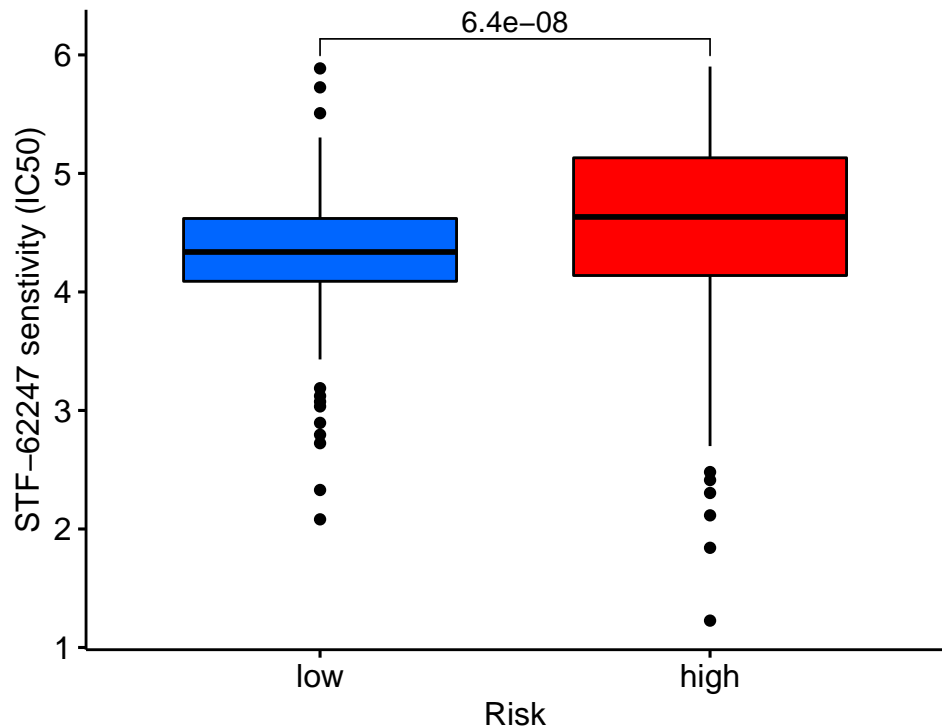

Supplement: Supplementary data 5 [file mmc5.zip › durgSenstivity.STF-62247.pdf]

Risk 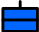 low 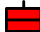 high

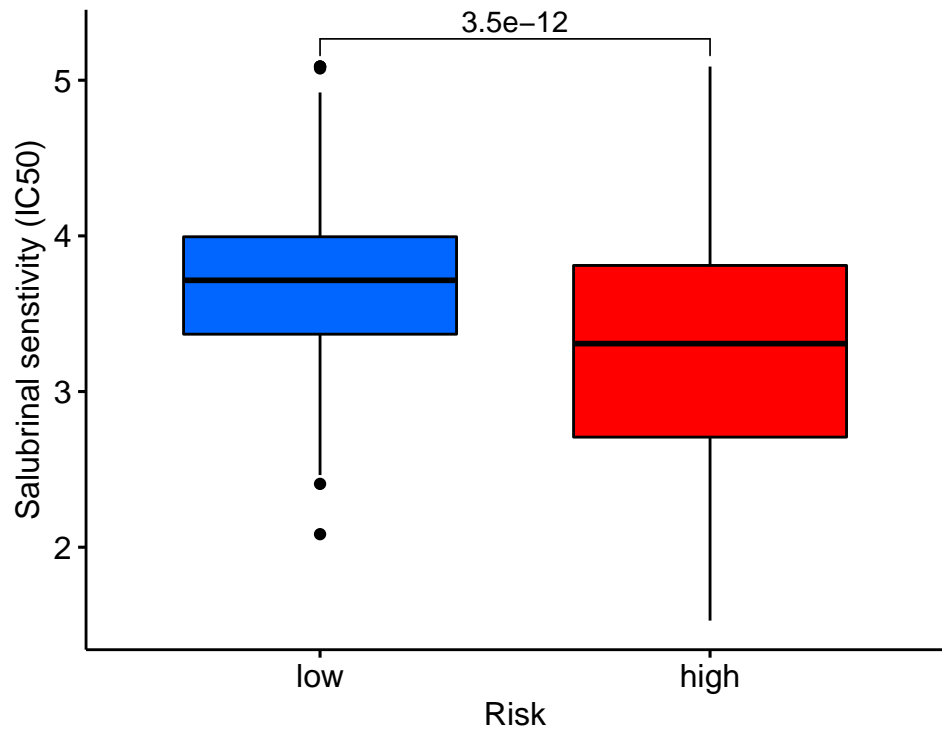

Supplement: Supplementary data 5 [file mmc5.zip › durgSenstivity.Salubrinal.pdf]

Risk 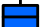 low 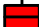 high

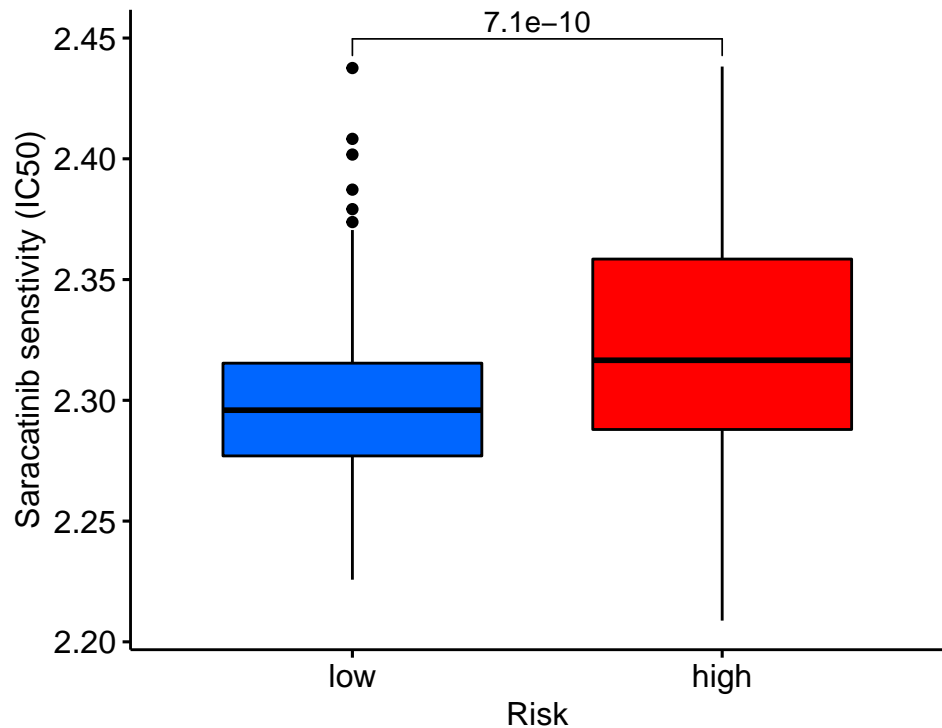

Supplement: Supplementary data 5 [file mmc5.zip › durgSenstivity.Saracatinib.pdf]

Risk 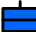 low 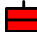 high

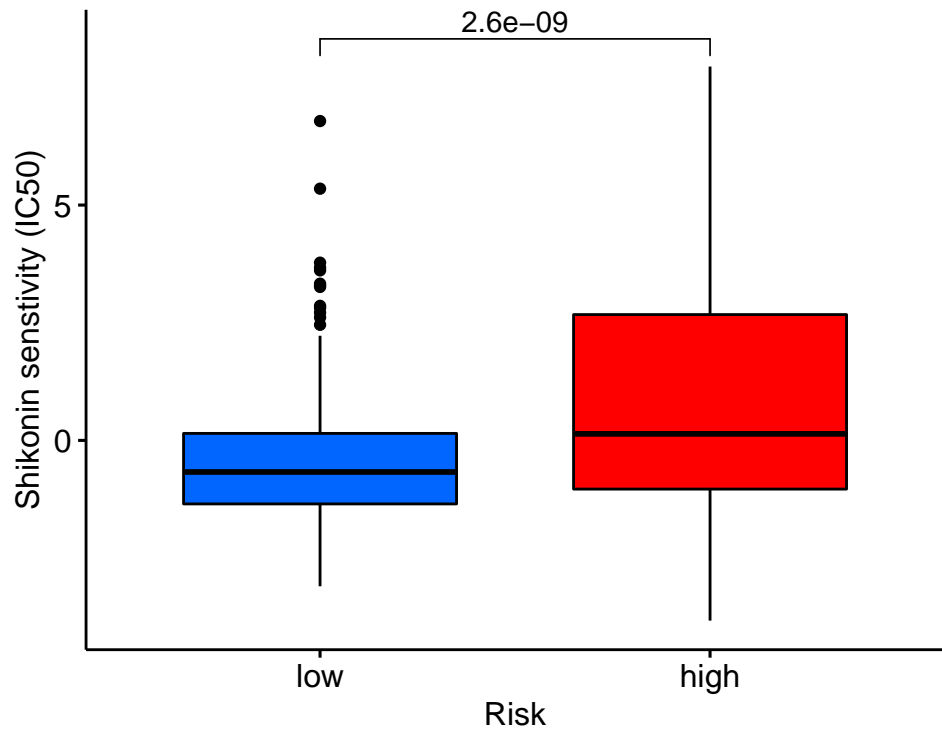

Supplement: Supplementary data 5 [file mmc5.zip › durgSenstivity.Shikonin.pdf]

Risk 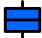 low 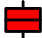 high

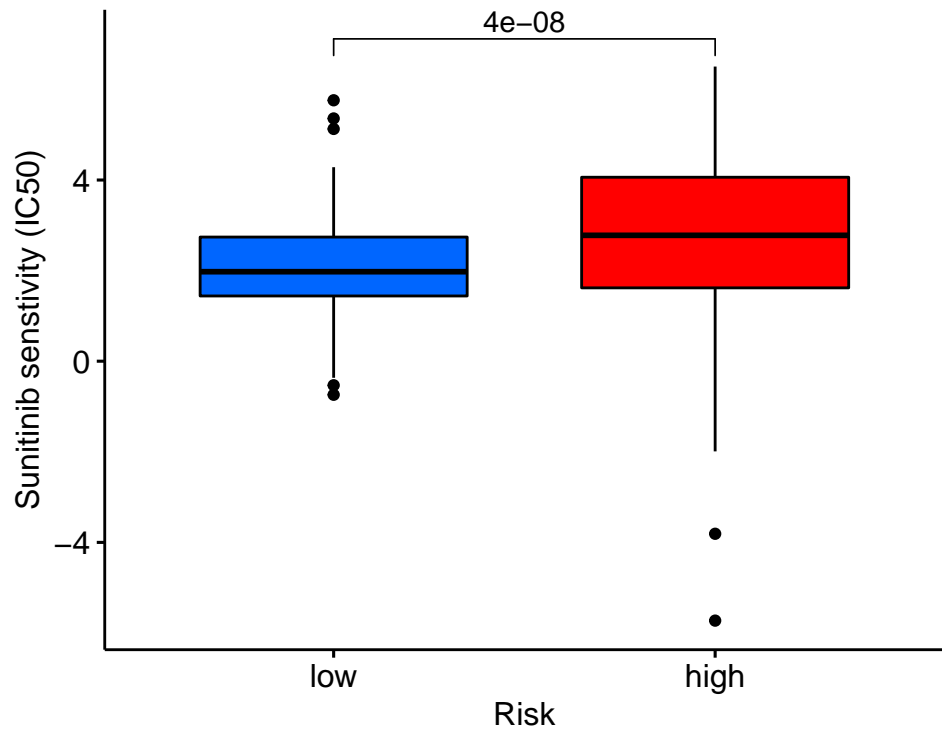

Supplement: Supplementary data 5 [file mmc5.zip › durgSenstivity.Sunitinib.pdf]

Risk 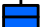 low 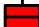 high

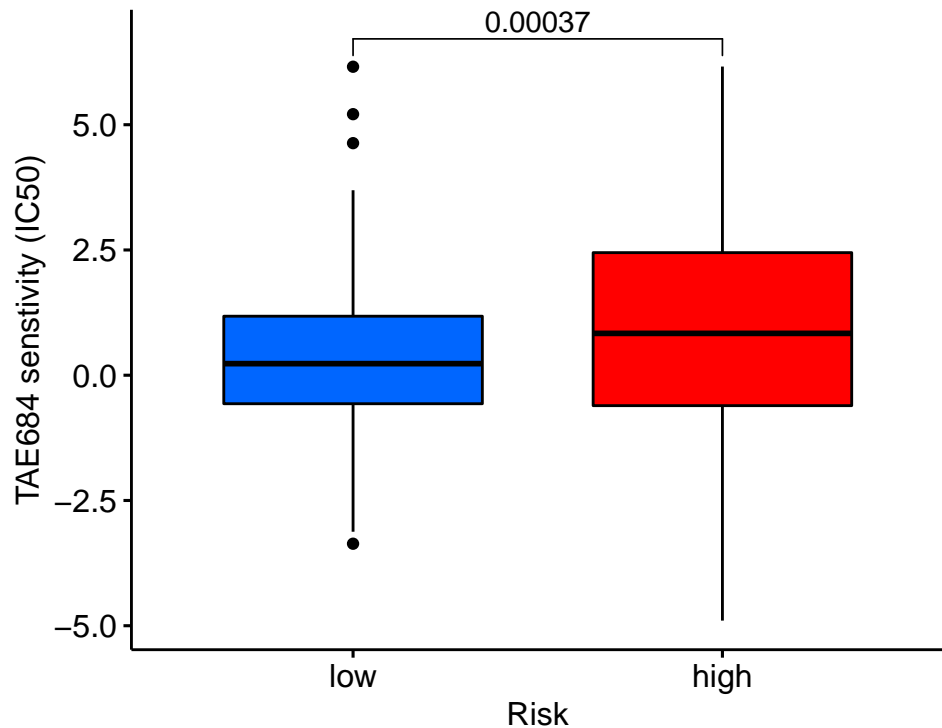

Supplement: Supplementary data 5 [file mmc5.zip › durgSenstivity.TAE684.pdf]

Risk 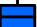 low 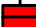 high

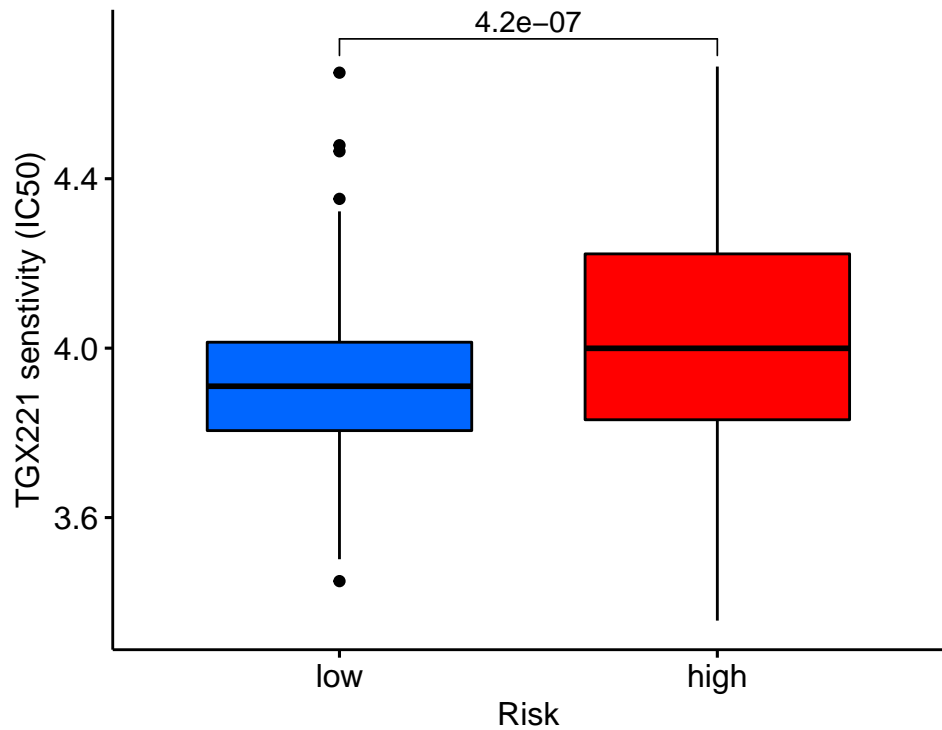

Supplement: Supplementary data 5 [file mmc5.zip › durgSenstivity.TGX221.pdf]

Risk 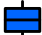 low 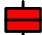 high

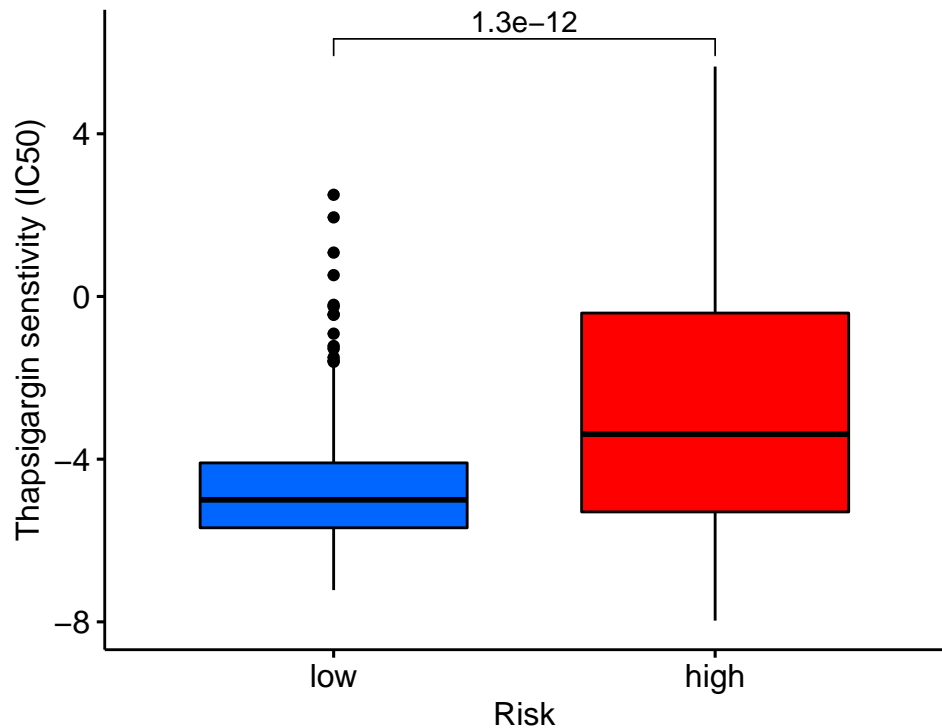

Supplement: Supplementary data 5 [file mmc5.zip › durgSenstivity.Thapsigargin.pdf]

Tipifarnib sensitivity (IC50)

Risk 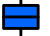 low 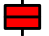 high

$1.6e-13$

2.5

0.0

-2.5

low

high

Risk

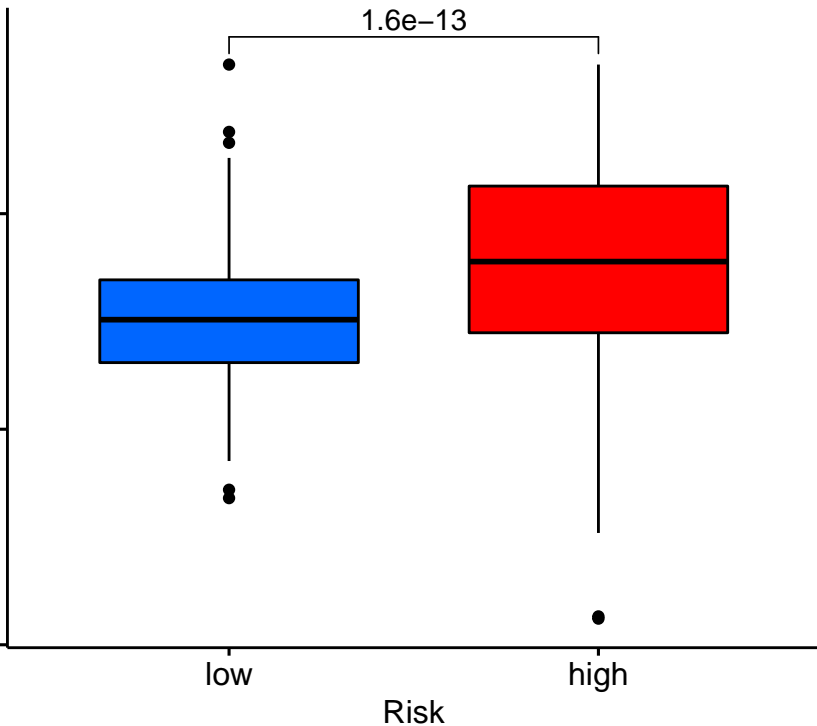

Supplement: Supplementary data 5 [file mmc5.zip › durgSenstivity.Tipifarnib.pdf]

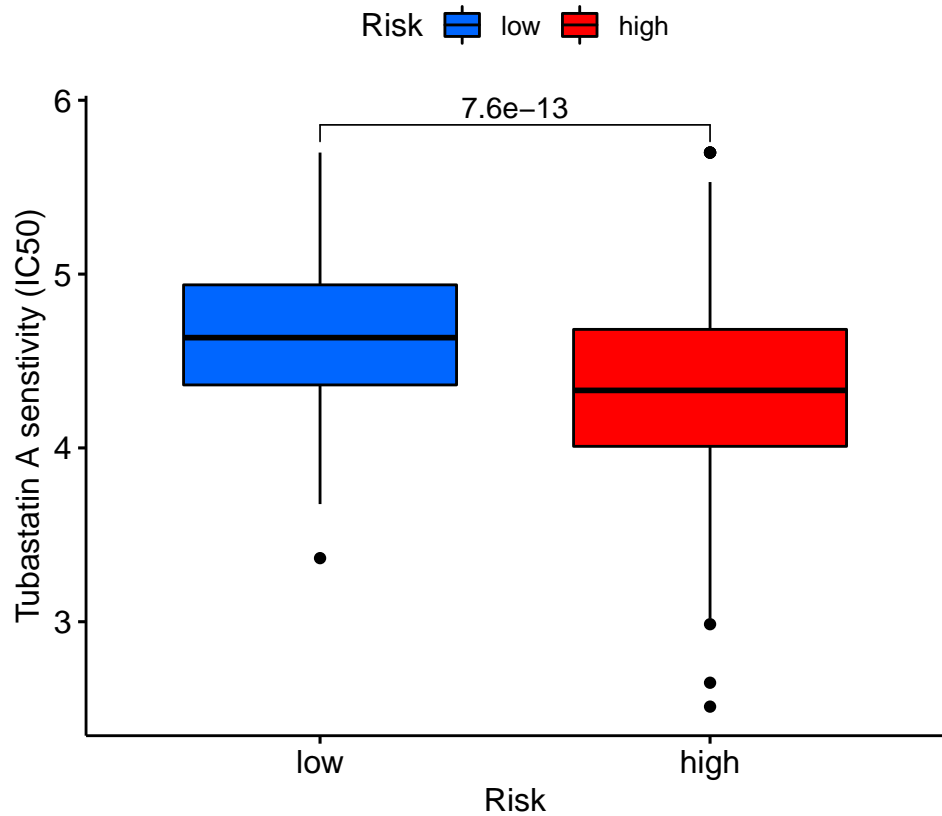

Supplement: Supplementary data 5 [file mmc5.zip › durgSenstivity.Tubastatin A.pdf]

Risk 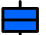 low 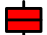 high

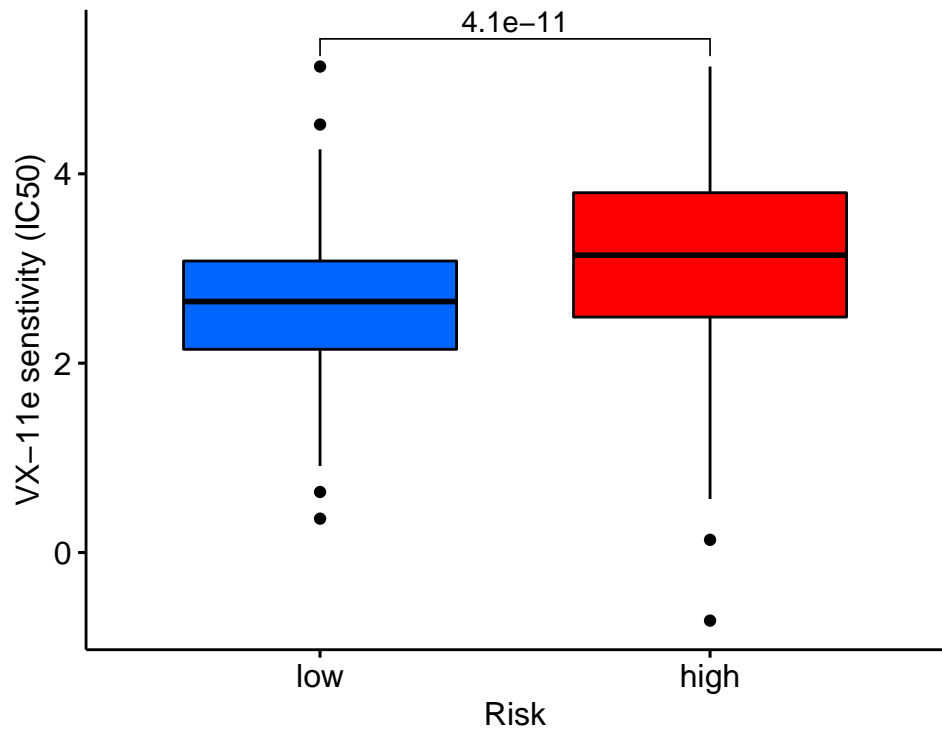

Supplement: Supplementary data 5 [file mmc5.zip › durgSenstivity.VX-11e.pdf]

Risk 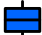 low 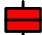 high

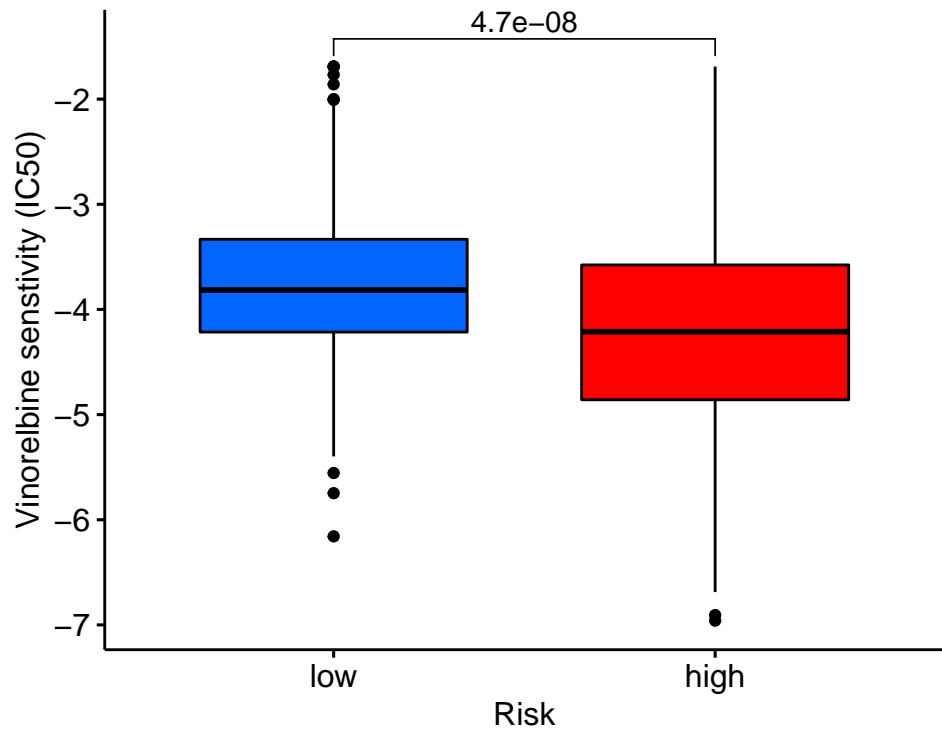

Supplement: Supplementary data 5 [file mmc5.zip › durgSenstivity.Vinorelbine.pdf]

Risk 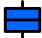 low 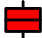 high

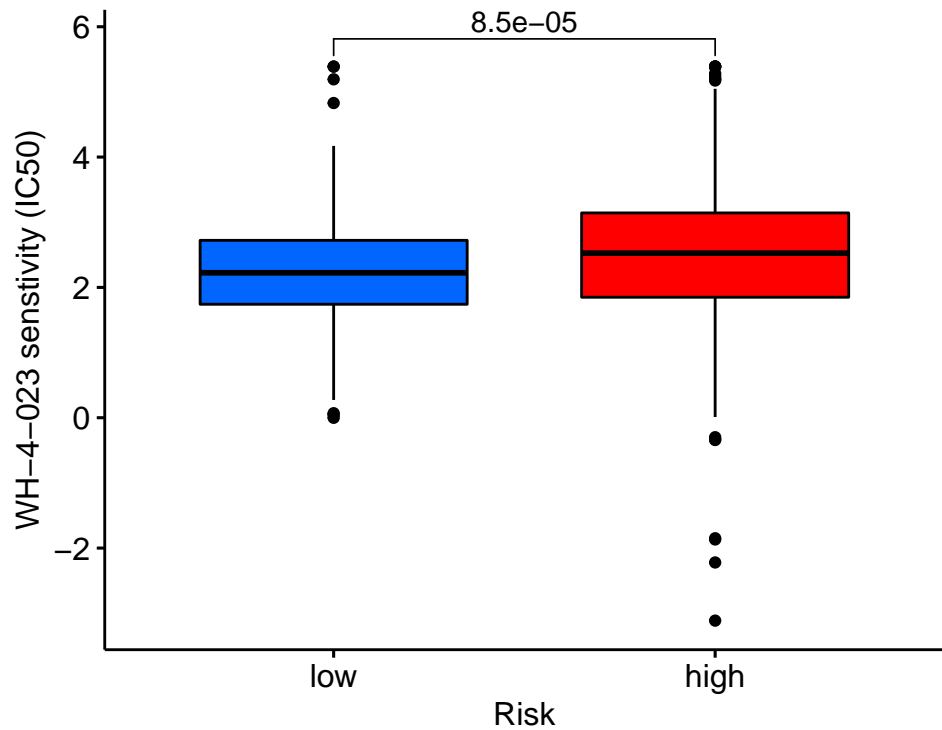

Supplement: Supplementary data 5 [file mmc5.zip › durgSenstivity.WH-4-023.pdf]

Risk 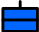 low 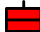 high

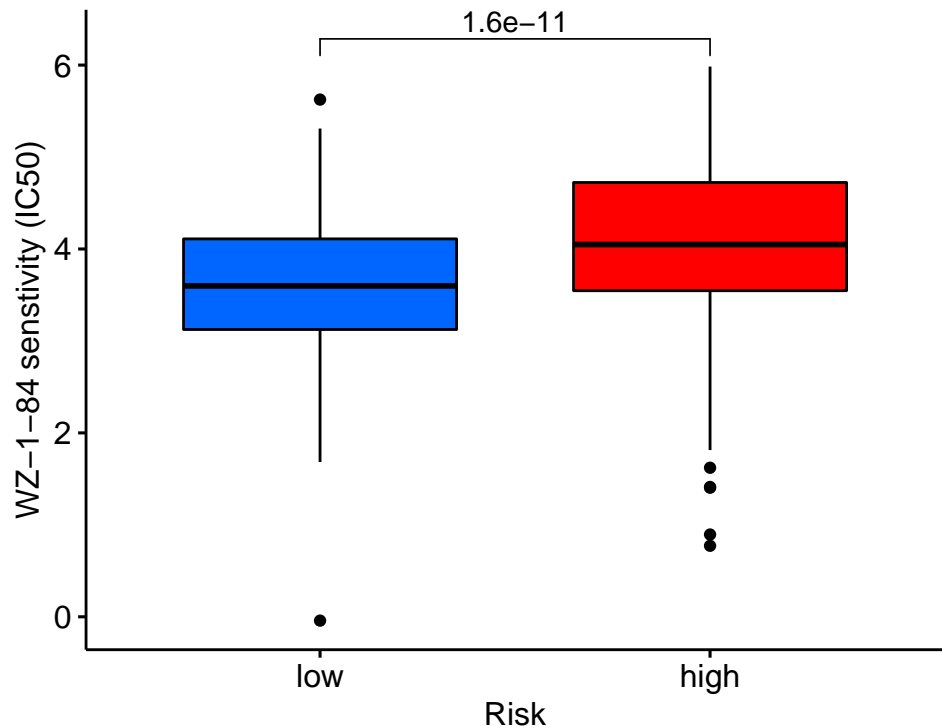

Supplement: Supplementary data 5 [file mmc5.zip › durgSenstivity.WZ-1-84.pdf]

Risk 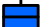 low 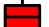 high

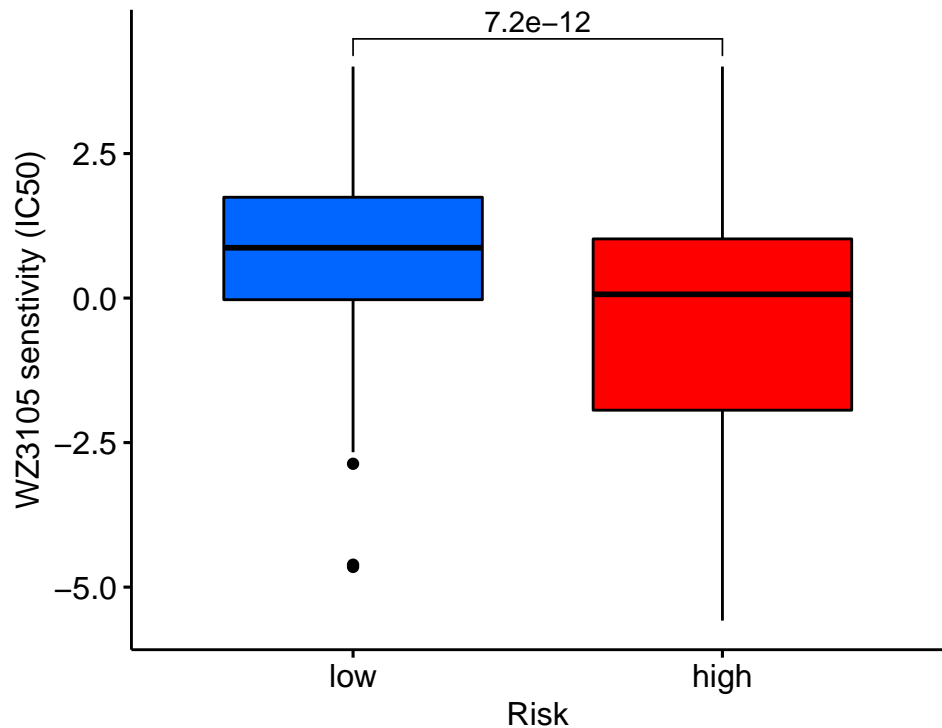

Supplement: Supplementary data 5 [file mmc5.zip › durgSenstivity.WZ3105.pdf]

Risk 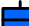 low 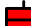 high

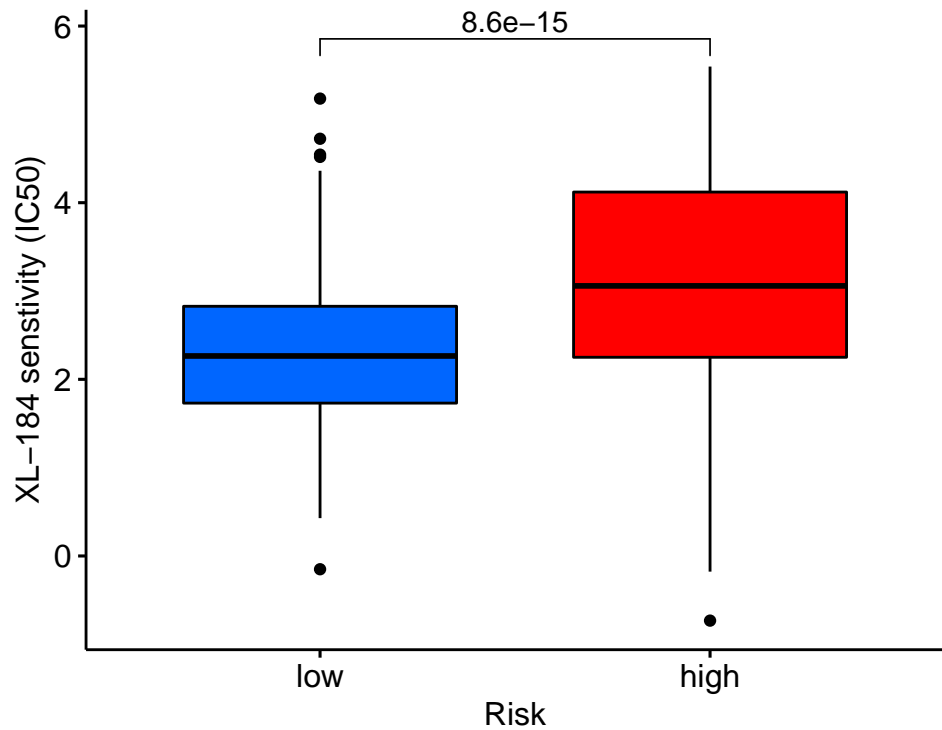

Supplement: Supplementary data 5 [file mmc5.zip › durgSenstivity.XL-184.pdf]

Risk 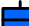 low 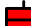 high

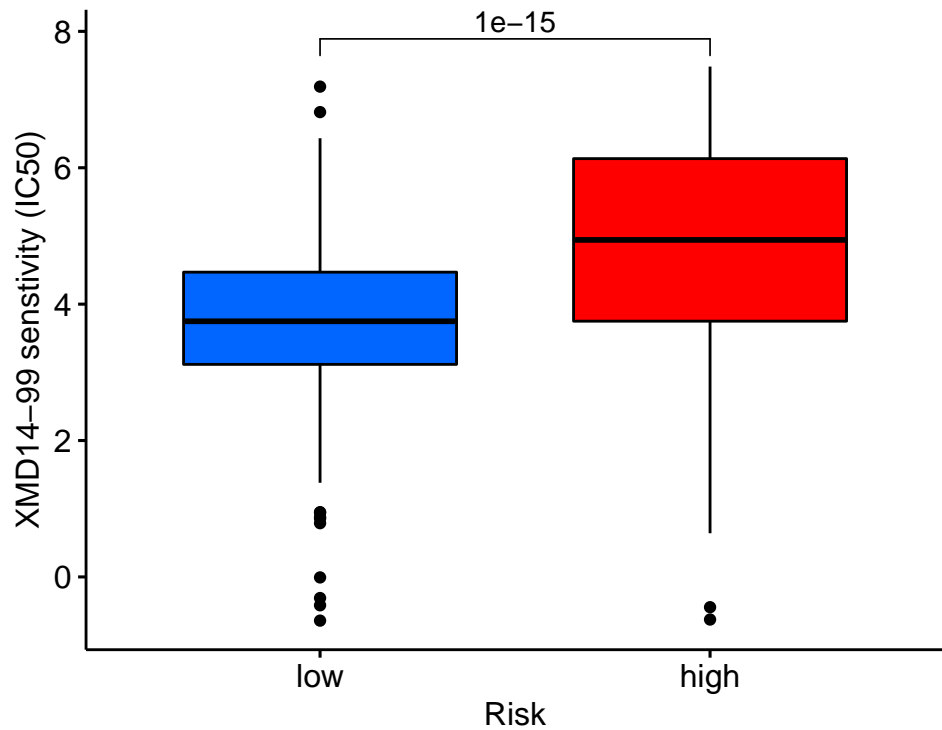

Supplement: Supplementary data 5 [file mmc5.zip › durgSenstivity.XMD14-99.pdf]

Risk low high

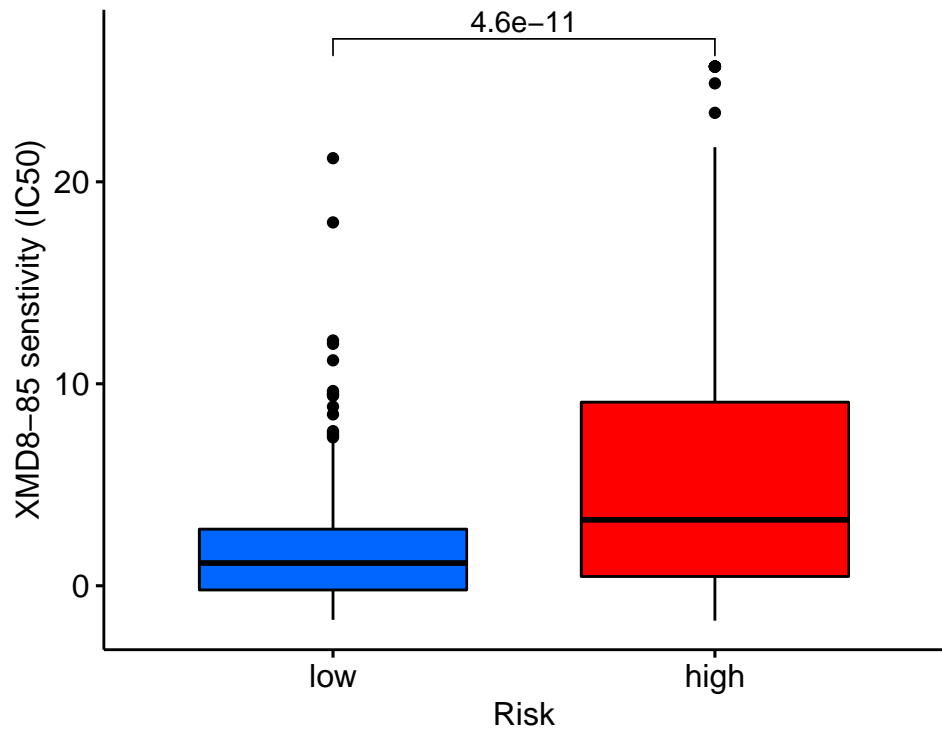

Supplement: Supplementary data 5 [file mmc5.zip › durgSenstivity.XMD8-85.pdf]

Risk 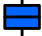 low 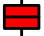 high

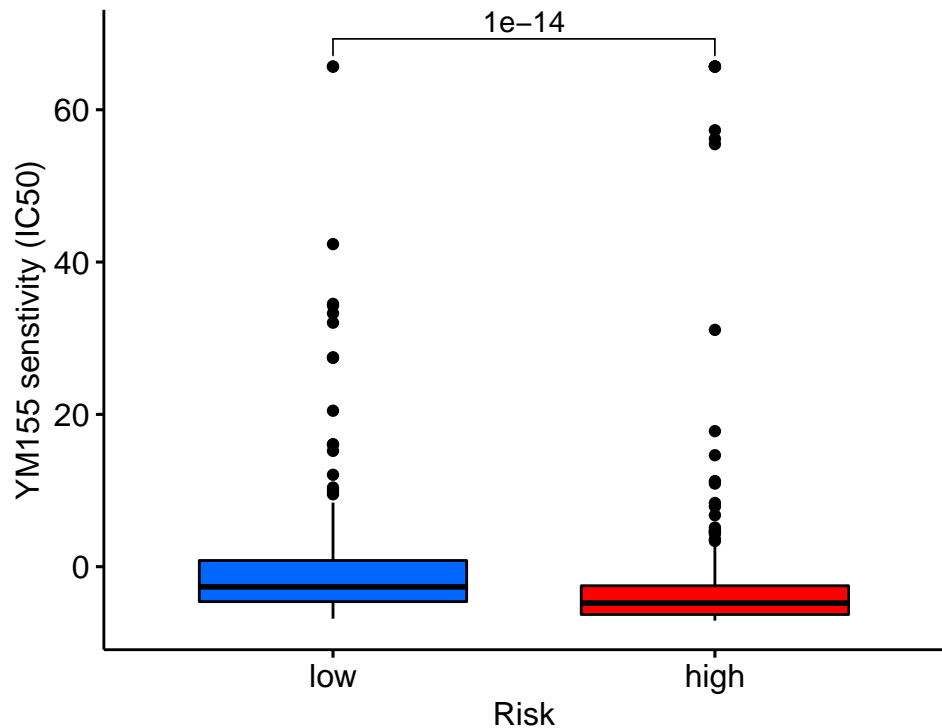

Supplement: Supplementary data 5 [file mmc5.zip › durgSenstivity.YM155.pdf]

Risk 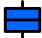 low 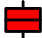 high

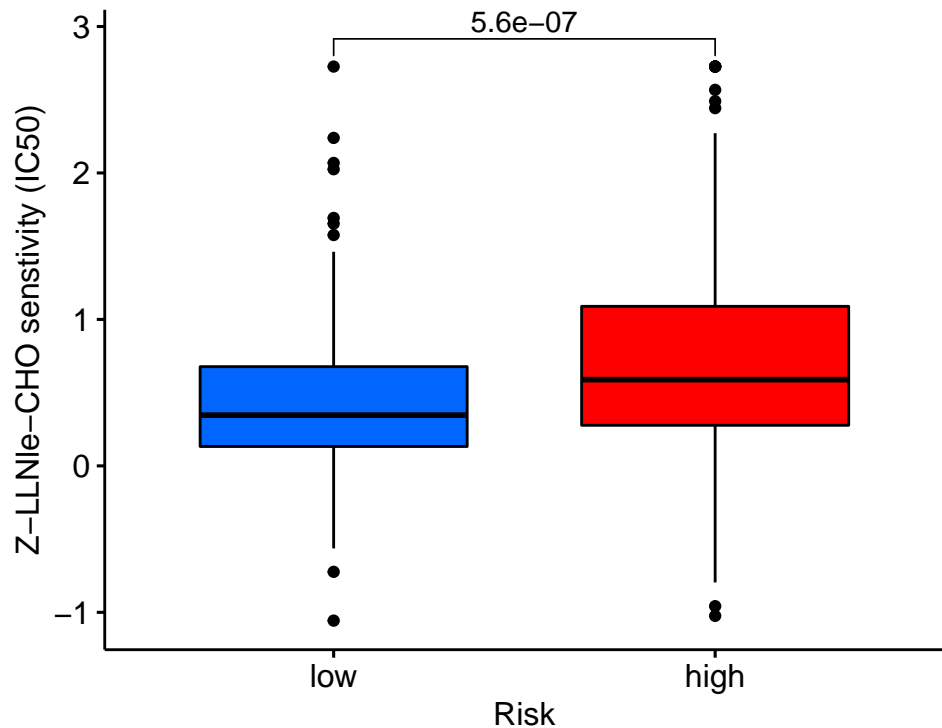

Supplement: Supplementary data 5 [file mmc5.zip › durgSenstivity.Z-LLNle-CHO.pdf]

ZSTK474 sensitivity (IC50)

Risk 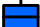 low 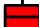 high

$6.5e-16$

low

high

Risk

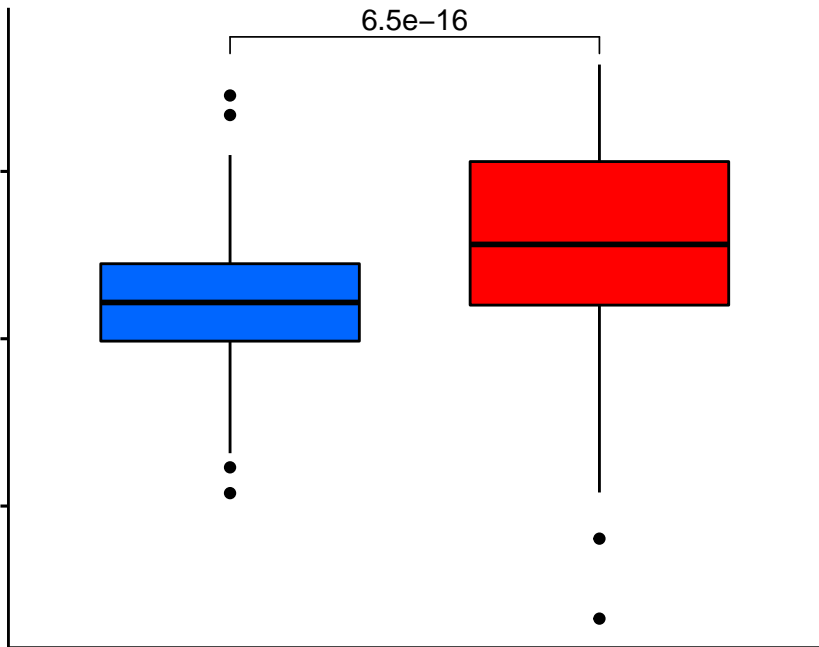

Supplement: Supplementary data 5 [file mmc5.zip › durgSenstivity.ZSTK474.pdf]
